# Force-Accelerated Ring Opening of Episulfide by Pulsed Ultrasonication

**DOI:** 10.1021/acs.macromol.5c00768

**Published:** 2025-06-16

**Authors:** Chun-Hao Ko, Hsi-Chih Wang, Van-Sieu Luc, Chun-Yi Hsu, Yangju Lin, Yu-Wen Huang, Chia-Chih Chang

**Affiliations:** a Department of Applied Chemistry, 34914 National Yang Ming Chiao Tung University, Hsinchu 300093, Taiwan; b Center for Emergent Functional Matter Science, 34914 National Yang Ming Chiao Tung University, Hsinchu 300093, Taiwan; c Institute of Chemistry, Academia Sinica, Taipei 11529, Taiwan; d Sustainable Chemical Science and Technology (SCST), Taiwan International Graduate Program (TIGP), Academia Sinica, Taipei 11529, Taiwan; e Department of Chemistry, 8430 University of Waterloo, Waterloo, ON N2L 3G1, Canada; f Department of Chemistry, 34881 National Tsing Hua University, Hsinchu 300093, Taiwan

## Abstract

Covalent polymer mechanochemistry is an emerging field that leverages the mechanical force transduced by polymer chains to bias or alter the reaction pathways uniquely, thereby influencing the stereoselectivity of chemical transformations. This study investigates the mechanochemical reactivities of episulfides featuring alkyl, ester, and phenyl substituents under pulsed ultrasonication, and the results demonstrate that episulfides bearing alkyl and phenyl substituents can undergo C–C bond cleavage to produce reactive intermediates that are amenable to subsequent *cis*–*trans* isomerization and cycloaddition reactions. In contrast, the episulfide with ester substituents is mechanochemically inactive.

## Graphical abstract

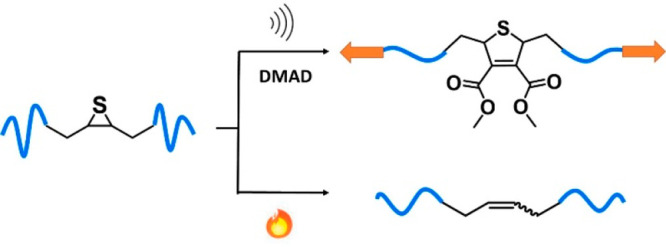

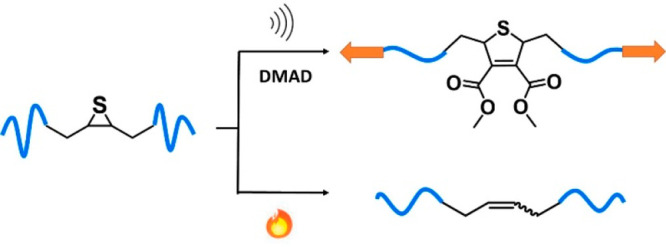

## Introduction

Mechanochemically active motifs, commonly referred to as “mechanophores”, can be activated by mechanical force to display interesting reactivities and functions, such as mechanochromism,^1,2^ flex-activation,^3^ polymer backbone remodeling,^4^ controlled release of small molecules,^5^ mechanocatalysis,^6^ gated cascade reactions,^7^ etc.^8–10^ Incorporation of mechanophores into the polymer backbone enables mechanical generation of reactive intermediates that can be trapped with appropriate reagents. Since three-membered rings are known to have sufficient ring strains to undergo ring-opening reactions in a nonscissile pathway, which allows for polymer backbone remodeling and functionalization, many mechanophore systems based on *gem*-dihalocyclopropanes,^11–13^ epoxides^14,15^ (Scheme 1A), and aziridines^16,17^ (Scheme 1B) have been investigated. Noting that ring-opened intermediates of epoxides and aziridines can be trapped by suitable dipolarphiles under pulsed ultrasonication, we are intrigued by the unexploited mechanochemical reactivities of episulfides. Moreover, thermolysis of epoxides and aziridines is known to generate carbonyl ylides and azomethine ylides,^18,19^ but thermolysis of episulfides undergoes desulfurization to generate alkenes.^20,21^ Because of the intermediacy of a ring-opened structure from carbon–carbon bond scission in the episulfide, whether the electronic structure of the mechanically generated intermediate is better described as a classical zwitterionic ylide, a 1,3-diradical, or something intermediate to the two is unknown. We will emphasize the reactivity of force-accelerated ring-opened episulfide in the later discussion.

**Scheme 1:**
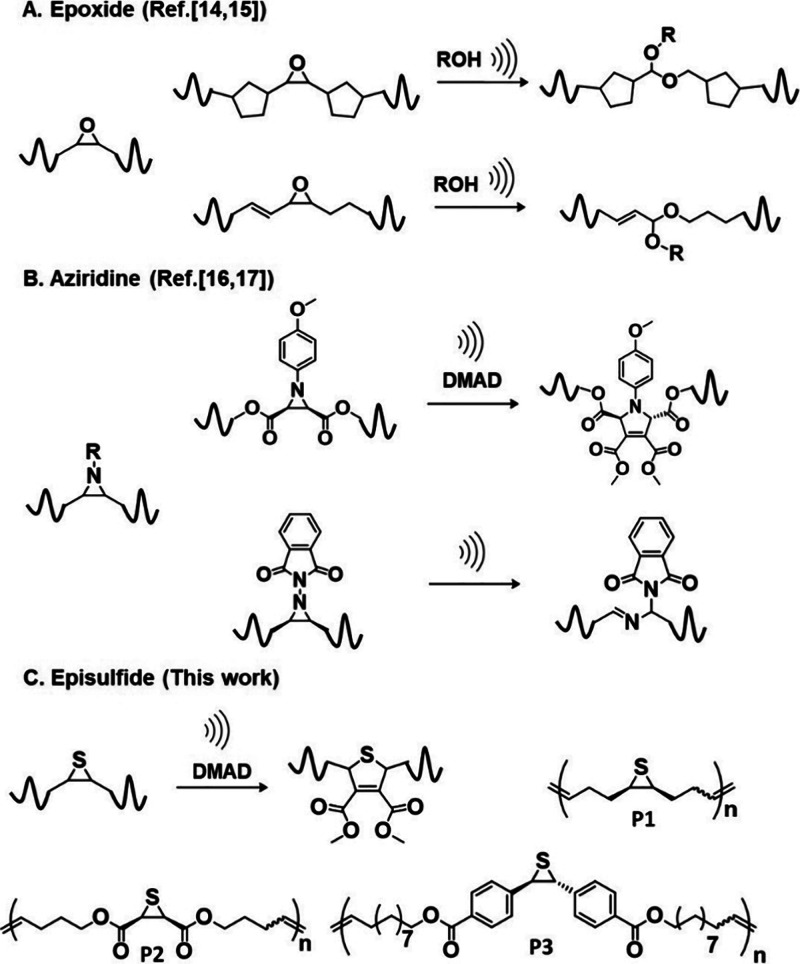
(A) Examples of Epoxides Undergoing an Addition Reaction with the Hydroxyl Group under Pulsed Ultrasound. (B) Examples of Aziridines Undergoing Cycloaddition Reaction with dimethyl acetylenedicarboxylate (DMAD) and Structural Rearrangement under Pulsed Ultrasound. (C) Evaluation of Episulfides with Different Substituents as Mechanophores

In this work, we aimed to investigate the mechanochemical reactivities of a series of episulfides featuring alkyl, phenyl, and ester tethering groups (P1–P3, Scheme 1C), unraveling the influence of these substituents on the mechanochemical reactivities. Pulsed ultrasonication was employed to apply tension along the polymer backbone, and dimethyl acetylenedicarboxylate (DMAD) and *N*-phenylmaleimide were added to investigate whether cycloaddition reactions can occur. Our results show that alkyl- and phenyl-substituted episulfides are mechanochemically active, while the one with the ester substituent is mechanochemically inactive.

## Results and Discussion

The Constrained Geometries Simulate External Force (CoGEF) method, a powerful and accessible tool to prescreen the mechanochemical reactivity at the B3LYP/6-31G* level, was employed to predict the maximum force required to induce bond scission.^22,23^ Episulfides with alkyl, ester, and phenyl substituents were chosen to study the influence of these substituents. The results are summarized in Figure 1 and Figure S1. The calculation results reveal that episulfide with substituents in the *trans*-configuration generally requires a larger force to undergo ring-opening reaction than episulfide with substituents in the *cis*-configuration. Alkyl substituents in the *trans*-configuration require a *F*_max_ of 6.11 nN, which is unlikely to undergo force-accelerated ring-opening reaction. By contrast, episulfide with alkyl substituents in the *cis*-configuration requires a *F*_max_ of 5.68 nN, suggesting that this mechanophore can possibly undergo ring-opening reaction under applied tension. Figure S1 shows that bond scission did not occur at the C–C bond of episulfide when the ester substituents are in the *trans*-configuration and the corresponding *F*_max_ is estimated to be 5.96 nN. Additionally, episulfide with ester substituents in the *cis* configuration has a lower *F*_max_ of 5.85 nN. Phenyl-substituted episulfides have *F*_max_ values of 4.03 and 5.10 nN for the *cis* and *trans* derivatives, respectively. Taken together, CoGEF calculations suggest that episulfides featuring substituents in *cis*-configurations are more likely to be mechanochemically active. Among all the possible episulfide derivatives, alkyl-substituted episulfides in the *trans*-configuration and ester-substituted episulfides are least likely to be mechanochemically active because of their high *F*_max_ values, whereas phenyl-substituted episulfide is anticipated to be mechanochemically active.

**Figure 1:**
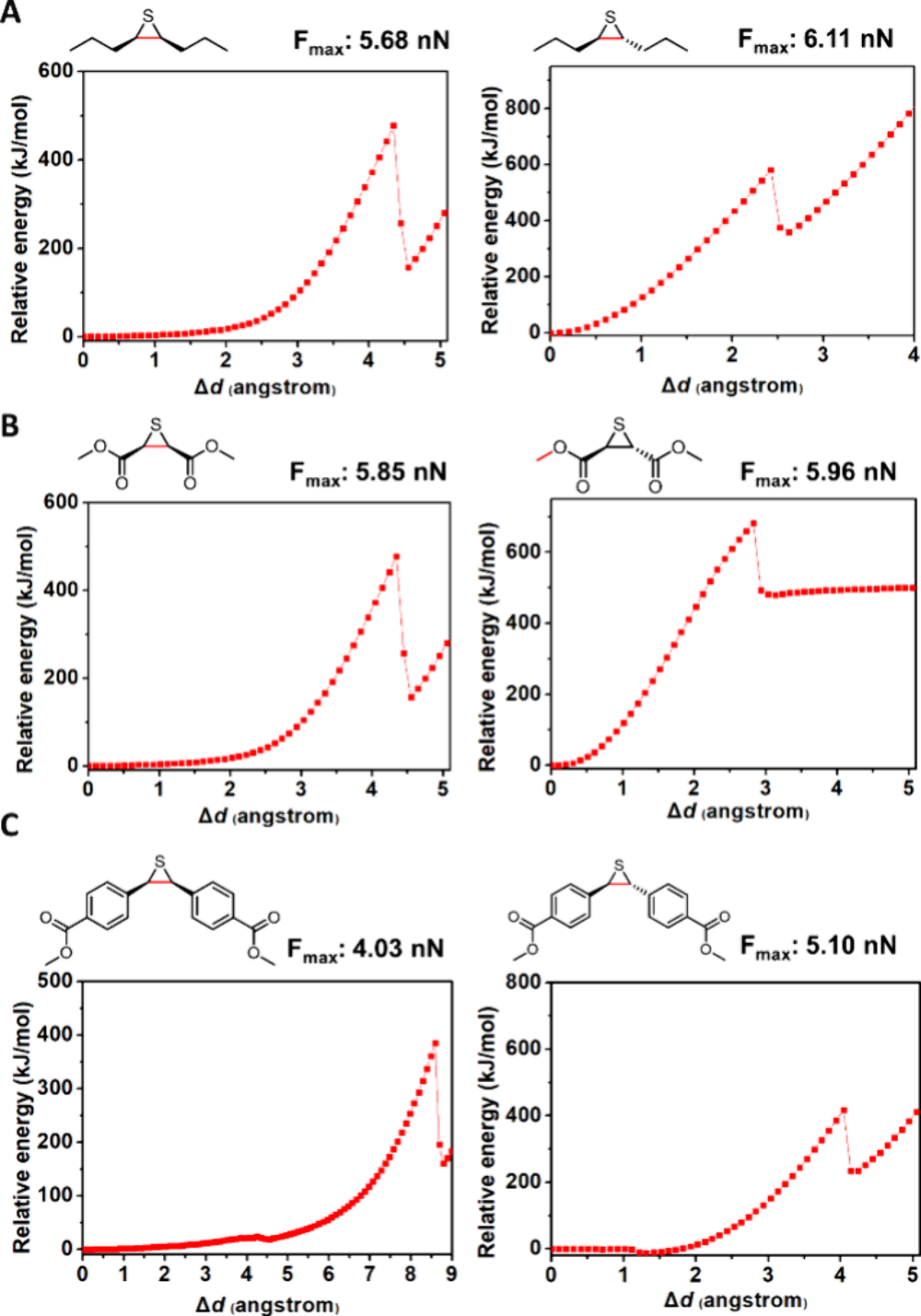
Plots of relative energy as a function of the change in bond distance relative to the force-free equilibrium geometry. The red line is the predicted location of bond breakage in the structure: (A) *cis*-alkyl and *trans*-alkyl, (B) *cis*-ester and *trans*-ester, and (C) *cis*-phenyl and *trans*-phenyl-substituted episulfides.

The next challenge is to identify suitable synthetic strategies for obtaining monomers to afford polymers P1–P3 (structures are shown in Scheme 1C) so that the CoGEF results can be validated with experimental evidence. The episulfides of interest were synthesized from the corresponding epoxides by using triphenylphosphine sulfide to replace oxygen with sulfur.^24^ Detailed experimental procedures can be found in the Supporting Information. The monomer for P1 was synthesized from 9-oxabicyclo[6.1.0]­non-4-ene in 40% yield. For the ester-substituted P2, epoxidation of dimethyl maleate with mCPBA was unsuccessful, so we had to devise an alternative oxidation procedure by using *n*-BuLi and *tert*-butyl hydroperoxide as oxidants to generate epoxide featuring ester substituents. The epoxidized dimethyl maleate was then subjected to transesterifcation with 4-penten-1-ol followed by ring-closing metathesis and oxygen-to-sulfur transformation to afford the macrocyclic monomer for P2 in 40% yield. Due to the synthetic difficulty in obtaining precursors for synthesizing the episulfide with phenyl substituents in the *cis*-conformation, the episulfide with phenyl substituents in the *trans*-conformation was pursued. Despite the fact that the epoxide featuring phenyl substituents in the *trans*-conformation can only be obtained in 28% yield even after extensive optimizations, the monomer for P3 was successfully synthesized in 29% yield by ring-closing metathesis. 10-Undecen-1-ol was used instead of 4-penten-1-ol because longer tethered alkenes can avoid the formation of the dimer. Entropy-driven ring-opening metathesis polymerization was employed to afford P1 containing *cis*-alkyl episulfide (P1, *M*_n_: 96 kDa, *Đ*: 1.78), P2 containing *cis*-ester episulfide (*M*_n_: 100 kDa, *Đ*: 1.85), and P3 containing *trans*-phenyl episulfide (*M*_n_: 220 kDa, *Đ*: 2.0) groups by using the third-generation Grubbs catalyst. The corresponding protons of these episulfides were detected at 2.95, 3.61, and 3.96 ppm, respectively. Figure S2 shows the scheme of pulsed ultrasonication experiments performed on P1–P3, and the expected polymer structures obtained after pulsed ultrasonication are labeled accordingly. We note that P2 was obtained as a copolymer with *cis*-cyclooctene with a feed molar ratio of 1:1 because it was difficult to obtain the episulfide-containing macrocyclic monomer for P2 in high yield.

After P1 was subjected to pulsed ultrasonication (10.2 W cm^–2^, 1 s on and 1 s off), a new peak at 2.63 ppm was observed for P1–1 in the ^1^H NMR spectrum (Figure S3), which can be ascribed to episulfide with alkyl substituents in the *trans* conformation. Previous work reported that the chemical shift of *trans*-5-decene sulfide is at 2.62 ppm, thus confirming the occurrence of *cis*-to-*tran*s isomerization during pulsed ultrasonication.^25^ Approximately 3% of *cis*-alkyl episulfide has isomerized to *trans*-alkyl episulfide due to the formation of a reactive intermediate during ultrasonication. We acknowledge that the extent to which the intermediate exhibits diradical versus zwitterionic ylide character remains unknown. To investigate the reactivity of mechanically generated intermediate, P1 was subjected to ultrasonication in the presence of excessive dimethyl acetylenedicarboxylate (DMAD) for 2 h, affording P1–2. After sonication, the polymer molecular weight decreased to 57 kDa (Table S1 and Figure S9B), which corresponds to an average scission cycle of 0.75. The scission cycle was calculated from the polymer molecular weight before and after sonication with the equation ln­(*M*_n,0_/*M*_n,t_)/ln­(2) to provide a quantitative description of how many scission events have occurred.^11^ Figure 2 shows two new prominent peaks emerged at 3.79 and 4.34 ppm, which can be assigned to new protons from the newly formed cycloaddition adduct of DMAD and the force generated intermediate via pulsed ultrasonication. The observed chemical shifts are in good agreement with the reported values by Kellogg and co-workers, where they had made a similar derivative.^21^ By comparing the relative integration values of new peaks to the backbone alkene peaks at 5.53 and 4.48 ppm, around 4% of the episulfide has participated in cycloaddition reaction with DMAD (Figure S18). A trace amount of episulfide has isomerized to the *trans* conformation that is difficult to quantify due to the noise from the baseline.^13^ The ^13^C NMR of P1–2 further confirmed the formation of the cycloaddition adduct with the new peaks at 164.93, 141.49, 53.86, and 52.61 ppm (Figure S19). Furthermore, we carried out a sonication experiment on a low-molecular-weight P1, P1_low_, with a *M*_n_ of 17 kDa in the presence of excess DMAD under the same conditions. Although there was no significant change in polymer molecular weight before and after sonication, as determined by GPC, a small peak was still observed at 3.79 ppm in the ^1^H NMR spectrum (Figure S4). We found that the extent of cycloaddition was far less than 1% based on the integration ratio of the cycloaddition adduct peak at 3.79 ppm and the backbone olefinic peaks at 5.53 and 5.48 ppm because a small fraction of polymer still exceeds the limiting molecular weight so that pulsed ultrasonication still can induce the formation of reactive intermediate. A previous report by Craig and co-workers reported that epoxidized polybutadiene was unable to undergo force accelerated ring-opening due to a high energy barrier of 65 kcal/mol,^14^ and poly­(episulfide) featuring alkyl substituents reported in this work proves to be mechanochemically active. For epoxidized polynorbornene with 6% *trans*-epoxide, pulsed ultrasonication increased the *trans*-epoxide content to 11% as the polymer molecular weight decreased to 48 from 965 kDa.^14^ P1 exhibits some notable mechanochemical reactivity despite the fact that cyclopentyl tethers are absent. To investigate whether P1 can undergo cycloaddition reaction with DMAD by means of heating, a 2 mg/mL solution of P1 in toluene containing 0.2 M DMAD was refluxed for 24 h. The ^1^H NMR spectrum of the recovered polymer shows new signals at 5.42 5.36, and 2.10 ppm; however, the peaks of the cycloaddition adduct that should appear at 3.79 and 4.34 ppm are absent. Given the instability of episulfides at high temperatures, we speculated that these new signals that originated from partial desulfurization of P1 are anticipated to produce polycyclooctadiene (PCOD). Indeed, additional signals in the spectrum of the heated P1 matched with the signals of PCOD as shown in Figure S5, suggesting that P1 and DMAD do not undergo cycloaddition reaction under the heat, and desulfurization reaction is preferred. A portion of episulfide in P1 desulfurized to form alkenes upon heating, while approximately 54 mol % episulfide remained intact.

**Figure 2:**
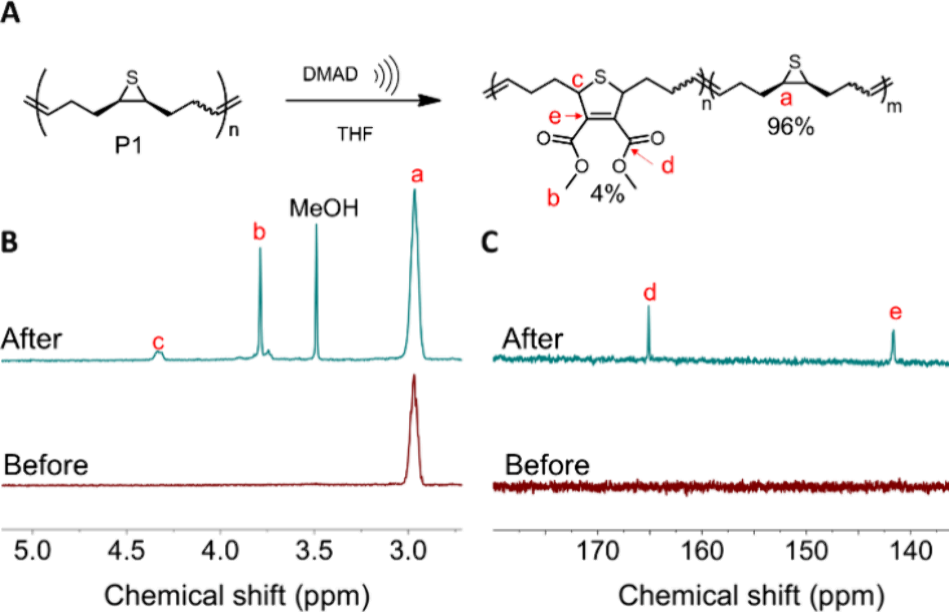
(A) Proposed cycloaddition reaction of P1 with DMAD under pulsed ultrasound. (B) ^1^H NMR spectra of P1 before sonication (bottom) and after sonication (top), and (C) ^13^C NMR spectra of P1 before sonication (bottom) and after sonication (top).

Ester-substituted episulfide containing polymer P2 was subjected to the pulsed ultrasonication with DMAD; however, there were no new signals after sonication despite the molecular weight decreasing to 58 from 100 kDa, indicating that the C–C bond of episulfide is likely as strong as the C–C bonds present in the polymer backbone (Figures S30 and S32). Considering that the CoGEF calculation result shows a high *F*_max_ of 5.85 nN for *cis*-ester-substituted episulfide, it was not surprising that ester-substituted episulfide did not undergo ring-opening reaction. Another possibility is that the lifetime of reactive intermediate produced by mechanochemical activation is too short for cycloaddition or isomerization to occur. On the contrary, ester-substituted dichlorocyclopropane can undergo ring-opening reaction to produce the corresponding rearranged products,^13^ while ester-substituted aziridine can undergo cycloaddition reaction with DMAD through an ylide-free mechanism during pulsed ultrasonication.^16^

Unlike P1 and P2 featuring substituents in the *cis-*conformation, P3 has phenyl substituents in the *trans-*conformation owing to the accessibility of such a monomer. Pulsed ultrasonication of P3 resulted in the appearance of three new ^1^H NMR at 7.82, 7.29, and 4.22 ppm, which are close to the characteristic peaks reported for *cis*-phenyl-substituted episulfides.^26^ The proton of *cis*-phenyl episulfide is located more upfield than *trans*-phenyl episulfide. Notably, the extent of *trans*-to-*cis* isomerization has reached approximately 15%. The increase in isomerization can be attributed to the larger molecular weight of P3, which in turns, allows for the scission cycle to reach to 1.67. Such a value is 2.2 times more than the scission cycle experienced by P1. Furthermore, the adjacent phenyl rings are likely to be responsible for a higher extent of isomerization. In addition, the electron resonance of the phenyl group could potentially stabilize the intermediate, thereby lowering the activation energy required for ring-opening. Once the episulfide ring is being pulled open, there are two potential pathways (i.e., diradical or ylide equivalent) to reconstruct the epsulfide, affording episulfides with either *trans* or *cis* conformation. We did not further investigate the reaction mechanism because the diradical and zwitterionic representations are resonance forms of a single intermediate, making proving or disproving the particular pathway more challenging. To investigate the reactivity of the transient intermediate toward cycloaddition, a sonication experiment of P3 with excessive DMAD was performed; a new signal emerged at 3.61 ppm, indicating that P3 can undergo addition with a dipolarophile under mechanical force. However, only about 2% of the episulfide underwent the cycloaddition reaction, and approximately 14% of *trans*-episulfide was converted to *cis-*episulfides based on the integration ratio at 5.37 ppm (backbone alkene) and 4.30 ppm (*cis*-episulfide) (Figure S53). We then employed *N*-phenyl maleimide as an alternative dipolarophile to trap the intermediate formed during the pulsed ultrasonication. The peak located at 7.16 ppm was observed in the ^1^H NMR spectrum, which can be assigned to protons on the phenyl ring in the cycloaddition adduct (Figure 3C), and the extent of cycloaddition was determined to be 12% by comparing the integration values of the peak at 7.16 ppm to the olefins present along the polymer backbone. Peaks located at 5.00 and 3.86 ppm further suggest successful cycloaddition with *N*-phenyl maleimide. The C=O signal at 172.77 ppm, and the five-membered adduct signal comes from at 53.03 and 55.08 ppm in the ^13^C NMR spectrum provides additional supporting evidence for successful cycloaddition reaction (Figure S6).

**Figure 3:**
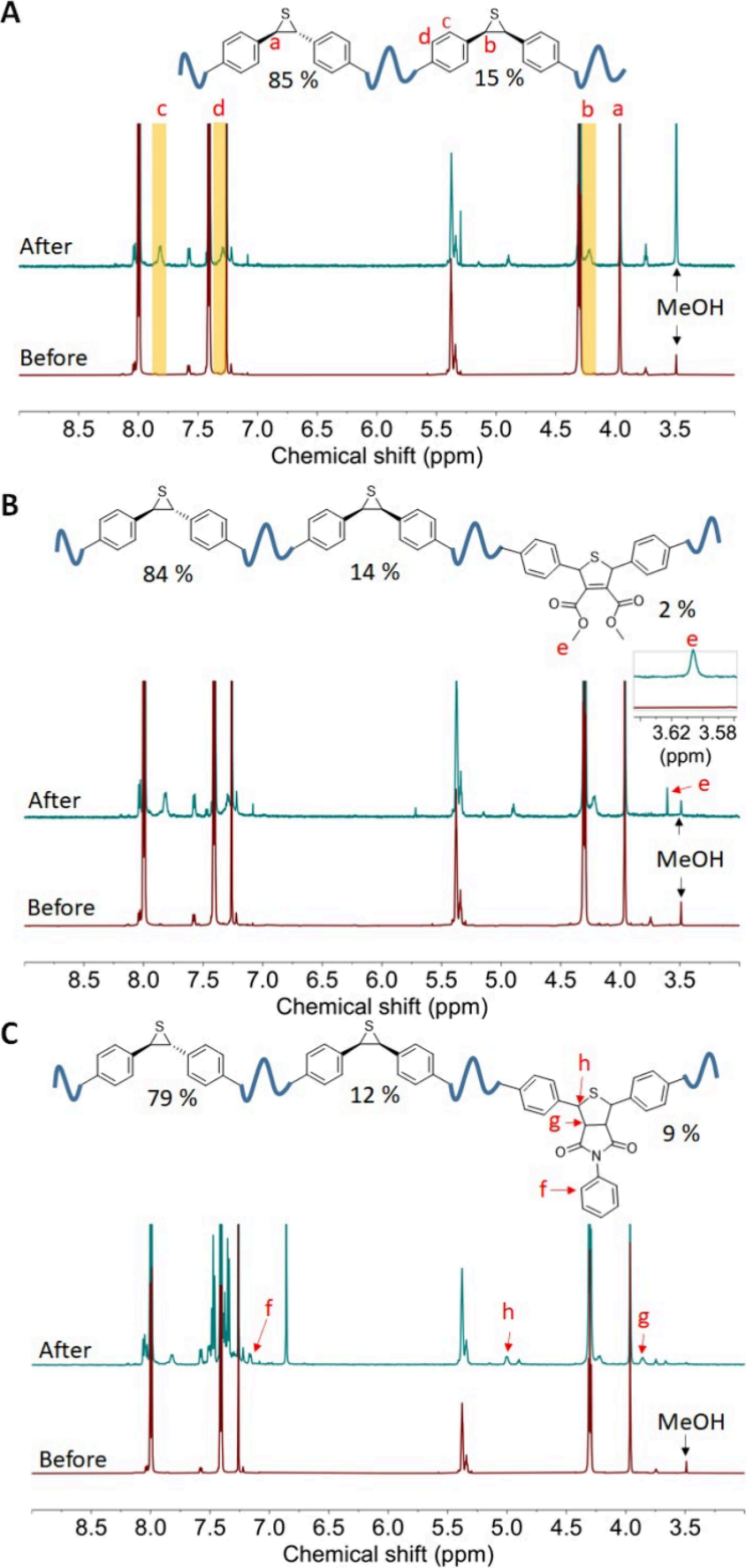
(A) ^1^H NMR spectra of P3 before sonication (bottom) and after sonication (top). (B) ^1^H NMR spectra of P3 before sonication (bottom) and after sonication with DMAD (top). (C) ^1^H NMR spectra of P3 before sonication (bottom) and after sonication with *N*-phenylmaleimide (top).

Control experiments were conducted to confirm that the ring-opening of phenyl substituted episulfides is indeed induced by a polymer transduced mechanical force. To this end, a small molecule episulfide with phenyl substituents in *trans*-conformation, compound 20, was subjected to pulsed ultrasonication and heating. After sonicating compound 20 with 24 mg of *N*-phenyl maleimide, no new signals appeared near 7.16 ppm in the ^1^H NMR spectrum as shown in Figure S7, indicating the absence of cycloaddition reaction. For the thermolysis experiment, 1 mg of compound 20 and 17 mg of *N*-phenyl maleimide were dissolved in 0.5 mL of d8-toluene and refluxed for 24 h. The ^1^H NMR spectrum shown in Figure S8 remained unchanged before and after heating. Unlike alkyl substituted episulfides, phenyl substituted episulfides did not undergo desulfurization and remained stable in refluxing toluene. Sonication experiments in the presence of 32 mM radical scavenger coumarin–2,2,6,6-tetramethylpiperidine-1-oxyl were also performed. Despite the UV detector showing that the absorbance at 335 nm of both P1 and P3 increased gradually during sonication (Figure S10), the lack of characteristic coumarin signal in the photodiode array detector suggests that the radical scavenger did not react with the transient intermediates formed during pulsed ultrasonication.

## Conclusions

In summary, we show that episulfide-containing polymers with appropriate substituents can be mechanically converted into reactive intermediates upon application of mechanical force, allowing for facile *cis*–*trans* isomerization and cycloaddition reactions. The experimental results corroborate predictions from the CoGEF calculations. Unlike epoxidized polybutadiene, which is reluctant to undergo mechanochemical activation by pulsed ultrasonication, alkyl-substituted episulfides can undergo both isomerization and cycloaddition reactions, whereas ester-substituted episulfides are mechanochemically inert. We anticipate that other yet to-be-explored mechanophore systems may benefit from having phenyl substituents either by enhanced force coupling or electronic stabilization of intermediate, resulting in an increase in the extent of mechanochemical ring-opening reactions. The diradical or zwitterionic ylide nature of the reactive intermediates derived from the episulfides studied in this work remained unclear, which warrants further experimental studies and theoretical calculations. This work contributes to the expanding landscape of mechanophores by introducing episulfides as a new class of mechanophores.

## Supplementary Material



## Notes

Experimental details and additional characterization data, kinetic modeling, synthetic procedures, CoGEF calculations, GPC chromatograms, and NMR spectra (PDF)

## References

[1] Davis D. A., Hamilton A., Yang J. L., Cremar L. D., Van Gough D., Potisek S. L., Ong M. T., Braun P. V., Martinez T. J., White S. R. (2009). Force-induced activation of covalent bonds
in mechanoresponsive polymeric materials. Nature.

[2] Kim T. A., Robb M. J., Moore J. S., White S. R., Sottos N. R. (2018). Mechanical
Reactivity of Two Different Spiropyran Mechanophores in Polydimethylsiloxane. Macromolecules.

[3] Larsen M. B., Boydston A. J. (2013). “Flex-Activated”
Mechanophores: Using
Polymer Mechanochemistry To Direct Bond Bending Activation. J. Am. Chem. Soc..

[4] Kean Z. S., Craig S. L. (2012). Mechanochemical
remodeling of synthetic polymers. Polymer.

[5] Diesendruck C. E., Steinberg B. D., Sugai N., Silberstein M. N., Sottos N. R., White S. R., Braun P. V., Moore J. S. (2012). Proton-Coupled
Mechanochemical Transduction: A Mechanogenerated Acid. J. Am. Chem. Soc..

[6] Piermattei A., Karthikeyan S., Sijbesma R. P. (2009). Activating catalysts with mechanical
force. Nat. Chem..

[7] Wang J., Kouznetsova T. B., Boulatov R., Craig S. L. (2016). Mechanical gating
of a mechanochemical reaction cascade. Nat.
Commun..

[8] Bowser B. H., Craig S. L. (2018). Empowering mechanochemistry
with multi-mechanophore
polymer architectures. Polym. Chem..

[9] Chen Y., Mellot G., van Luijk D., Creton C., Sijbesma R. P. (2021). Mechanochemical
tools for polymer materials. Chem. Soc. Rev..

[10] Li J., Nagamani C., Moore J. S. (2015). Polymer
Mechanochemistry: From Destructive
to Productive. Acc. Chem. Res..

[11] Lenhardt J. M., Black A. L., Craig S. L. (2009). gem-Dichlorocyclopropanes as Abundant
and Efficient Mechanophores in Polybutadiene Copolymers under Mechanical
Stress. J. Am. Chem. Soc..

[12] Klukovich H. M., Kouznetsova T. B., Kean Z. S., Lenhardt J. M., Craig S. L. (2013). A backbone
lever-arm effect enhances polymer mechanochemistry. Nat. Chem..

[13] Wang Z., Kouznetsova T. B., Craig S. L. (2022). Pulling Outward but Reacting Inward:
Mechanically Induced Symmetry-Allowed Reactions of cis-and trans-Diester-Substituted
Dichlorocyclopropanes. Synlett.

[14] Klukovich H. M., Kean Z. S., Ramirez A. L. B., Lenhardt J. M., Lin J., Hu X., Craig S. L. (2012). Tension Trapping of Carbonyl Ylides Facilitated by
a Change in Polymer Backbone. J. Am. Chem. Soc..

[15] Barbee M.
H., Wang J., Kouznetsova T., Lu M., Craig S. L. (2019). Mechanochemical
Ring-Opening of Allylic Epoxides. Macromolecules.

[16] Jung S., Yoon H. J. (2020). Mechanical Force Induces Ylide-Free Cycloaddition of
Nonscissible Aziridines. Angew. Chem., Int.
Ed..

[17] Jung S., Yoon H. J. (2021). Mechanical Force for the Transformation of Aziridine
into Imine. Angew. Chem., Int. Ed..

[18] Huisgen R. (1977). Electrocyclic
Ring Opening Reactions of Ethylene Oxides. Angew.
Chem., Int. Ed..

[19] Dauban P., Malik G. (2009). A masked 1,3-dipole revealed from aziridines. Angew. Chem., Int. Ed..

[20] Lown E. M., Sandhu H. S., Gunning H. E., Strausz O. P. (1968). Reactions of sulfur
atoms. XI. Intermediacy of a hybrid.pi.-thiacyclopropane in the addition
reactions to olefins and in the thermal decomposition of episulfides. J. Am. Chem. Soc..

[21] Buter J., Wassenaar S., Kellogg R. M. (1972). Thiocarbonyl ylides. Generation,
properties, and reactions. J. Org. Chem..

[22] Klein I. M., Husic C. C., Kovács D. P., Choquette N. J., Robb M. J. (2020). Validation of the CoGEF Method as a Predictive Tool
for Polymer Mechanochemistry. J. Am. Chem. Soc..

[23] Beyer M. K. (2000). The mechanical
strength of a covalent bond calculated by density functional theory. J. Chem. Phys..

[24] Chan T. H., Finkenbine J. R. (1972). Facile
conversion of oxiranes to thiiranes by phosphine
sulfides. Scope, stereochemistry, and mechanism. J. Am. Chem. Soc..

[25] Brimeyer M. O., Mehrota A., Quici S., Nigam A., Regen S. L. (1980). Silica
gel assisted synthesis of thiiranes. J. Org.
Chem..

[26] Song S.-M., Jin J., Choi J.-H., Chung W.-J. (2022). Synthesis of cis-thiiranes as diastereoselective
access to epoxide congeners via 4π-electrocyclization of thiocarbonyl
ylides. Nat. Commun..

