# Supplementary material for: Force-Accelerated Ring Opening of Episulfide by Pulsed Ultrasonication
Source: Macromolecules. 2025 Jun 16;58(13):6929–34. doi: 10.1021/acs.macromol.5c00768 (PMC12257580; doi:10.1021/acs.macromol.5c00768)
Supplement: Supplementary file 1 [file ma5c00768_si_001.pdf]

Force-accelerated Ring-Opening of Episulfide by Pulsed Ultrasonication

Chun-Hao Ko[a][b], Hsi-Chih Wang[a][b], Van-Sieu Luc[a][c][d], Chun-Yi Hsu [a][b], Yangju Lin[e], Yu-Wen Huang[f], Chia-Chih Chang[a][b]*

[a] Department of Applied Chemistry, National Yang Ming Chiao Tung University, Hsinchu 300093, Taiwan

[b] Center for Emergent Functional Matter Science, National Yang Ming Chiao Tung University, Hsinchu 300093, Taiwan

[c] Institute of Chemistry, Academia Sinica, Taipei 11529, Taiwan

[d] Sustainable Chemical Science and Technology (SCST), Taiwan International Graduate Program (TIGP), Academia Sinica, Taipei 11529, Taiwan

[e] Department of Chemistry, University of Waterloo, Waterloo, ON, Canada

[f] Department of Chemistry, National Tsing Hua University, Hsinchu 300093, Taiwan

*Email cchang113ac@nycu.edu.tw

Table of Contents

General Experimental Details 2

Reagents 2

Characterization 2

Gel permeation chromatography 2

Ultrasound Sonication 2

Experimental Procedures 3

Results and Discussion 11

Control Experiment 15

Heating Experiment 15

GPC Characterization of synthesized polymers 18

^{1}H and ^{13}C NMR spectrum 20

General Experimental Details

Reagents

All reagents were purchased from Nova-Matls, Sigma-Aldrich and TCI and used without further purification unless otherwise stated.

Characterization

NMR spectra were collected using either an Agilent 400-MR DD2 (400 MHz for ^{1}H spectra; 100 MHz for ^{13}C spectra), Agilent VNMRS 600 (600 MHz for ^{1}H spectra; 150 MHz for ^{13}C spectra), or Jeol JNM-ECZ400S/L1 (400 MHz for ^{1}H spectra; 100 MHz for ^{13}C spectra) spectrometer with chloroform-d and DMSO-d_6 as the solvent. The chemical shift (δ) are given in part per million (ppm) using the solvent peak such as chloroform-d (δ_H= 7.26 ppm; δ_C= 77.16 ppm) or DMSO-d_6 (δ_H= 2.5 ppm; δ_C= 39.52 ppm) as internal standard. The following abbreviations were used to describe the multiplicities: s = singlet, d= doublet, t = triplet, m = multiplet, ddt = double of double of triplets, etc. Coupling contanst (J) was reported in Hertz (Hz). High-resolution mass spectrometry was performed in a TOF instrument and GC-FD-MS ion source in positive ionization mode and was recorded with JEOL JMS-T200GC AccuTOF GCx at the National Yang Ming Chiao Tung University's Mass Spectrometry Facility.

Gel permeation chromatography

Gel permeation chromatography (GPC) was conducted using a JASCO instrument, equipped with an RI-4030 refractive index detector, an AS-4050 auto sampler, and a PU-4180 pump. The mobile phase was THF, and the system utilized two Waters Styragel HR4 columns in series, along with one Waters Styragel HR2 column to achieve optimal separation. The column flow rate was maintained at 1 mL/min. The number-average molecular weight (Mn) and Đ were calculated from the chromatographs with respect to polystyrene standards with M_p in the range of 980-355,000 g/mol.

Ultrasound Sonication

Ultrasonic sonication experiments using a Qsonica Q500 model. Operating at 20 kHz, we selected an amplitude of 25% and a power setting of approximately 10.2 W for the instrument. It was fitted with a titanium probe tip supplied by Sonics. Sonication experiments were performed in THF containing 250 ppm BHT at a concentration of 2 mg/mL. Before sonication, we deoxygenated the solutions for 30 minutes by bubbling nitrogen. The experiment was conducted with a controlled temperature between 5–9 °C, maintained by an ice-water bath. The sonication pulse sequence was set to 1 s on / 1 s off. The total sonication duration was set at 2 hours. For the experiments with dimethyl acetylenedicarboxylate (DMAD), DMAD was added directly to the polymer solution at a concentration of 0.2M.

Determination of Scission Cycle

Chain scission cycle is determined by the following equation:

$$\text{Chain Scission Cycle (SC)} = \frac{\ln(Mn,0)-\ln(Mn,t)}{\ln 2}$$

Mn,0 represents the initial Mn of copolymer; Mn,t represents the Mn of copolymer sonicated for t min.

CoGEF calculation

Density functional theory (DFT) calculations were conducted with Spartan'18 by using constrained geometries simulated external force (CoGEF) at the B3LYP/6-31G* level of theory in vacuum. Our aim was to model the mechanochemical behavior of episulfide with different substituents. In these calculations, we started with the equilibrium geometry of the unconstrained molecule (relative energy = 0 kJ/mol) and gradually increased the interatomic distance between two terminal carbon atoms as pointed out with arrows in Figure S1 in 0.1 Å increments for each energy minimization step until we observed an obvious change in the structural model (*i.e.,* elongation of bond). This allowed us to find the weak bond that could be broken when an external force was applied to the molecules. We were also able to obtain the bond dissociation energy (E_{max}) and the force associated with the mechanochemical transformation (F_{max}) by obtaining force versus distance plot from the first derivatives of the quadratic fit to the energy versus distance plot.[1, 2] Figure S1 shows the equilibrium geometry, structures prior to bond cleavage and structures immediately after bond cleavage, the change in distance varies from 2.53 to 8.69 angstrom depending on the substituents and stereochemistry.

Experimental Procedures

Synthetic Procedures and Characterization of Compounds

(Z)-9-oxabicyclo[6.1.0]non-4-ene (Compound 2) [3]

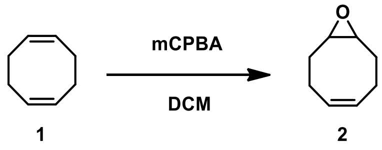

*m*CPBA (15 g, 65.4 mmol, 1.01 eq.) was dissolved in 50 mL DCM, and the solution was added dropwise to cycloocta-1,5-diene (8 g, 64.5 mmol, 1 eq.) in ice bath. After stirring overnight, aqueous $Na_2S_2O_3$ was added to the mixture to quench mCPBA, and the mixture was filtered. The solution was washed with $NaHCO_3$, and brine, the organic phase was dried with Na_2SO_4, and the crude product was purified by silica flash chromatography (5% e
thyl acetate, 95% n-Hexanes) to afford compound 2 as a colorless oil. (6.3 g, 36.6% yield).

^{1}H NMR (400 MHz, $CDCl_3$) δ: 5.69 – 5.44 (m, 2H), 3.11 – 2.95 (m, 2H), 2.53 – 2.36 (m, 2H), 2.21 – 2.09 (m, 2H), 2.09 – 1.94 (m, 4H). ^{13}C NMR (100 MHz, $CDCl_3$) δ: 128.96, 56.84, 28.23, 23.80 ppm.

(Z)-9-thiabicyclo[6.1.0]non-4-ene (Compound 3) [4]

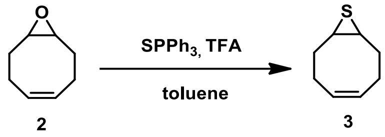

Compound 2 (1.58 g, 12.7 mmol, 1 eq.) and $SPPh_3$ (4.65 g, 19.05 mmol, 1.5 eq.) were dissolved in 62.3 mL toluene, Then TFA (0.95 ml, 12.7 mmol, 1 eq.) were added to a round bottom flask under N_2. After stirring overnight, $NaHCO_{3\,(aq)}$ was added and extracted with DCM, and the mixture was purified by silica flash chromatography (100% n-Hexanes) to afford compound 3 as a colorless oil. (0.66 g, 41.7% yield)

^{1}H NMR (400 MHz, $CDCl_3$) δ: 5.73 – 5.62 (m, 2H), 3.16 – 2.99 (m, 2H), 2.60 – 2.34 (m, 5H), 2.24 – 2.14 (m, 2H), 1.88 – 1.68 (m, 2H). ^{13}C NMR (100 MHz, $CDCl_3$) δ: 130.26, 41.39, 31.59, 26.31 ppm.

Polymerization of P1

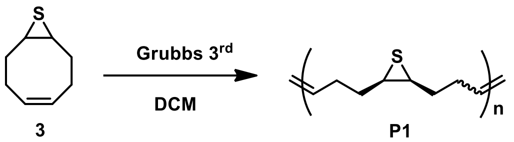

Compound 3 (0.15g, 1.1 mmol, 1100 eq.) was dissolved in 2.3 mL DCM. 4.3 mg Grubbs 3rd catalyst was dissolved in 0.5 mL DCM. 0.1 mL of the Grubbs catalyst solution (0.85 mg, 0.9 μmol, 1 eq.) was added to the solution. After stirring for 2 h, 2 mL Ethyl vinyl ether was added to terminate the reaction. After stirring for 30 min, the solution was precipitated in methanol once to obtain P1 (0.12g, 80% yield, white powder, M_n = 96 kDa, PDI =1.78).

^{1}H NMR (400 MHz, $CDCl_3$) δ: 5.59 – 5.44 (m, 2H), 3.03 – 2.92 (m, 2H), 2.39 – 2.16 (m, 4H), 1.98 – 1.85 (m, 2H), 1.57 – 1.47 (m, 2H). ^{13}C NMR (100 MHz, $CDCl_3$) δ: 130.02, 129.47, 41.51, 41.45, 32.54, 30.93, 30.82, 30.78, 27.34 ppm.

Polymerization of P1$_{LOW}$

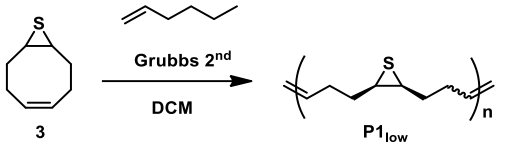

Compound 3 (0.15g, 1.1 mmol, 500 eq.) and hex-1-ene (1.3mg, 0.016mmol, 15eq.) was dissolved in 2.3 mL DCM. 4.3 mg Grubbs 2nd catalyst was dissolved in 0.5 mL DCM. 0.1 mL of the Grubbs catalyst solution (0.85 mg, 0.9 μmol, 1 eq.) was added to the solution. After stirring for 2 h, 2 mL Ethyl vinyl ether was added to terminate the reaction. After stirring for 30 min, the solution was precipitated in methanol once to obtain P1 (40 mg, 27% yield, white powder, M_n = 16 kDa, PDI =1.75).

Polymerization of PCOD

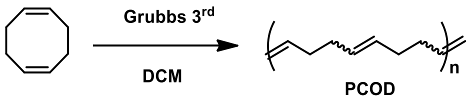

Cycloocta-1,5-diene (0.1 g, 0.93 mmol, 900 eq.) was dissolved in 0.25 mL DCM. 4.6 mg Grubbs 3rd catalyst was dissolved in 0.5 mL DCM. 0.1 mL of the Grubbs catalyst solution (0.9 mg, 0.9 μmol, 1 eq.) was added to the solution. After stirring for 2 h, 2 mL Ethyl vinyl ether was added to terminate the reaction. After 10 min, the solution was precipitated in methanol

twice to obtain product PCOD. (45 mg 45% yield, M_n=54 kDa, PDI: 1.44).

(2S,3R)-dimethyl oxirane-2,3-dicarboxylate (Compound 5) [5]

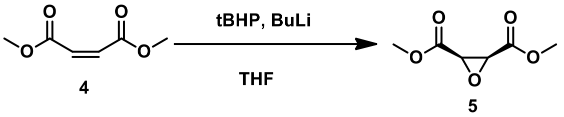

The solvent and reagent in this reaction were all dried before used, and the reaction was conducted under N_2 atmosphere. *Tert*-Butylhydroperoxide (tBHP, 4.68 g, 52 mmol, 1.5 eq., 3.5M toluene solution) and THF (50 mL) was added. The solution was cooled down to -78°C, and n-BuLi (2.4 g, 38 mmol, 1.1 eq., in 2.5M hexane solution) was added dropwise. After stirring for 15 min, dimethyl maleate (10 g, 34 mmol, 1 eq.) was added dropwise. Then the solution was warmed up to room temperature and stirred overnight. Methanol and $Na_2S_2O_3$ were added to quench the reaction. Finally, the solution was purified by silica flash chromatography (20% Ethyl acetate, 80% n-Hexanes) to afford compound 5 as a colorless oil. (2.08 g, 25% yield).

^{1}H NMR (400 MHz, $CDCl_3$) δ: 3.80 (s, 6H), 3.71 (s, 2H). ^{13}C NMR (100 MHz, $CDCl_3$) δ: 166.30, 52.95, 52.60 ppm.

(2S,3R)-di(pent-4-en-1-yl) oxirane-2,3-dicarboxylate (Compound 6)

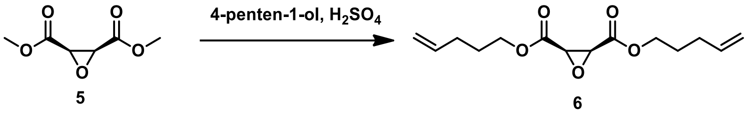

Compound 5 (1 g, 6.25 mmol, 1 eq.) was dissolved in 4-Penten-1-ol (10 mL), and sulfuric acid (1 mL, 5.5 mmol, 0.88 eq.) was added dropwise. After stirring for 2 days, $NaHCO_{3\,(aq)}$ was added and extracted with DCM, and the crude product was purified by silica flash chromatography (10% Ethyl acetate, 90% n-Hexanes) to afford products as a colorless oil. (0.9g, 53% yield).

^{1}H NMR (400 MHz, $CDCl_3$) δ: 5.77 (m, 2H), 5.10 – 4.90 (m, 4H), 4.18 (t, *J* = 6.7, 1.2 Hz, 4H), 3.68 (s, 2H), 2.16 – 2.06 .80 – 1.70 (m, 4H). ^{13}C NMR (100 MHz, $CDCl_3$) δ: 160.39, 131.76, 110.26, 60.12, 47.28, 24.46, 22.24 ppm.

(1R,14S)-3,12,15-trioxabicyclo[12.1.0]5entadic-7-ene-2,13-dione (**Compound** 7)

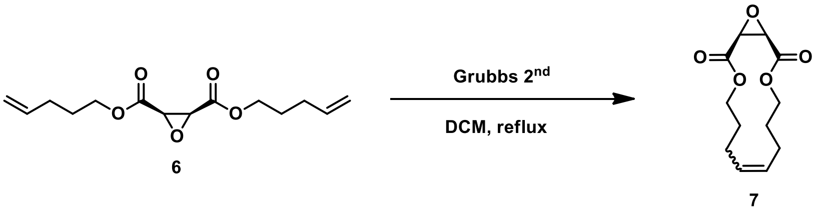

Compound 6 (0.8 g, 3 mmol, 1 eq.) was dissolved in 450 mL DCM, and sparged with N_2 for 30 min. Grubbs 2nd (12.6 mg, 0.15 mmol, 0.05 eq.) was added to the solution under N_2. The reaction was refluxed for 18 h. After the reaction was completed, 5 mL of ethyl vinyl ether was added to the mixture and stirred for 30 min. The reaction was cooled to room temperature and concentrated under reduced pressure. The residue was separated by silica gel column (10% ethyl acetate, 90% n-hexanes) and recrystallized in n-hexanes to afford compound 7 as a white powder. (0.432 g, 60% yield).

^{1}H NMR (400 MHz, $CDCl_3$) δ: 5.44 – 5.40 (m, 2H), 5.39 – 5.35 (m, 2H), 4.46 (m, 2H), 4.32 (m), 4.09 – 3.99 (m), 3.73 (s), 3.73 (s), 2.29 – 2.03 (m), 1.88 – 1.78 (m), 1.76 – 1.60 (m). ^{13}C NMR (100 MHz, $CDCl_3$) δ: 165.62, 165.37, 130.55, 129.72,

(1R,14S)-3,12-dioxa-15-thiabicyclo[12.1.0]6entadic-7-ene-2,13-dione (Compound 8)[4]

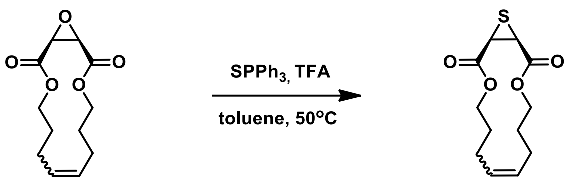

Compound 7 (0.4 g, 1.7 mmol, 1 eq.) and $SPPh_3$ (0.8 g, 2.7 mmol, 1.7 eq.) was dissolved in 5 mL toluene under N_2, then TFA (0.13 ml, 1.7 mmol, 1eq.) were added. After stirring overnight, $NaHCO_3$ was added to neutralize the solution, and the crude product was purified by silica flash chromatography (5% ethyl acetate, 95% n-hexanes) to afford compound 8 as a colorless oil. (0.17 g, 40% yield).

^{1}H NMR (400 MHz, $CDCl_3$) δ: 5.44 – 5.39 (m), 5.38 – 5.32 (m), 4.38 – 4.29 (m), 4.28 – 4.16 (m), 4.09 – 3.89 (m), 3.63 (s), 3.62 (s), 2.35 – 2.00 (m), 1.81 – 1.52 (m). ^{13}C NMR (100 MHz, $CDCl_3$) δ: 167.13, 167.03, 130.59, 129.85, 65.73, 64.45,

Polymerization of P2

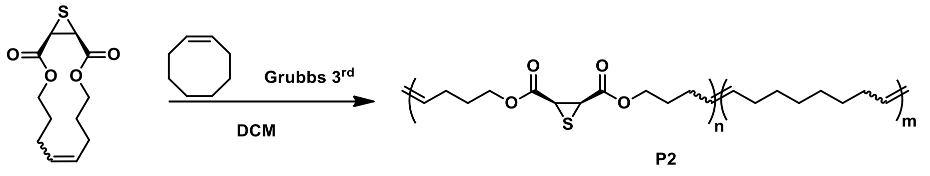

Compound 8 (66 mg, 0.257 mmol, 400 eq.) and *cis*-cyclooctene (28 mg, 0.257 mmol, 400 eq.) were dissolved in 0.2 mL DCM. 2.8 mg Grubbs 3rd catalyst was dissolved in 0.5 mL DCM. 0.1 mL of the Grubbs catalyst solution (0.57 mg, 0.68 μmol, 1 eq.) was added to the solution. After stirring for 3 h, 0.3 mL of ethyl vinyl ether was added to terminate the reaction. After stirring for 1 h, the solution was precipitated twice in methanol to obtain product P2. (30 mg, 33.3% yield, M_n =100 kDa, PDI = 1.85, white powder).

^{1}H NMR (600 MHz, $CDCl_3$) δ: 5.49 – 5.31 (m), 4.19 – 4.14 (m), 3.61 (s), 2.16 – 1.89 (m), 1.71 (t, J = 7.2 Hz), 1.35 – 1.22 (m). ^{13}C NMR (100 MHz, $CDCl_3$) δ: 167.36, 131.84, 130.43, 129.92, 128.47, 65.97, 34.65, 32.70, 29.72, 29.59, 29.14, 28.80,

4-formylbenzoic acid (Compound 10)[6]

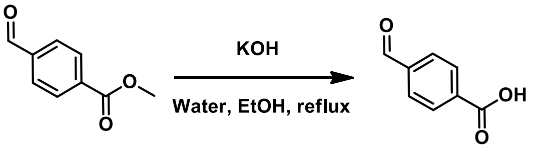

9 10

KOH (4.1 g, 73 mmol, 1.2 eq.) was added to methyl 4-formylbenzoate (10 g, 61 mmol, 1 eq.) in EtOH and H2O solution (100 mL: 25 mL), refluxed for 3 h. 1M HCl aqueous solution was added to pH = 3 and the precipitate was collected by filtration to afford compound 10 as a white powder. (7.6 g, 85% yield).

^{1}H NMR (600 MHz, d$_6$-DMSO) δ: 13.39 (s, 1H), 10.11 (s, 1H), 8.13 (d, *J* = 8.2 Hz, 2H), 8.02 (d, *J* = 8.1 Hz, 2H). ^{13}C NMR (150 MHz, d$_6$-DMSO) δ: 192.99, 166.55, 138.88, 135.62, 129.91, 129.54 ppm.

Undec-10-en-1-yl 4-formylbenzoate (Compound 11)

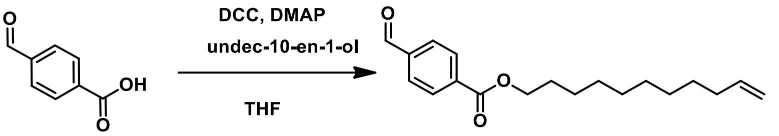

10 11

Compound 10 (5 g, 33.3 mmol, 1 eq.), 4-Dimethylaminopyridine (DMAP, 447 mg, 36.6 mmol, 0.11 eq.), and undec-10-en-1-ol (6.2 g, 36.4 mmol, 1.1 eq.) were dissolved in 150 ml THF. The solution was cooled using an ice-bath. Then N,N'-dicyclohexylcarbodiimide (DCC, 7.55 g, 2.08 mmol, 1.1 eq.) in 150 mL THF was added into the system dropwise. The mixture was stirred at room temperature overnight. Precipitated dicyclohexylurea was filtered off. The solvent was removed and the residue was separated by silica gel column (5% Ethyl acetate, 95% n-Hexanes) to afford products as a colorless oil. (10 g, 70% yield)

^{1}H NMR (400 MHz, CDCl$_3$) δ: 10.10 (s, 1H), 8.19 (d, *J* = 8.3 Hz, 2H), 7.95 (d, *J* = 8.5 Hz, 2H), 5.80 (ddt, *J* = 16.9, 10.2, 6.7 Hz, 2H), 5.04 – 4.85 (m, 2H), 4.35 (t, *J* = 6.7 Hz, 4H), 2.08 – 1.98 (m, 2H), 1.82 – 1.73 (m, 2H), 1.47 – 1.24 (m, 12H). ^{13}C NMR (150 MHz, CDCl$_3$) δ: 191.65, 165.63, 139.17, 139.07, 135.50, 130.14, 129.49, 114.14, 65.77, 33.77, 29.43, 29.37,

Methyl 4-(bromomethyl)benzoate (Compound 13)

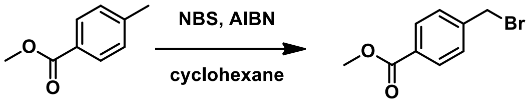

12 13

A mixture of methyl 4-methylbenzoate (22 g, 147 mmol, 1 eq.), *N*-bromosuccinimide (NBS) (26 g, 147 mmol, 1 eq.) and AIBN (1.2 g, 7.3 mmol, 0.05 eq.) in 250 mL cyclohexane was refluxed for 4h. Then the mixture was filtered and concentrated, the residue was then recrystallized with ethanol to afford compound 13 as a white powder. (13.4 g, 40% yield).

^{1}H NMR (400 MHz, CDCl$_3$) δ: 8.01 (d, *J* = 8.3 Hz, 2H), 7.46 (d, *J* = 8.3 Hz, 2H), 4.50 (s, 2H), 3.92 (s, 3H). ^{13}C NMR (150 MHz, CDCl$_3$) δ: 166.52, 142.61, 130.07, 130.05, 129.01, 52.21, 32.20 ppm.

4-(bromomethyl)benzoic acid (Compound 14)[7]

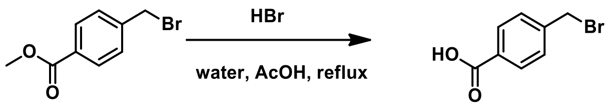

13 14

Compound 13 (5.57 g, 24.2 mmol) was dissolved in acetic acid (50 ml) and aqueous HBr (47 %, 50 ml). The solution was refluxed for 12 h and poured into 500 ml ice water slowly. The resulting precipitate was collected by filtration and washed with distilled water. The filter cake was dried in vacuum to afford compound 13 as a colorless solid (4.9 g, 95% yield).

^{1}H NMR (600 MHz, d_6-DMSO) δ: 12.98 (s, 1H), 7.90 (d, *J* = 8.3 Hz, 2H), 7.54 (d, *J* = 8.2 Hz, 2H), 4.73 (s, 2H). ^{13}C NMR (150 MHz, d_6-DMSO) δ: 166.88, 142.84, 130.52, 129.67, 129.45, 33.28 ppm.

Undec-10-en-1-yl 4-(bromomethyl)benzoate (Compound 15)

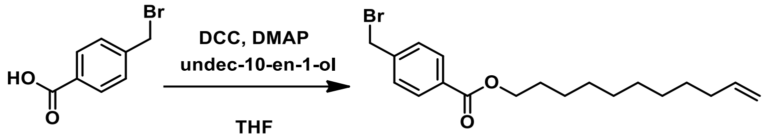

14 15

Compound 14 (6 g, 30 mmol, 1 eq.), 4-Dimethylaminopyridine (DMAP, 366 mg, 3 mmol, 0.11 eq.), and undec-10-en-1-ol (5.1 g, 30 mmol, 1.1 eq.) were dissolved in 150 ml THF. The solution was cooled using an ice-bath. Then *N,N'*-dicyclohexylcarbodiimide (DCC, 6.18 g, 30 mmol, 1.1 eq.) in 150 mL THF was added into the system dropwise. The mixture was stirred at room temperature overnight. Precipitated dicyclohexylurea was filtered off. The solvent was removed in vacuo and the residue was separated by silica gel column (3% Ethyl acetate, 97% n-Hexanes) to afford products as colorless oils. (6.14 g, 60% yield).

^{1}H NMR (400 MHz, $CDCl_3$) δ: 8.01 (d, *J* = 8.4 Hz, 2H), 7.46 (d, *J* = 8.3 Hz, 2H), 5.96 – 5.70 (m, 1H), 5.04 – 4.86 (m, 2H), 4.50 (s, 2H), 4.31 (t, *J* = 6.7 Hz, 2H), 2.07 – 1.99 (m, 2H), 1.82 – 1.70 (m, 2H), 1.42 – 1.24 (m, 12H). ^{13}C NMR (150 MHz, $CDCl_3$) δ: 166.09, 142.48, 139.19, 130.46, 130.03, 128.98, 114.12, 65.27, 33.78, 32.24, 29.44, 29.38, 29.23, 29.08, 28.90,

Di(undec-10-en-1-yl) 4,4'-((2R,3R)-oxirane-2,3-diyl)dibenzoate (Compound 16) [8]

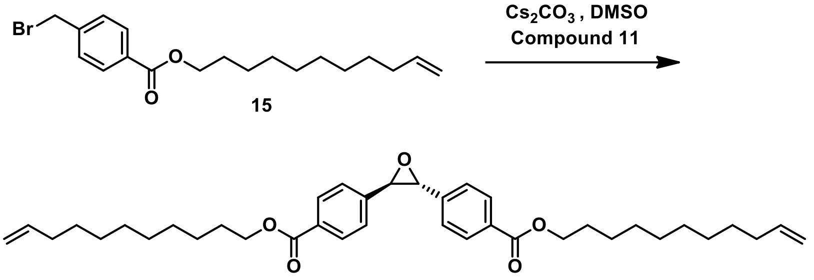

16

To a solution of compound 15 (4.5 g, 12.2 mmol, 1 eq.) and compound 11 (3.7 g, 12.2 mmol, 1 eq.) in 15 mL DMSO was added Cs_2CO_3 (4.0 g, 12.2 mmol, 1 eq.) at room temperature. After stirring for 16 h, 500 mL EtOAc was added to the reaction. The resulting mixture was washed with water, and the solvent was dried (Na_2SO_4). After evaporation, the residue was separated by silica gel column (5% Ethyl acetate, 95% n-Hexanes) to afford compound 16 as a white powder. (2.0 g, 28% yield).

^{1}H NMR (600 MHz, $CDCl_3$) δ: 8.06 (d, *J* = 8.2 Hz, 4H), 7.42 (d, *J* = 8.4 Hz, 4H), 5.89 – 5.68 (m, 2H), 5.02 – 4.85 (m, 4H), 4.32 (t, *J* = 6.7 Hz, 4\H), 3.91 (s, 2H), 2.07 – 2.00 (m, 4H), 1.77 (p, *J* = 6.9 Hz, 4H), 1.44 (m, 5H), 1.40 – 1.28 (m, 20H). ^{13}C NMR (150 MHz, $CDCl_3$) δ: 166.24, 141.52, 139.19, 130.68, 129.88, 125.42, 114.13, 65.28, 62.54, 33.78, 29.45, 29.38,

Compound 17

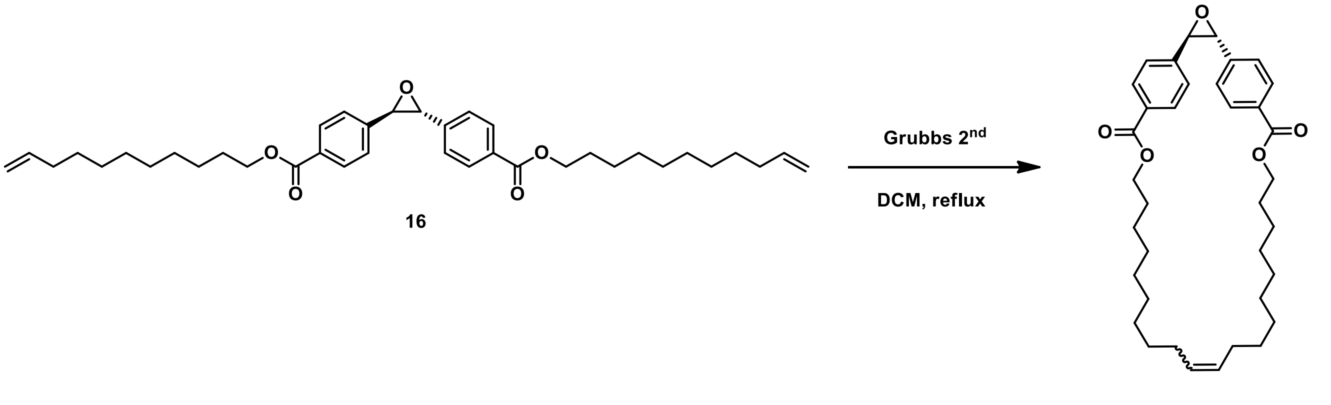

17

Compound 16 (1.5 g, 2.5 mmol, 1 eq.) was dissolved in 650 mL DCM, and sparged with N_2 for 30 min. 2nd generation Grubbs catalyst (108 mg, 0.12 mmol, 0.05 eq.) was added to the solution under N_2. The reaction was refluxed for 18 h. After the reaction was completed, 5 mL of ethyl vinyl ether was added to the mixture was and stirred for 30 min. The reaction was cooled to room temperature and concentrated under reduced pressure. The residue was separated by silica gel column (7% Ethyl acetate, 93% n-Hexanes) and recrystallized from n-hexanes to afford products as a white powder. (0.41 g, 29% yield).

^{1}H NMR (600 MHz, $CDCl_3$) δ: 8.06 (d, *J* = 8.4 Hz, 4H), 7.69 – 7.34 (m, 4H), 5.56 – 5.21 (m, 2H), 4.38 (dddd, *J* = 40.9, 10.8, 6.5, 4.2 Hz, 4H), 3.88 (s, 2H), 1.94 – 1.81 (m, 4H), 1.79 – 1.74 (m, 4H), 1.51 – 1.45 (m, 4H), 1.41 – 1.34 (m, 4H), 1.31 – 1.17 (m, 16H). ^{13}C NMR (150 MHz, $CDCl_3$) δ: 166.10, 141.57, 130.71, 130.15, 129.92, 129.89, 125.33, 65.45, 62.75,

3

2

.

9

Compound 18 [4]

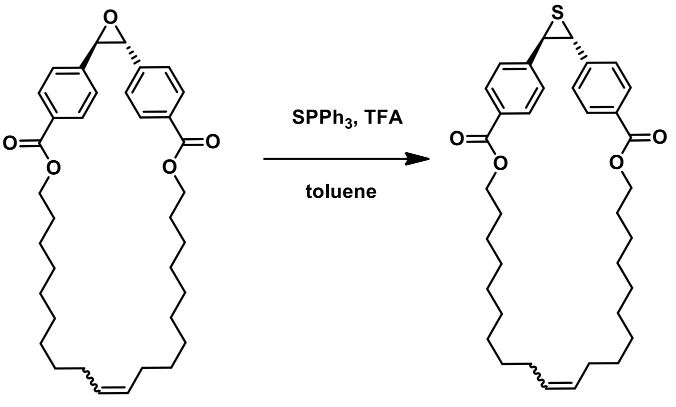

17 18

Compound 17 (0.4 g, 0.71 mmol, 1 eq.) and $SPPh_3$ (0.273 g, 0.93 mmol, 1.3 eq.) was dissolved in 5 mL toluene under N_2, and TFA (53 µl, 0.71 mmol, 1 eq.) were added. After stirring overnight, $NaHCO_3$ was added to neutralize the solution, and the crude product was purified by silica flash chromatography (50% DCM, 50% n-Hexanes) to afford products as a white powder. (0.16 g, 39% yield).

^{1}H NMR (600 MHz, $CDCl_3$) δ: 8.01 (d, *J* = 8.4 Hz, 4H), 7.42 (d, *J* = 8.4 Hz, 4H), 5.52 – 5.28 (m, 2H), 4.36 (m, 4H), 3.91 (s, 2H), 1.91 – 1.86 (m, 4H), 1.78 – 1.70 (m, 4H), 1.50 – 1.42 (m, 4H), 1.41 – 1.33 (m, 4H), 1.31 – 1.14 (m, 16H). ^{13}C NMR (150 MHz, $CDCl_3$) δ: 166.13, 143.26, 130.18, 130.15, 129.90, 126.91, 126.88, 65.31, 45.14, 32.83, 29.84, 29.74,

Polymerization of P3

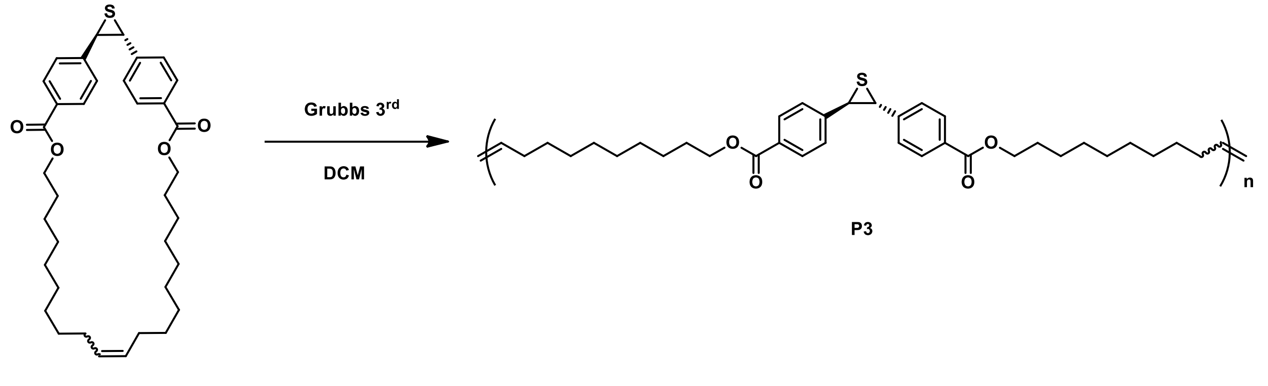

18

Compound 18 (150 mg, 0.268 mmol, 210 eq.) were dissolved in DCM (0.5 mL). 6 mg Grubbs 3rd catalyst was dissolved in 0.5 mL DCM. 0.1 mL of the Grubbs catalyst solution (1.1 mg, 0.68 µmol, 1 eq.) was added to the solution. After stirring for 3 h, 0.3 mL of ethyl vinyl ether was added to terminate the reaction. After stirring for 1 h, the solution was precipitated twice in methanol to obtain product P3. (86 mg, 57.3% yield, M_n =220 kDa, PDI =2.0, white powder).

^{1}H NMR (600 MHz, $CDCl_3$) δ: 8.00 (d, *J* = 8.3 Hz, 4H), 7.41 (d, *J* = 8.3 Hz, 4H), 5.41 – 5.31 (m, 2H), 4.30 (t, *J* = 6.7 Hz,

4H), 3.96 (s, 2H), 1.96 (d, J = 5.8 Hz, 4H), 1.80 – 1.70 (m, 4H), 1.46 – 1.38 (m, 4H), 1.36 – 1.18 (m, 23H). ^{13}C NMR (150 MHz, $CDCl_3$) δ: 166.17, 143.29, 130.32, 130.10, 129.89, 126.90, 65.23, 44.94, 32.58, 29.63, 29.48, 29.41, 29.25,

Dimethyl 4,4'-((2R,3R)-oxirane-2,3-diyl)dibenzoate (Compound 19)

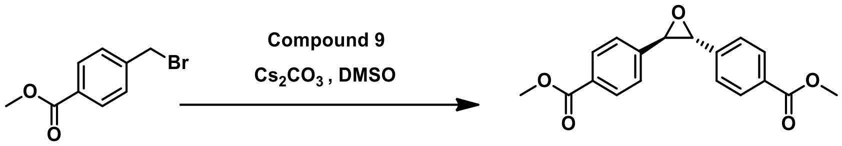

13 19

To a solution of compound 13 (4 g, 17.5 mmol, 1 eq.) and compound 9 (2.9 g, 17.5 mmol, 1 eq.) in 15 mL of DMSO was added Cs_2CO_3 (5.7 g, 17.5 mmol, 1 eq.) at room temperature. After stirring for 16 h, 500 mL EtOAc was added to the reaction. The resulting mixture was washed with water, and the solvent was dried (Na_2SO_4). After evaporation, the residue was separated by silica gel column (10% Ethyl acetate, 90% n-Hexanes) to afford compound 19 as a white powder. (1.8 g, 33% yield).

^{1}H NMR (600 MHz, $CDCl_3$) δ: 8.06 (d, J = 8.3 Hz, 4H), 7.42 (d, J = 8.3 Hz, 4H), 3.93 (s, 6H), 3.92 (s, 2H). ^{13}C NMR (150 MHz, $CDCl_3$) δ: 166.66, 141.63, 130.30, 129.93, 125.47, 62.50, 52.20 ppm.

Dimethyl 4,4'-((2R,3R)-thiirane-2,3-diyl)dibenzoate (Compound 20)

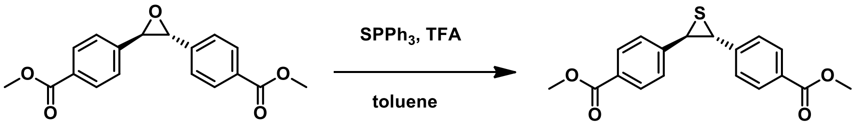

19 20

Compound 19 (0.8g, 2.5 mmol, 1 eq.) and $SPPh_3$ (0.75 g, 2.5mmol, 1.3 eq.) was dissolved in 10 mL toluene under N_2, and TFA (0.29 g, 2.5 mmol, 1eq.) were added. After stirring overnight, $NaHCO_3$ (aq) was added to neutralize the solution and extracted with DCM, and the solution was purified by silica flash chromatography (70% DCM, 30% n-Hexanes) to afford compound 20 as a white powder. (0.3 g, 36% yield).

^{1}H NMR (400 MHz, $CDCl_3$) δ: 8.00 (d, J = 8.4 Hz, 4H), 7.42 (d, J = 8.5 Hz, 4H), 3.97 (s, 2H), 3.92 (s, 6H). ^{13}C NMR (100 MHz, $CDCl_3$) δ: 166.60, 143.42, 129.94, 129.72, 126.95, 52.17, 44.90 ppm.

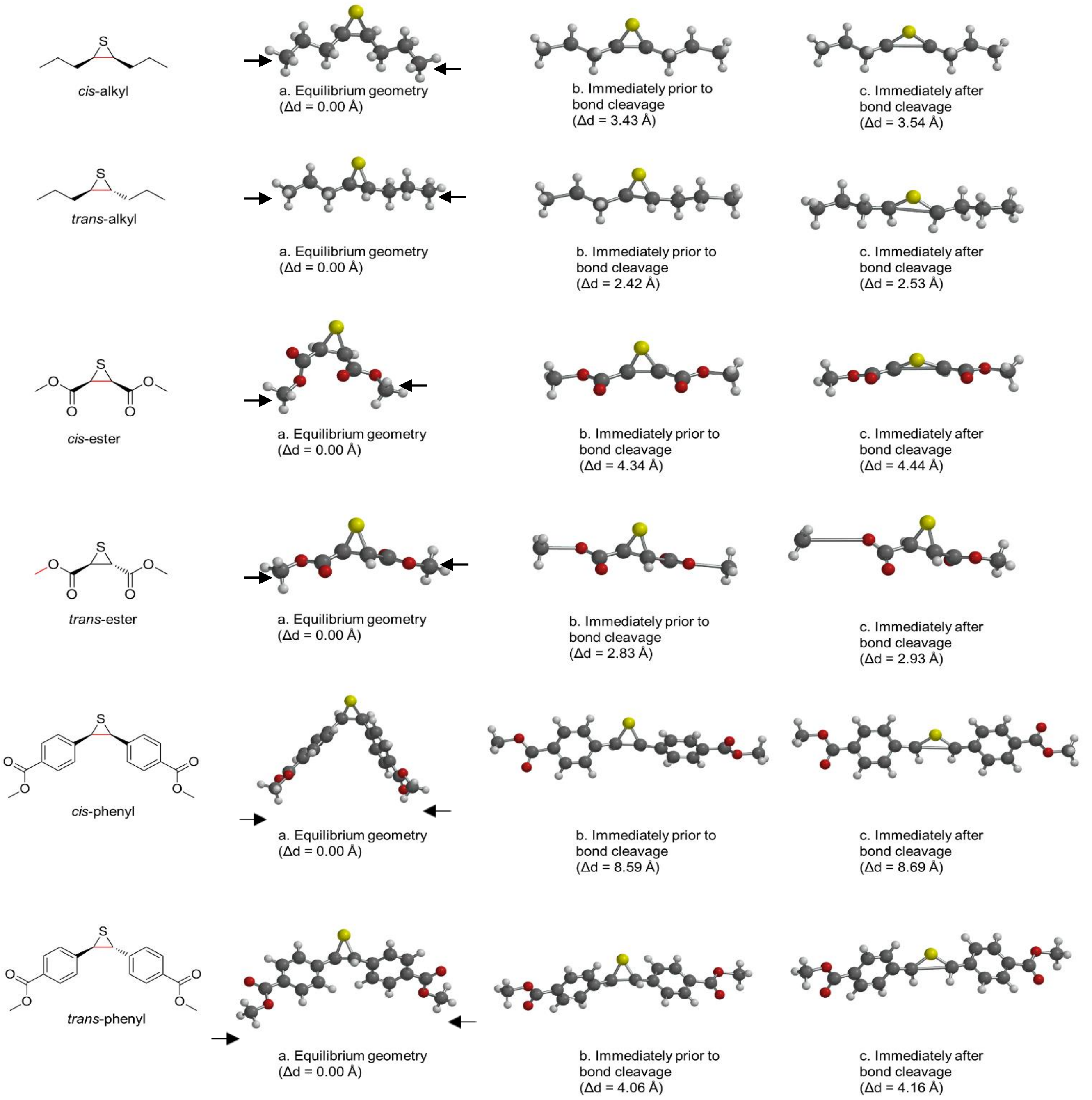

Figure S1. Summary of CoGEF simulations for episulfide with different substituents. The constraint was set between two terminal carbon atoms as indicated by the black arrows. For cis-alkyl, initial distance of equilibrium geometry is 7.748 Å, distance immediately prior to bond cleavage is 11.182 Å, distance immediately after bond cleavage is 11.283 Å, total step is 35; For trans-alkyl, initial distance of equilibrium geometry is 8.986 Å, distance immediately prior to bond cleavage is 11.410 Å, distance immediately after bond cleavage is 11.511 Å, total step is 25; For cis-ester, initial distance of equilibrium

geometry is 5.971 Å, distance immediately prior to bond cleavage is 10.314 Å, distance immediately after bond cleavage is 10.415 Å, total step is 44; For trans-ester, initial distance of equilibrium geometry is 8.332 Å, distance immediately prior to bond cleavage is 11.16 Å, distance immediately after bond cleavage is 11.261 Å, total step is 32; For cis-phenyl, initial distance of equilibrium geometry is 10.032 Å, distance immediately prior to bond cleavage is 18.618 Å, distance immediately after bond cleavage is 18.719 Å, total step is 86; For trans-phenyl, initial distance of equilibrium geometry is 15.014 Å, distance immediately prior to bond cleavage is 19.072 Å, distance immediately after bond cleavage is 19.173 Å, total step is 41;

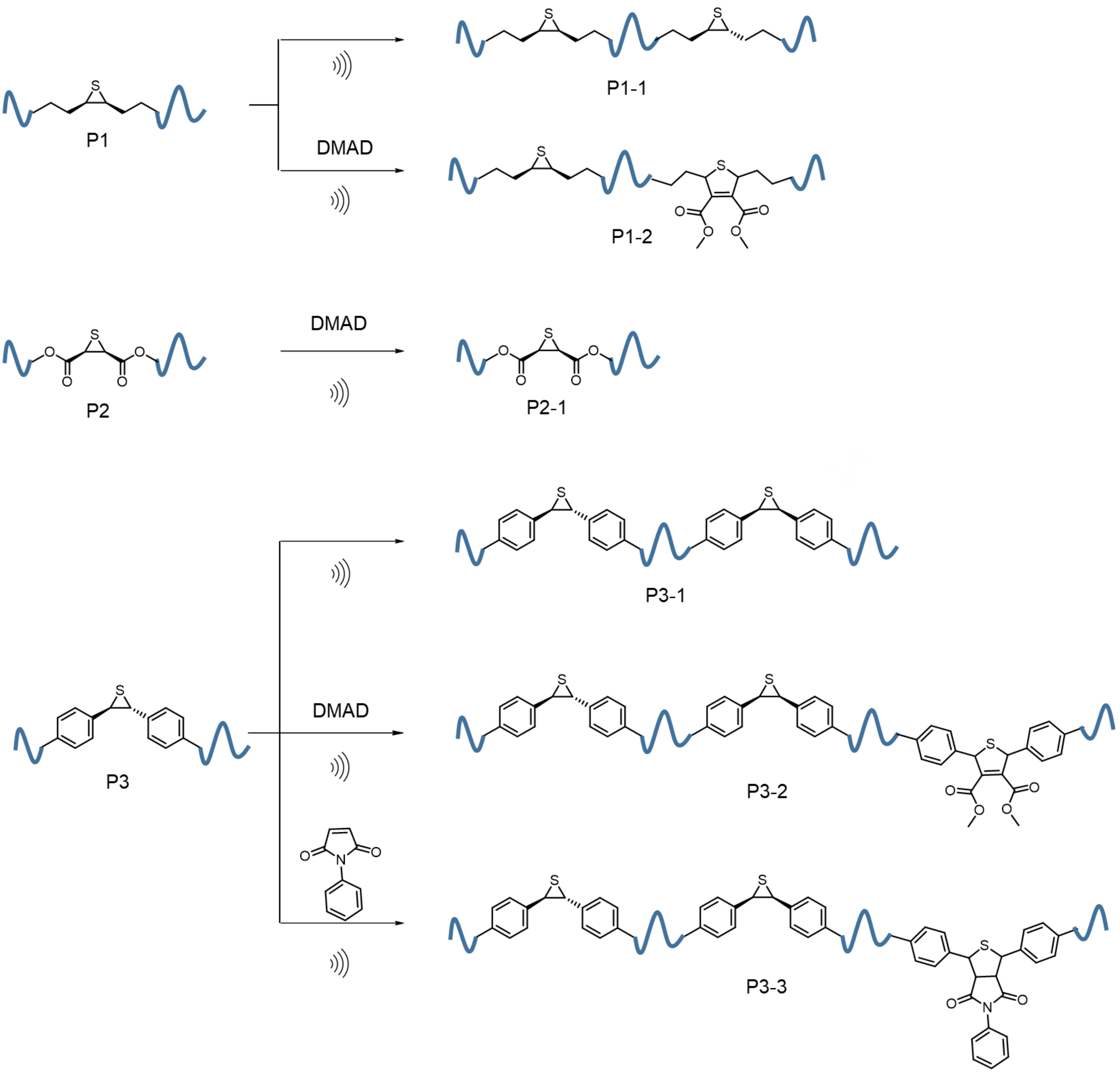

Figure S2. Episulfide-containing polymer sonication experiments done in this work.

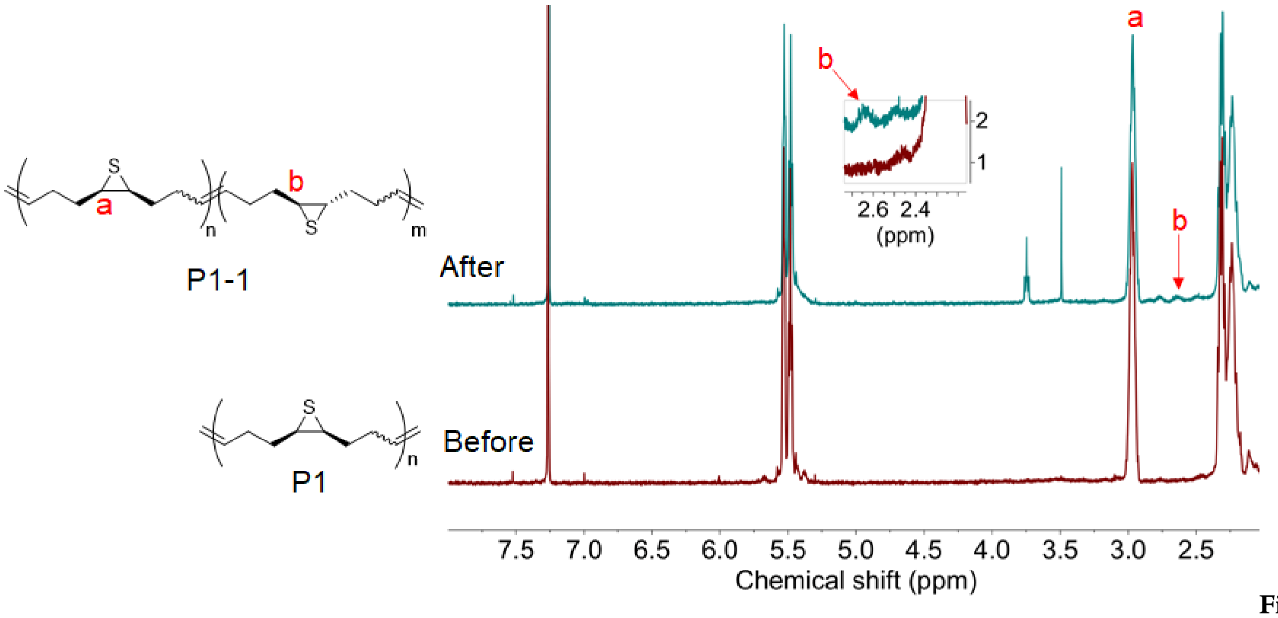

Figure S3. ^{1}H NMR spectrum of P1 before ultrasonication (bottom) and after sonication (top).

Control Experiment

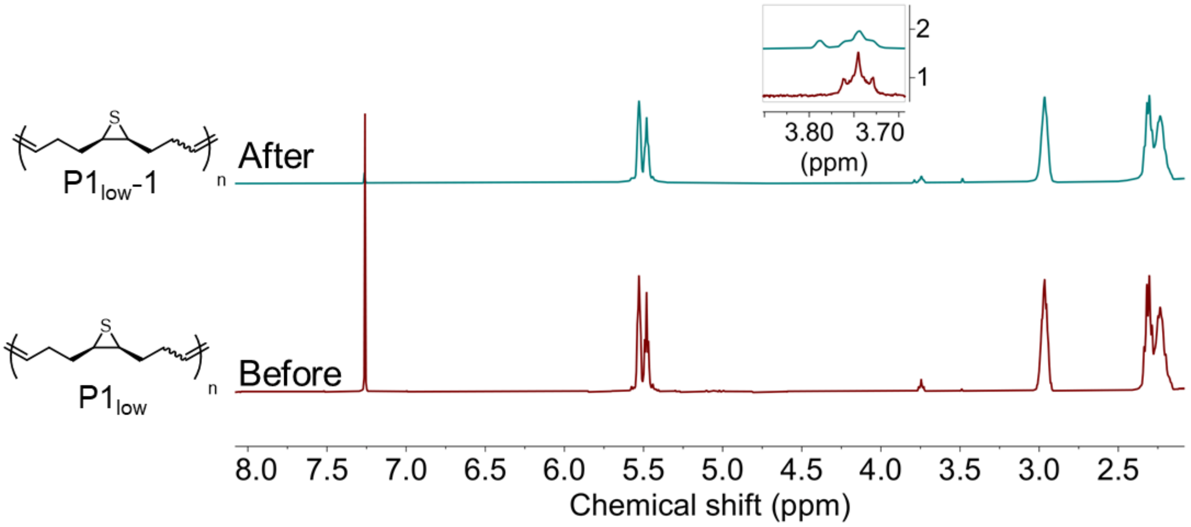

Figure S4. ^{1}H NMR spectrum of $P1_{low}$ (M_n: 16 kDa) before ultrasonication (bottom) and after ultrasonication (top).

Heating Experiment

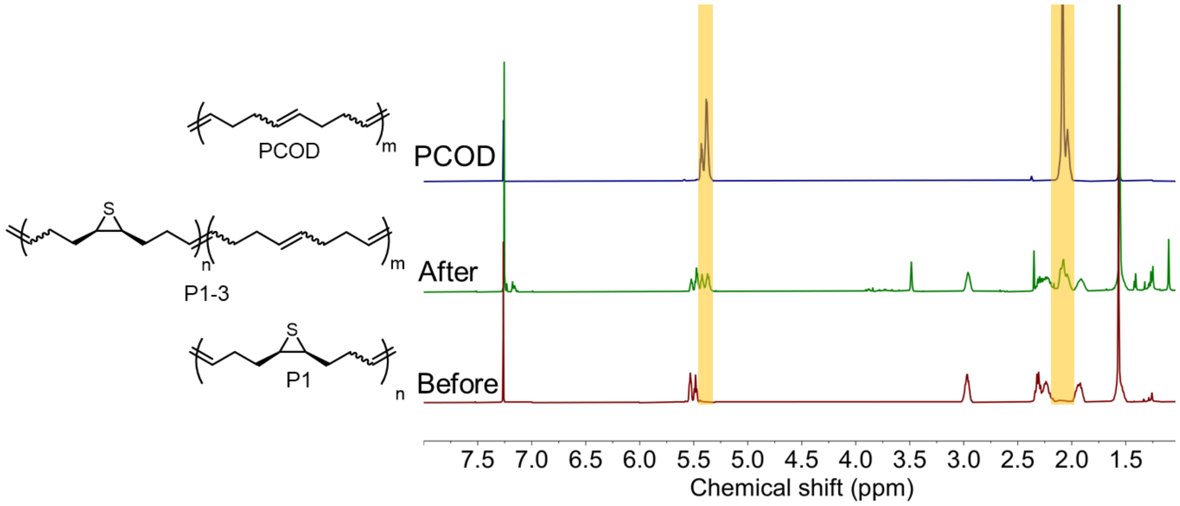

Figure S5. ^{1}H NMR spectrum of P1 (2 mg/ml) refluxed with DMAD (0.2M) in toluene for 24 h and pristine PCOD.

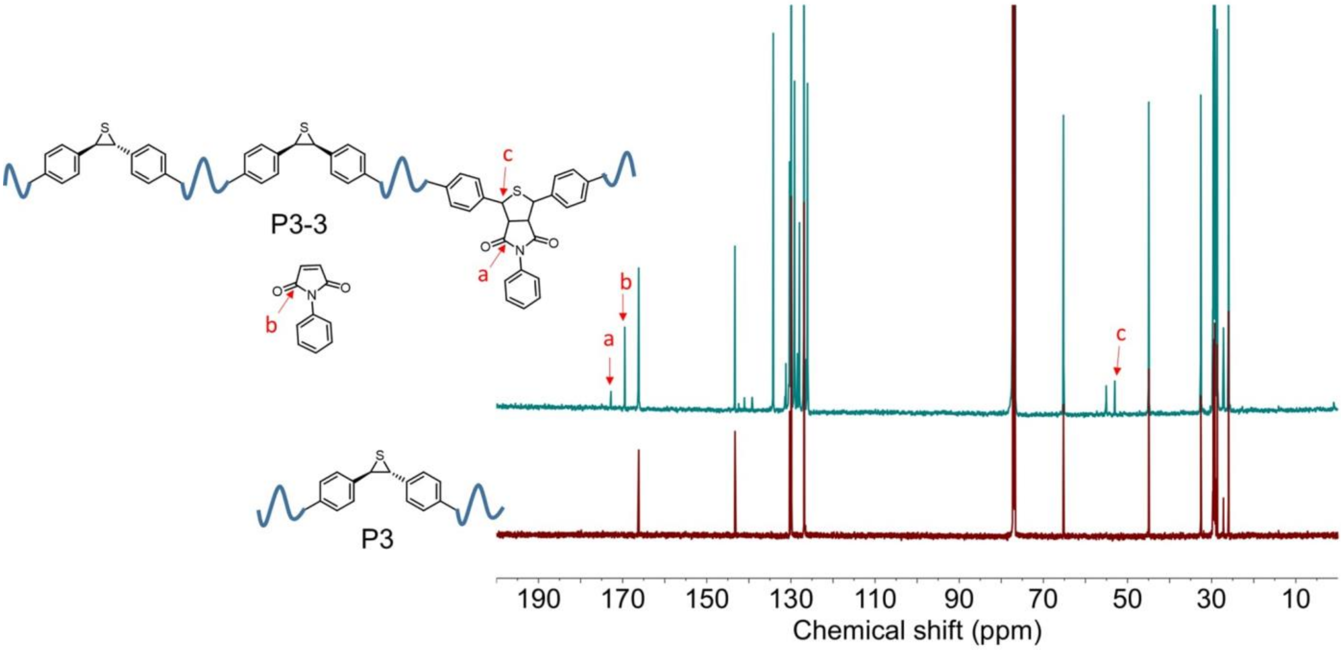

Figure S6. ^{13}C NMR spectrum of P3 with N-phenylmaleimide before ultrasonication (bottom) and after sonication (top).

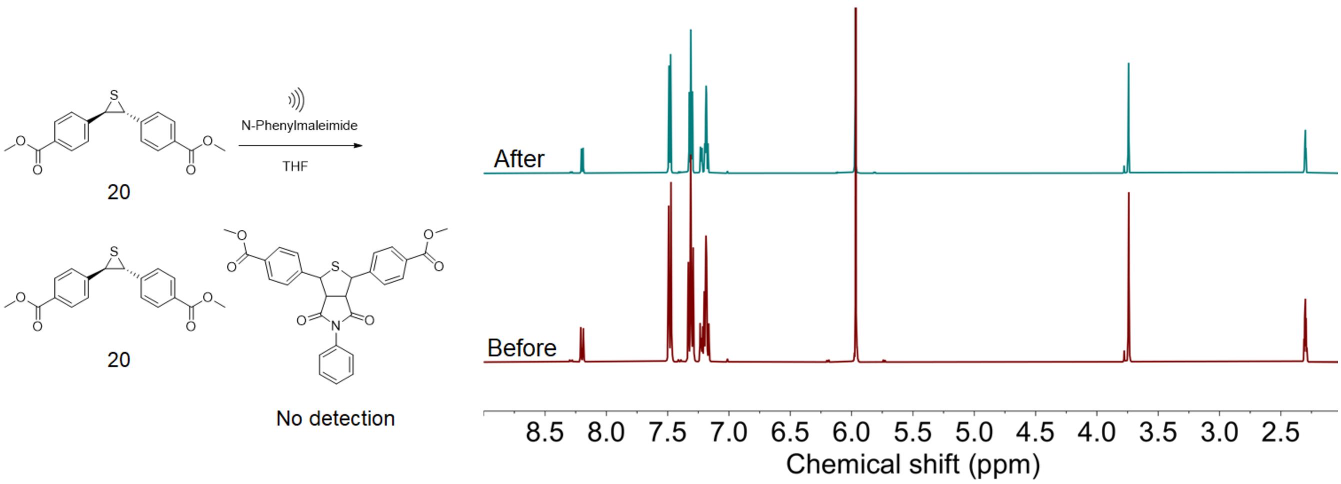

Figure S7. ^{1}H NMR spectrum of compound 20 and *N*-phenylmaleimide before ultrasonication (bottom) compared with after ultrasonication (top).

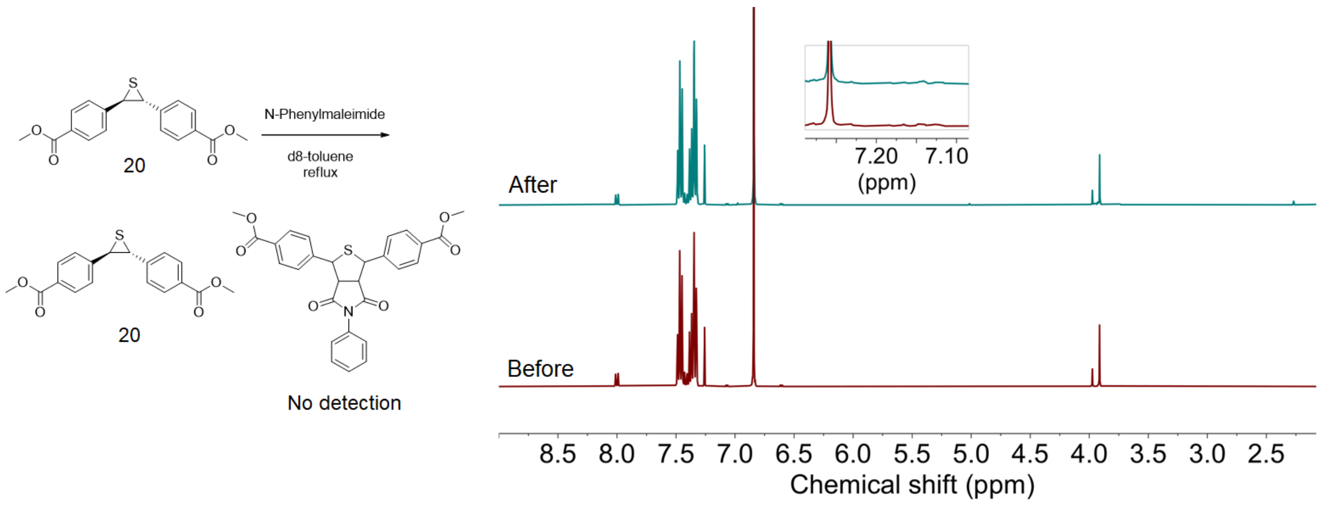

Figure S8. ^{1}H NMR spectrum of compound 20 (1 mg) reflux with *N*-phenylmaleimide (24 mg) in d_8-toluene (0.5 ml) for 24 h.

GPC Characterization of synthesized polymers

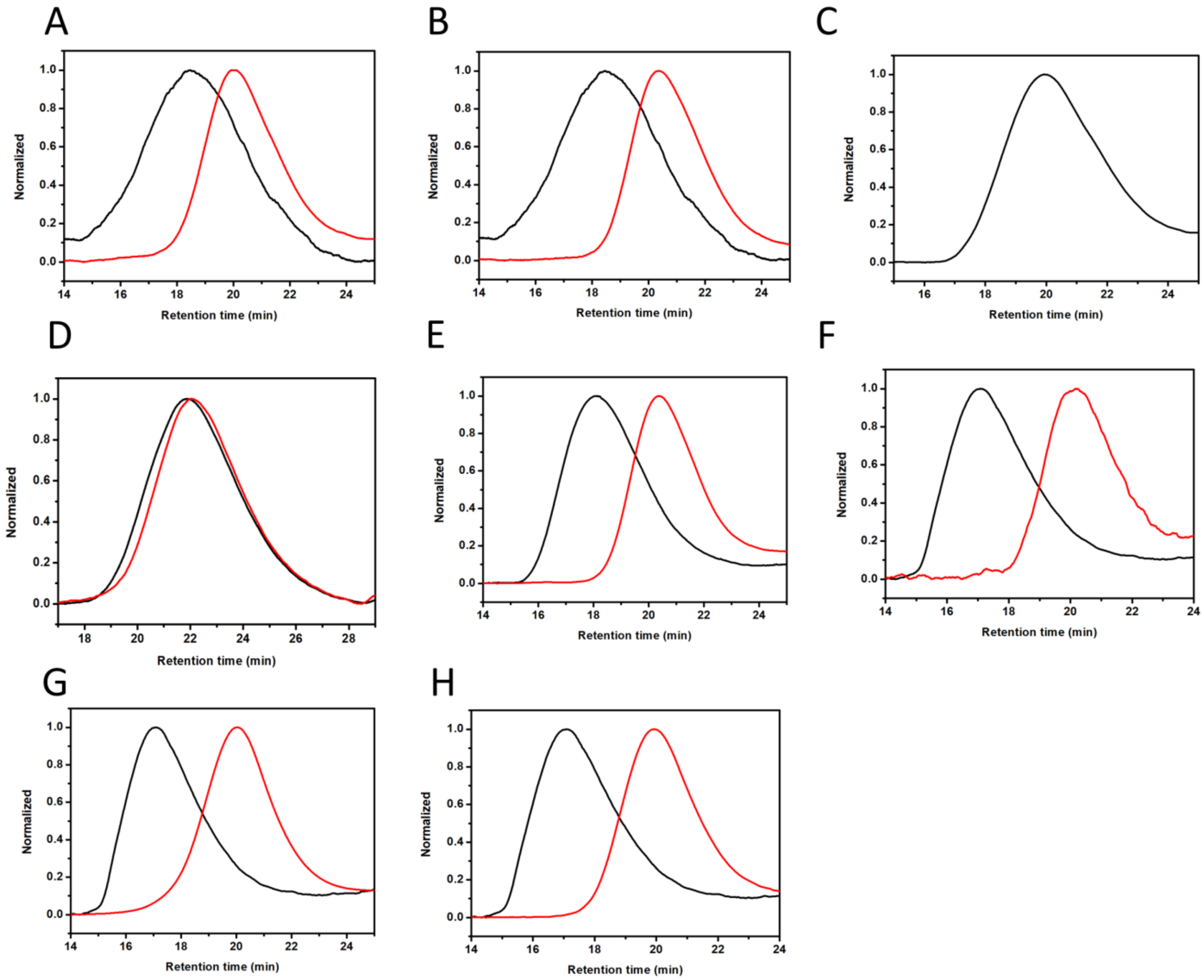

Figure S9. GPC traces of (A) P1 (black, M_n: 96 kDa, PDI: 1.78) and P1-1 (red, M_n: 46 kDa, PDI: 1.28). (B) P1 (black, M_n: 96 kDa, PDI: 1.78) and P1-2 (red, M_n: 57 kDa, PDI: 1.26). (C) PCOD (M_n: 54 kDa, PDI: 1.44). (D) $P1_{low}$ (black, M_n: 16 kDa, PDI: 1.75) and $P1_{low}$-1 (red, M_n: 15 kDa, PDI: 1.70). (E) P2 (black, M_n: 100 kDa, PDI: 1.85) and P2-1 (red, M_n: 58 kDa, PDI: 1.20). (F) P3 (black, M_n: 220 kDa, PDI: 2.0) and P3-1 (red, M_n: 59 kDa, PDI: 1.26). (G) P3 (black, M_n: 220 kDa, PDI: 2.0) and P3-2 (red, M_n: 69 kDa, PDI: 1.30). (H) P3 (black, M_n: 220 kDa, PDI: 2.0) and P3-3 (red, M_n: 64 kDa, PDI: 1.43).

Table S1. Molecular weight before and after sonication of polymers (2mg/mL) with 0.2M DMAD

	before	after	Scission Cycle
P1	96 kDa	57 kDa	0.75
P2	100 kDa	58 kDa	0.79
P3	220 kDa	69 kDa	1.67

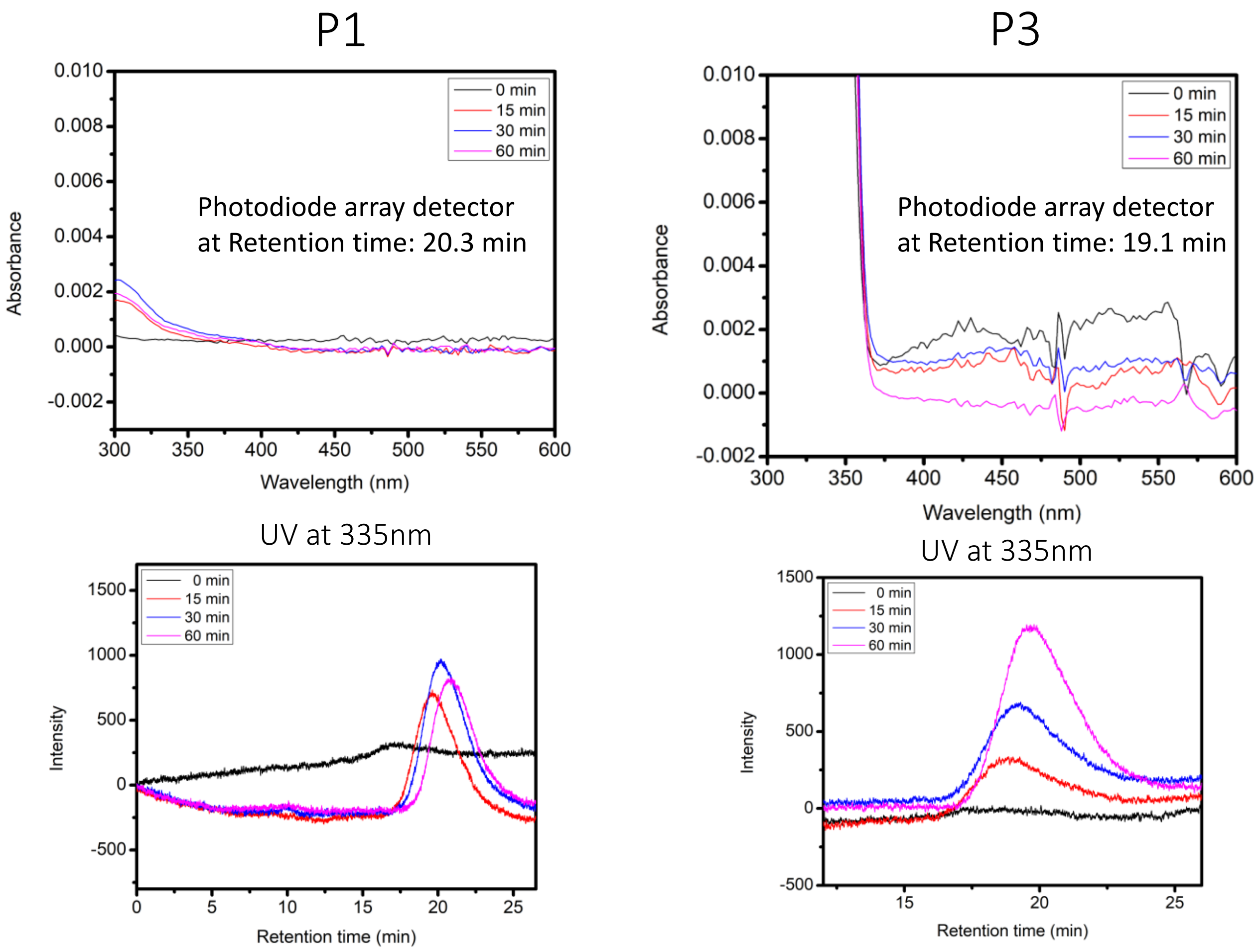

Figure S10. Sonication experiments in the presence of radical scavenger coumarin–2,2,6,6-tetramethylpiperidine-1-oxyl; P1 (Mn: 167 kDa, Đ: 2.17); P3 (Mn: 108 kDa, , Đ: 1.96), P1 and P3 were sonicated for 120 min in 32 mM CT solutions in THF at 25% amplitude, the method was adapted from reference 9.

^{1}H and ^{13}C NMR spectrum

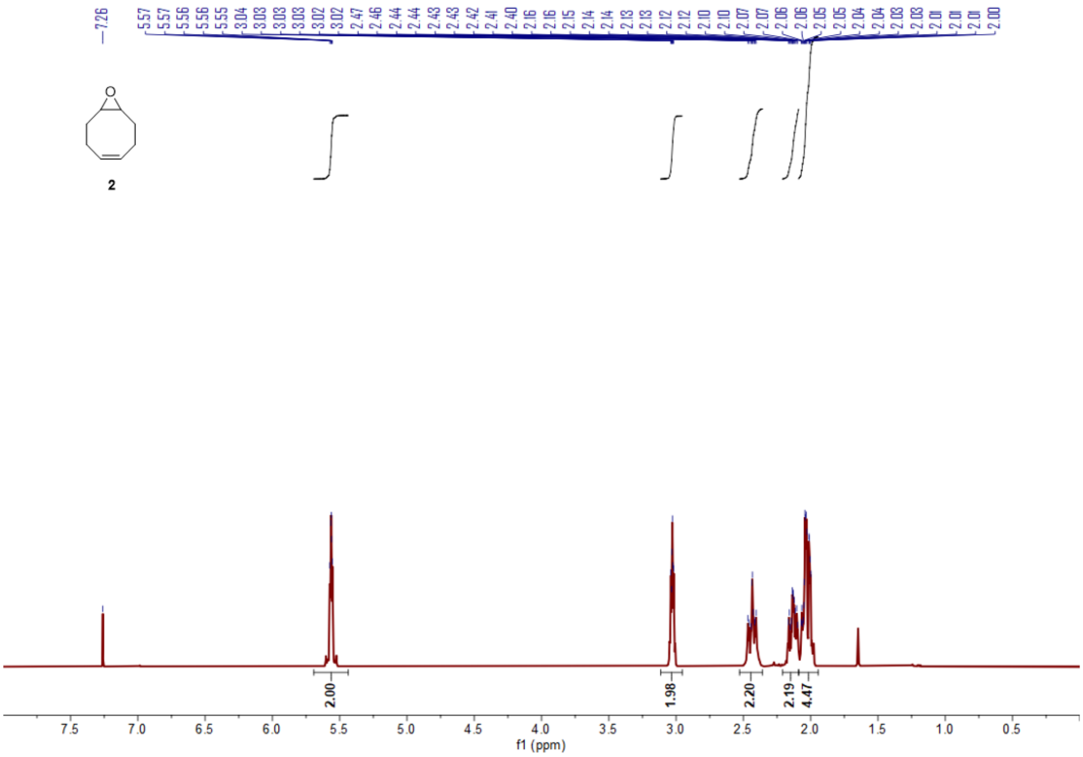

Figure S11. ^{1}H NMR (400 *MHz*, $CDCl_3$) spectrum of **Compound 2**.

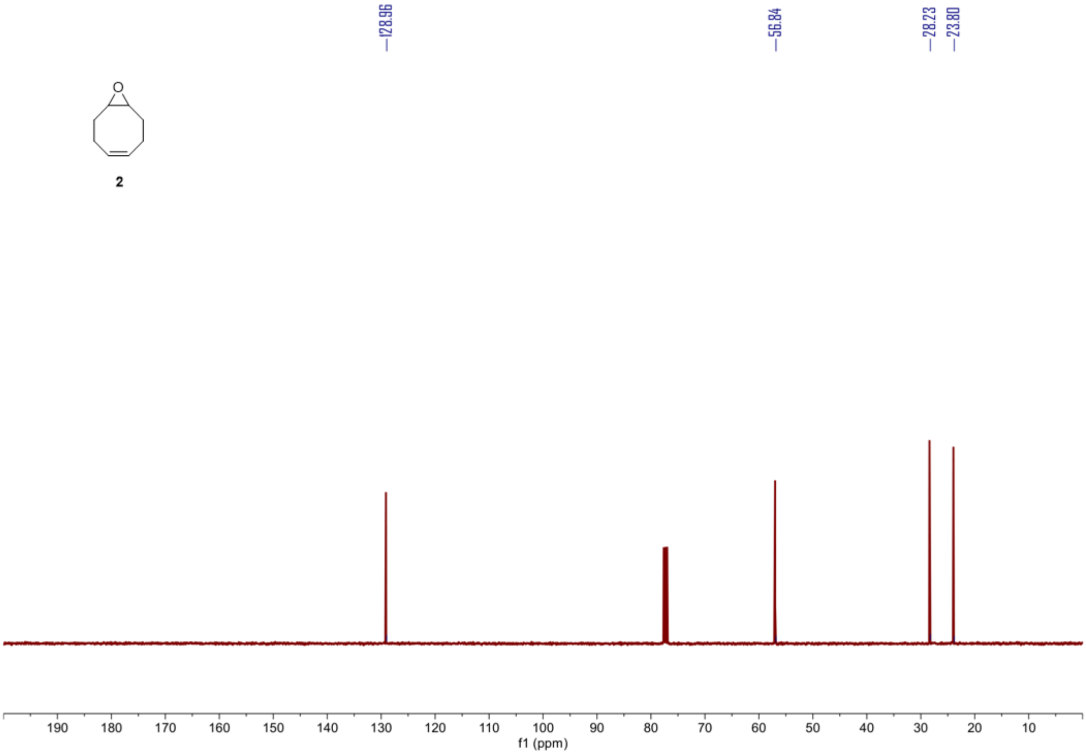

Figure S12. ^{13}C NMR (100 *MHz*, $CDCl_3$) spectrum of **Compound 2**.

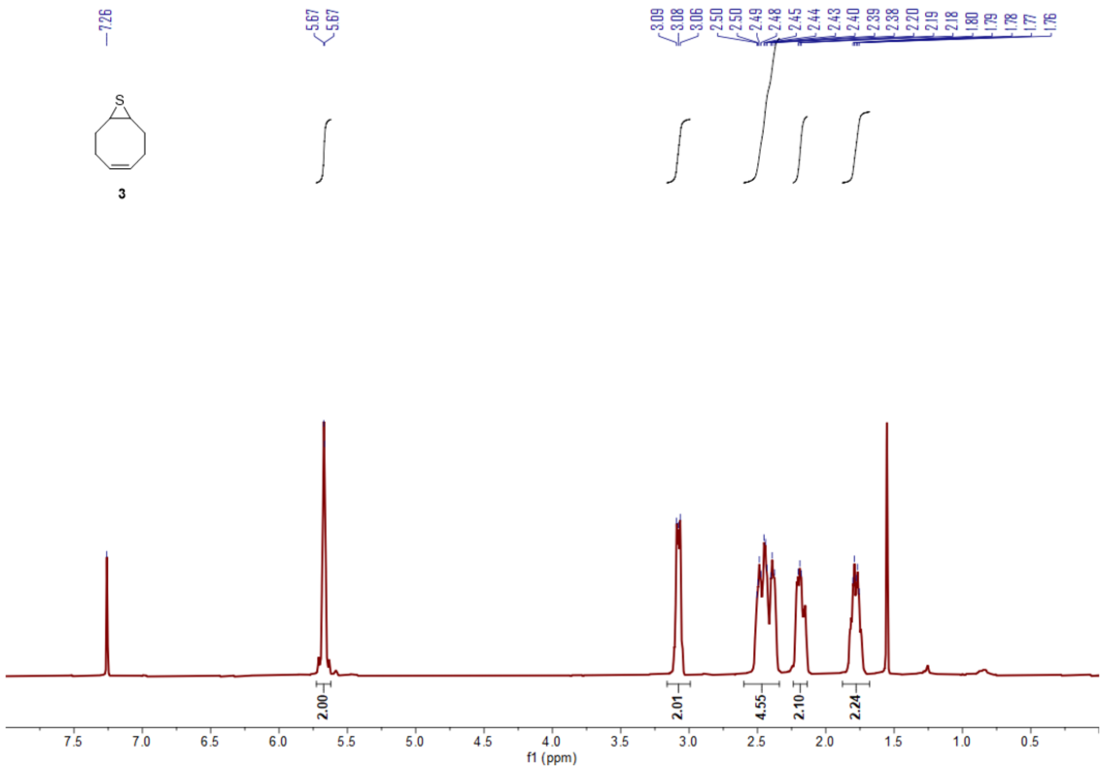

Figure S13. ^{1}H NMR (400 *MHz*, $CDCl_3$) spectrum of **Compound 3**.

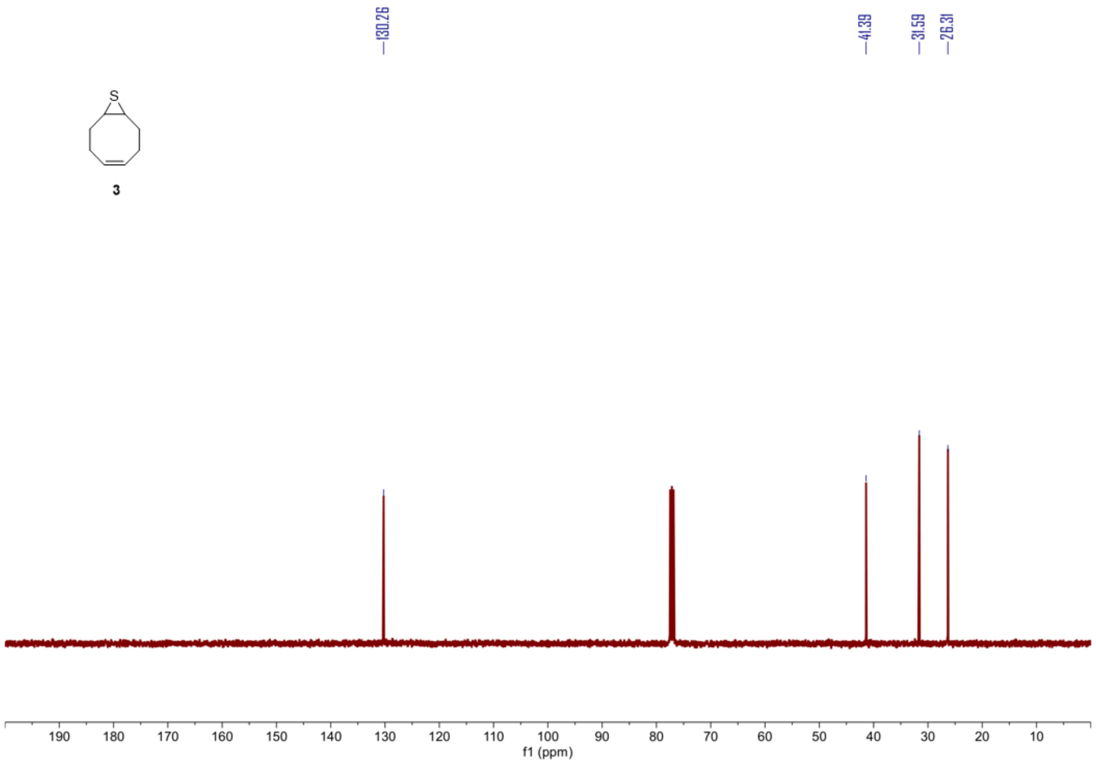

Figure S14. ^{13}C NMR (100 *MHz*, $CDCl_3$) spectrum of **Compound 3**.

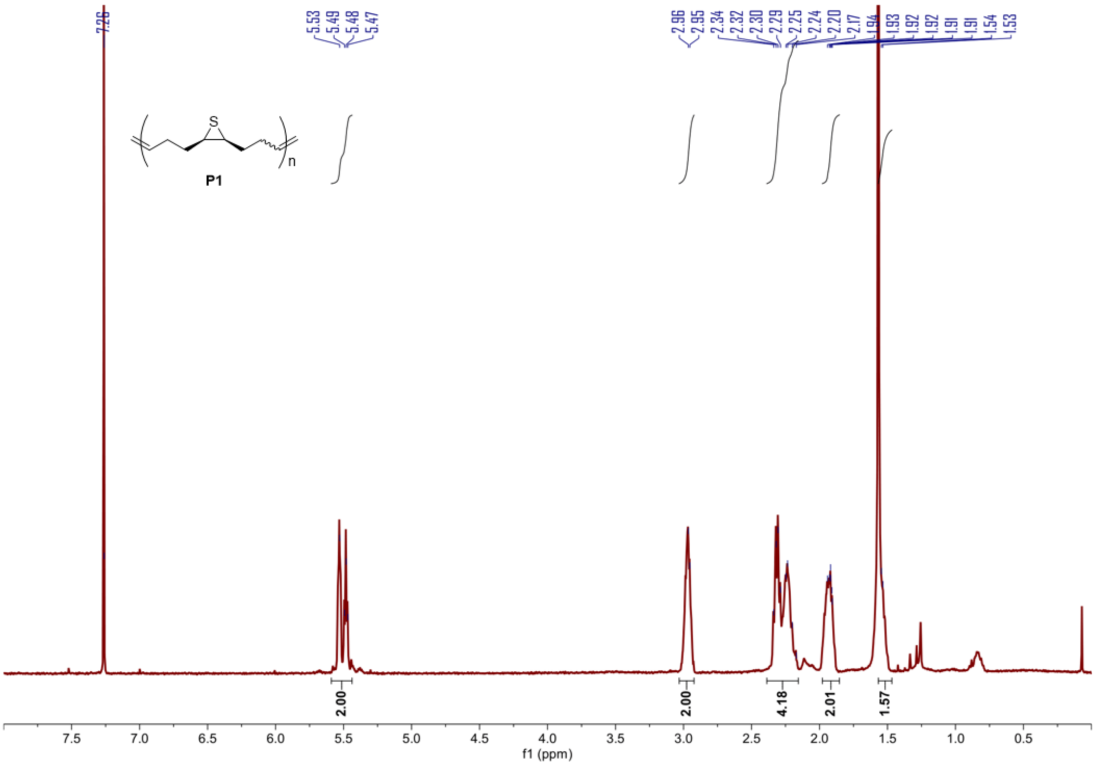

Figure S15. ^{1}H NMR (400 MHz, $CDCl_3$) spectrum of **P1**.

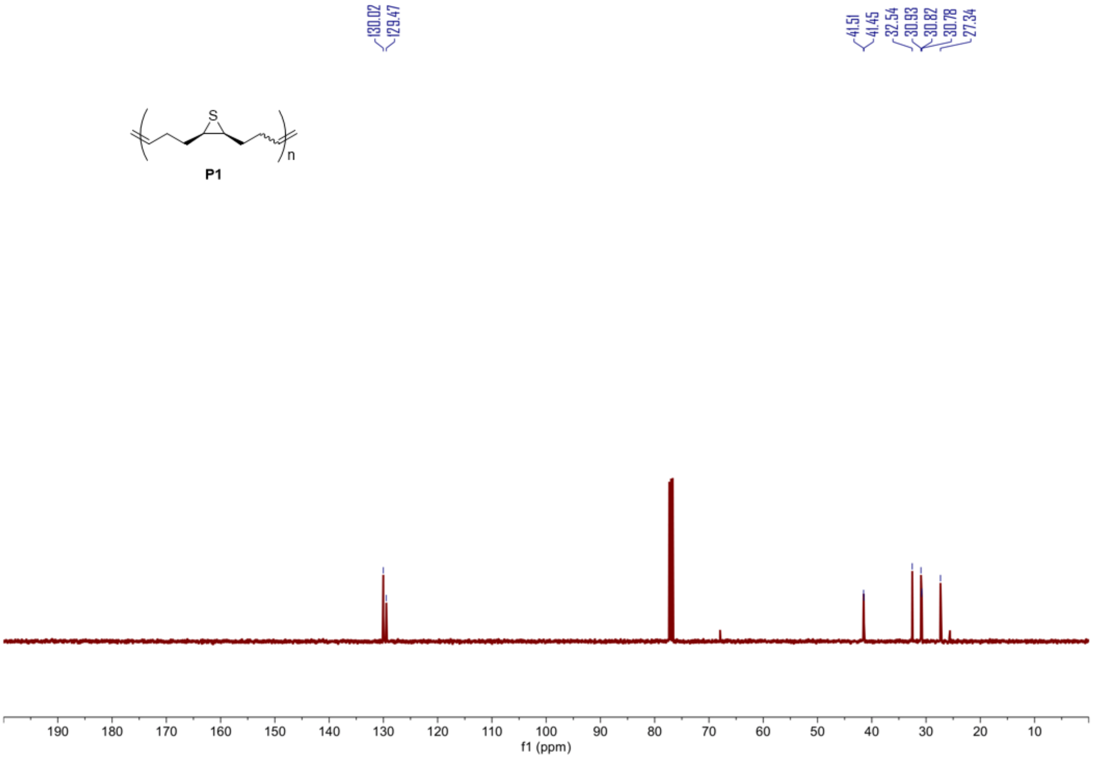

Figure S16. ^{13}C NMR (100 MHz, $CDCl_3$) spectrum of **P1**.

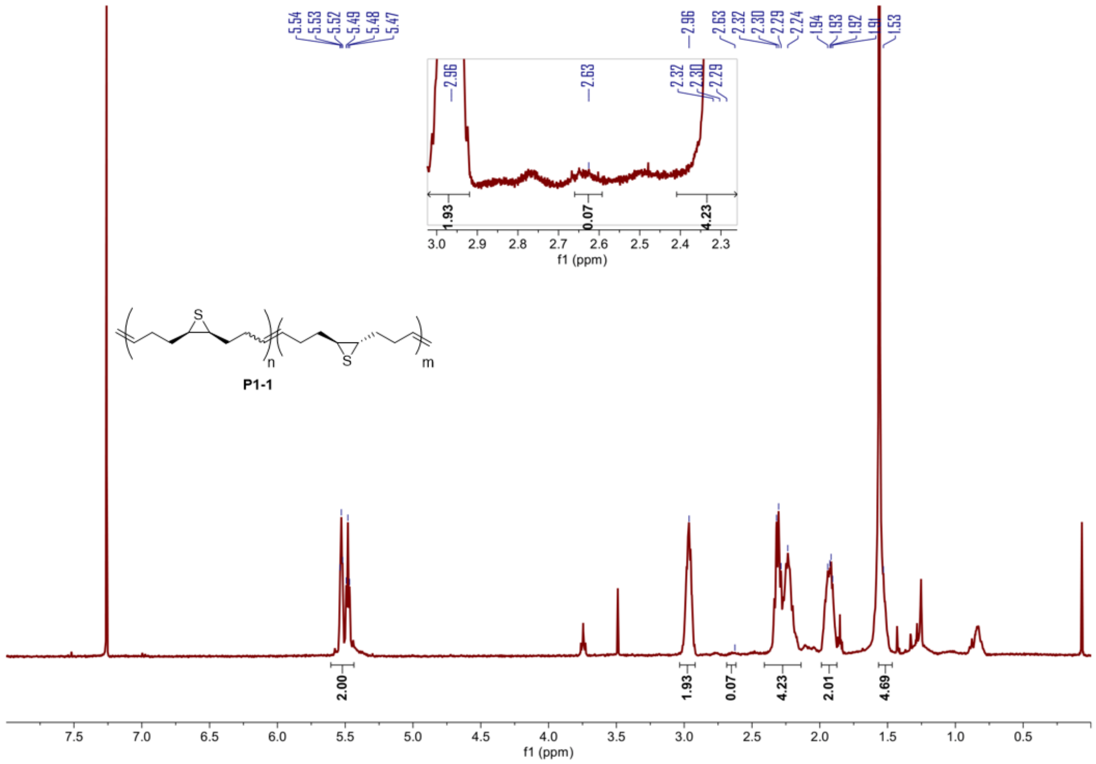

Figure S17. ^{1}H NMR (400 *MHz*, $CDCl_3$) spectrum of **P1-1**. The residual solvent peaks were MeOH at 3.5 ppm and THF at 3.7ppm.

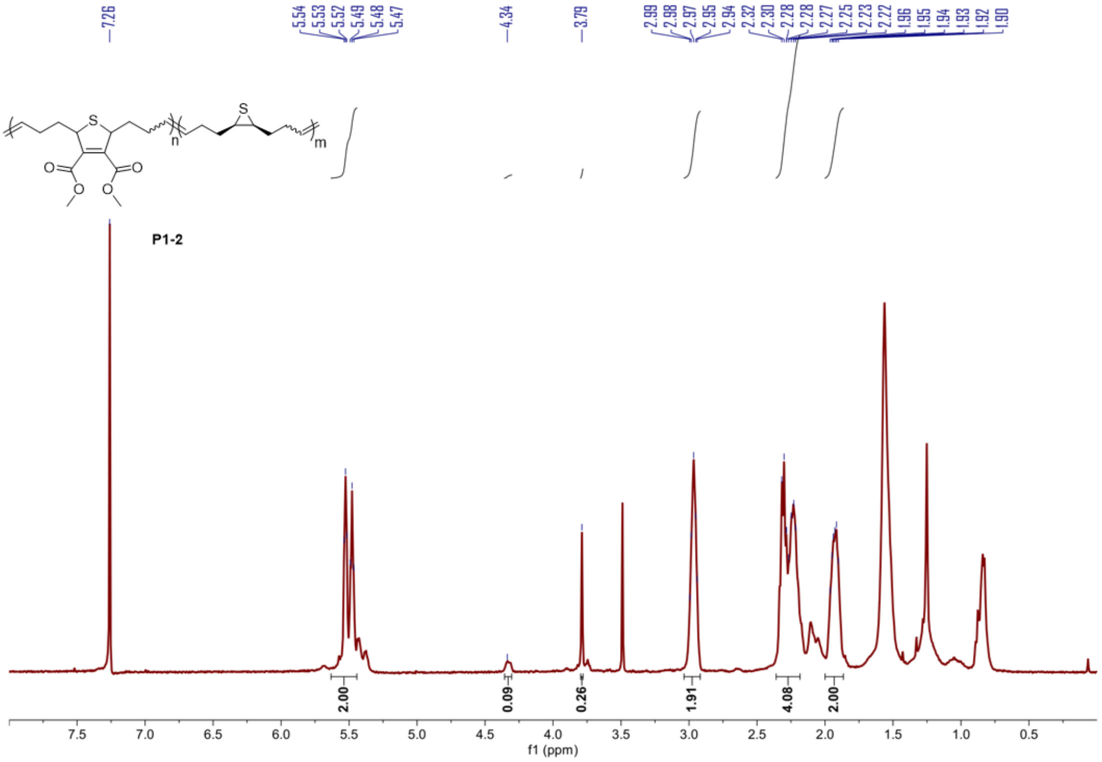

Figure S18. ^{1}H NMR (400 *MHz*, $CDCl_3$) spectrum of **P1-2**. The residual solvent peaks were MeOH at 3.5 ppm and THF at 3.7ppm.

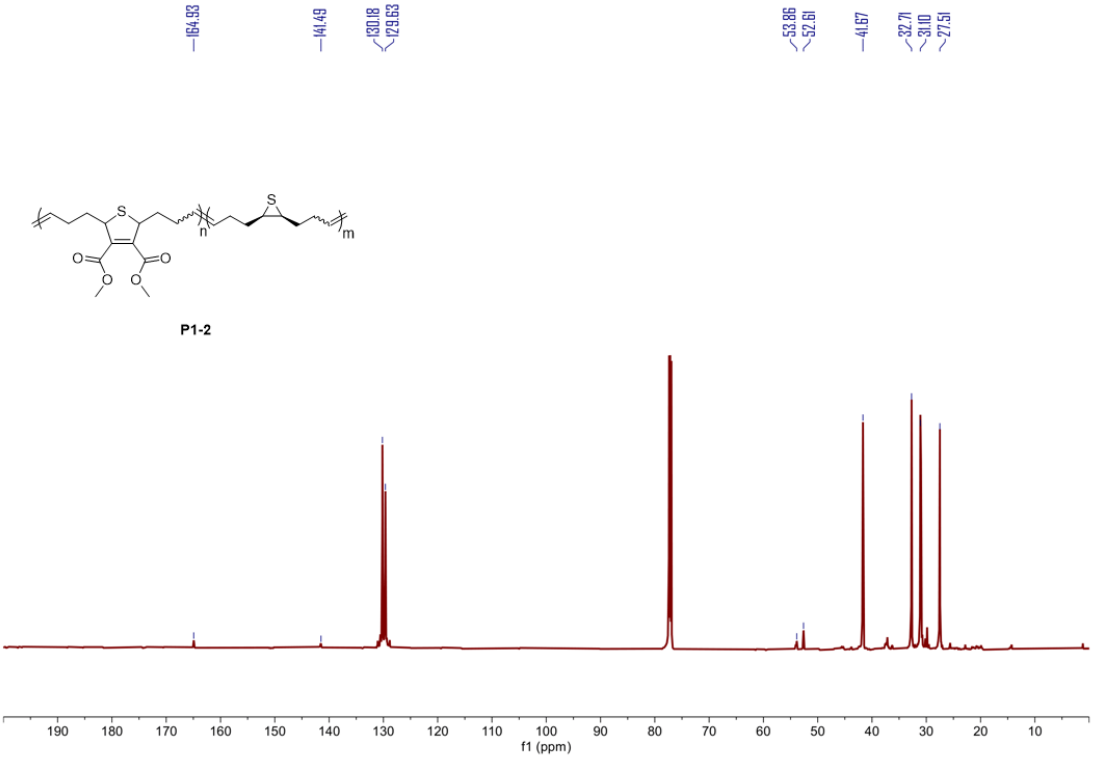

Figure S19. ^{13}C NMR (100 *MHz*, $CDCl_3$) spectrum of **P1-2**.

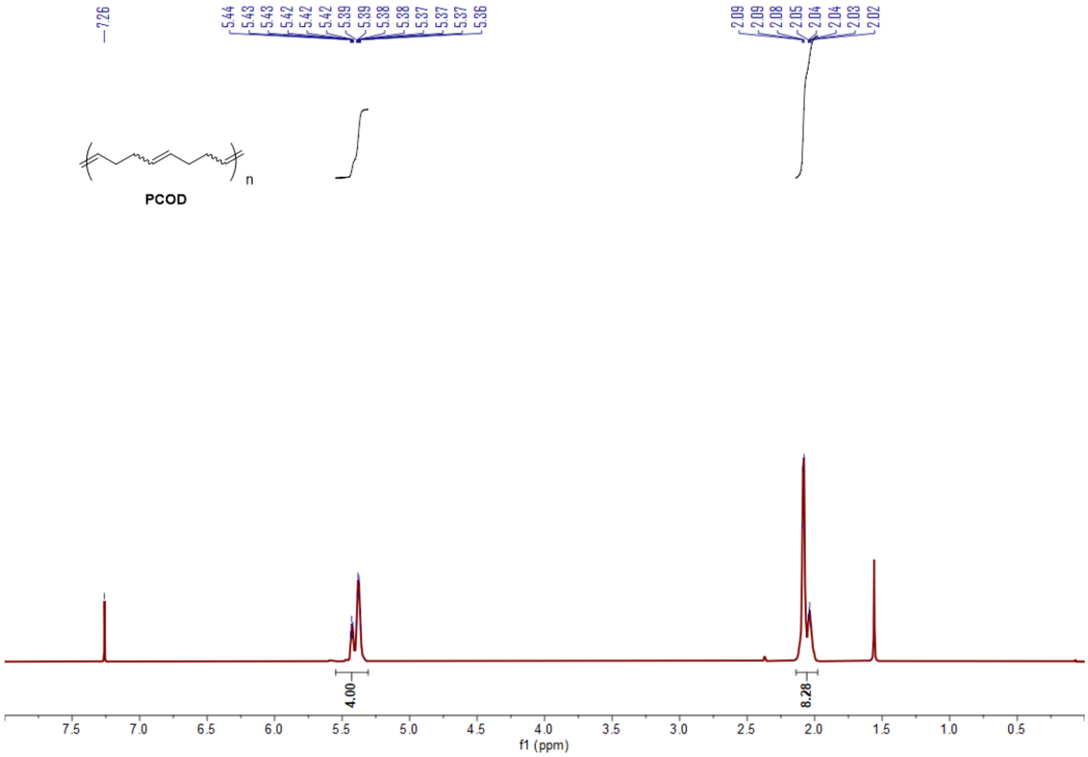

Figure S20. ^{1}H NMR (400 *MHz*, $CDCl_3$) spectrum of **PCOD**.

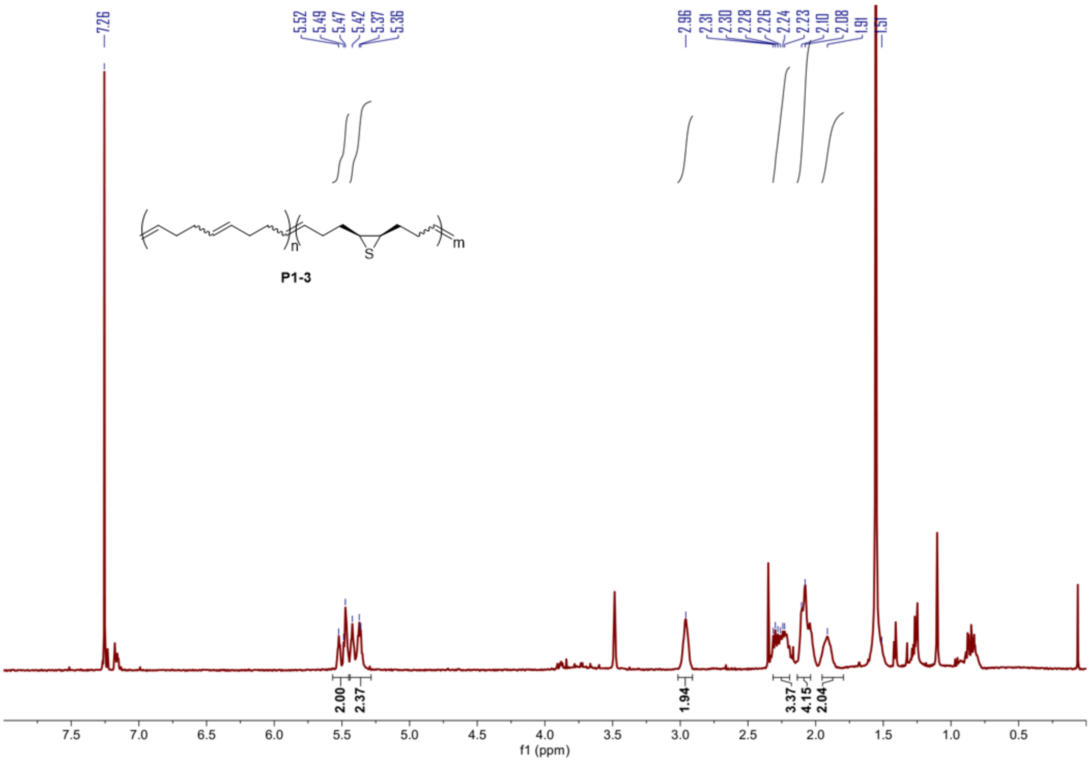

Figure S21. ^{1}H NMR (400 *MHz*, $CDCl_3$) spectrum of **P1-3**. The residual solvent peaks were MeOH at 3.5 ppm.

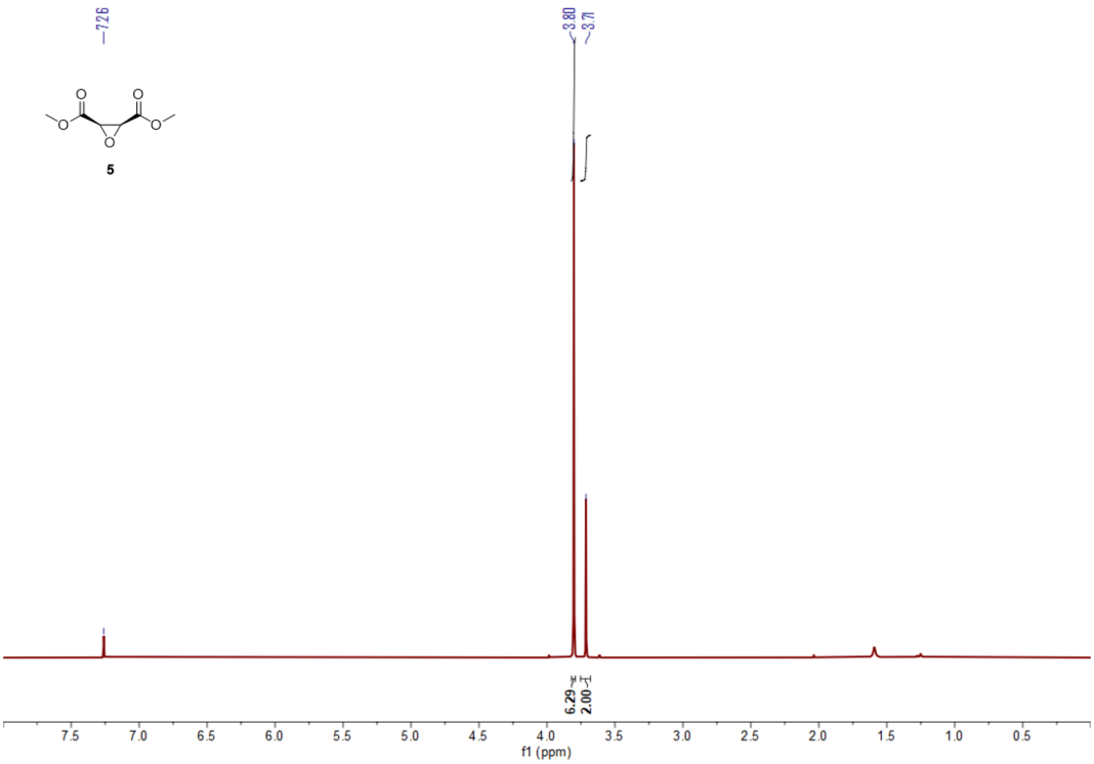

Figure S22. ^{1}H NMR (400 *MHz*, $CDCl_3$) spectrum of **Compound 5**.

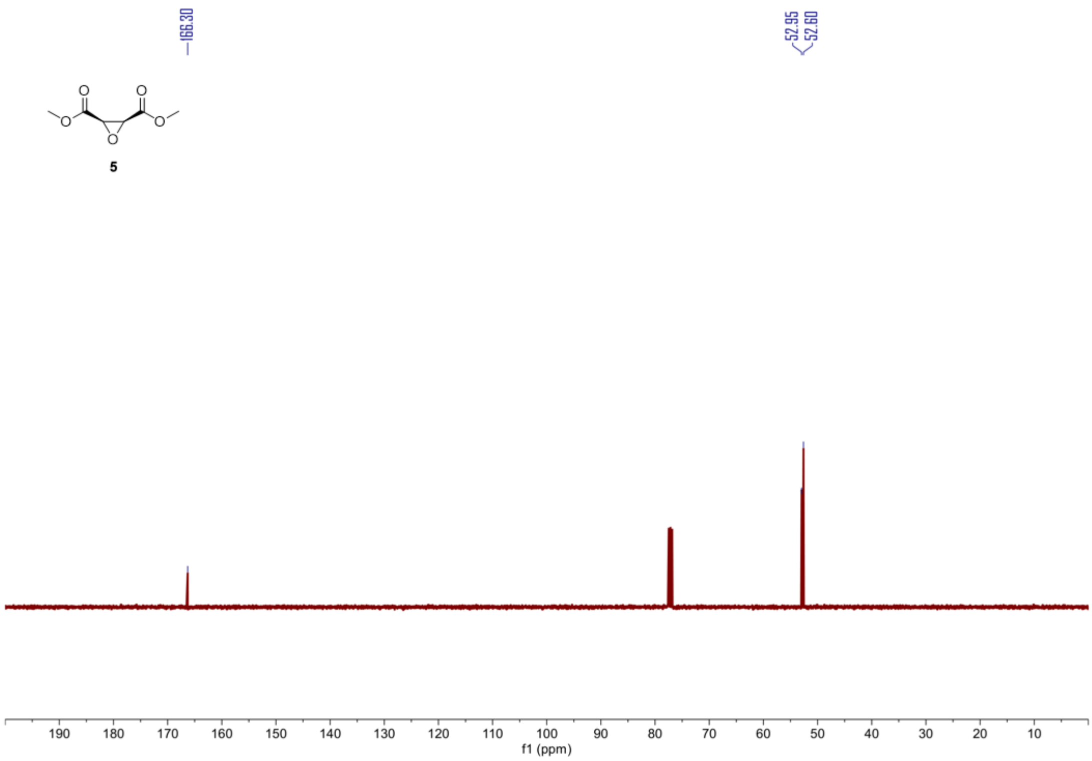

Figure S23. ^{13}C NMR (100 *MHz*, $CDCl_3$) spectrum of **Compound 5**.

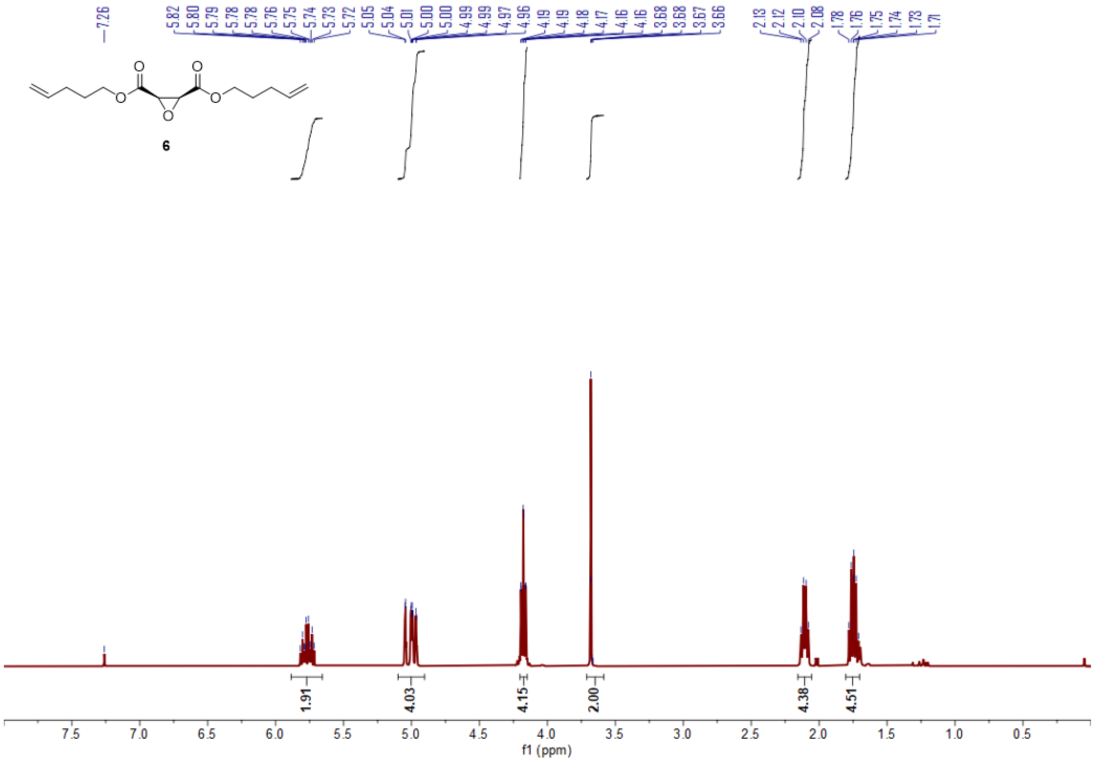

Figure S24. ^{1}H NMR (400 *MHz*, $CDCl_3$) spectrum of **Compound 6**.

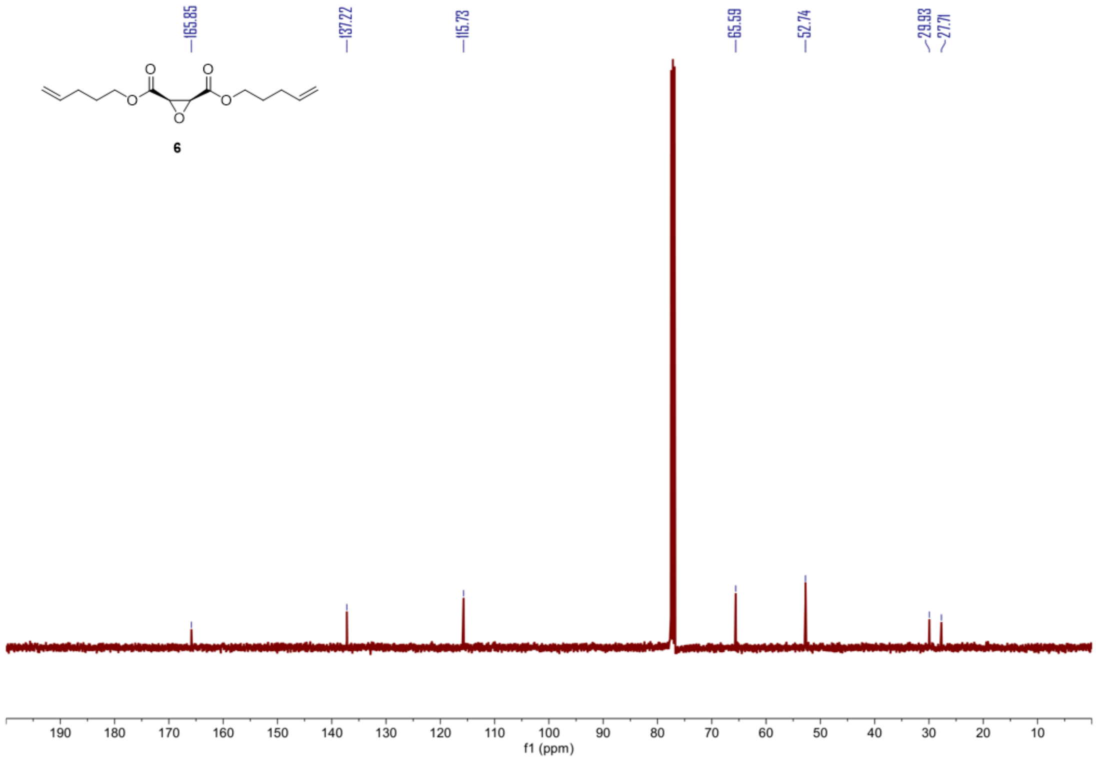

Figure S25. ^{13}C NMR (100 *MHz,* $CDCl_3$) spectrum of **Compound 6**.

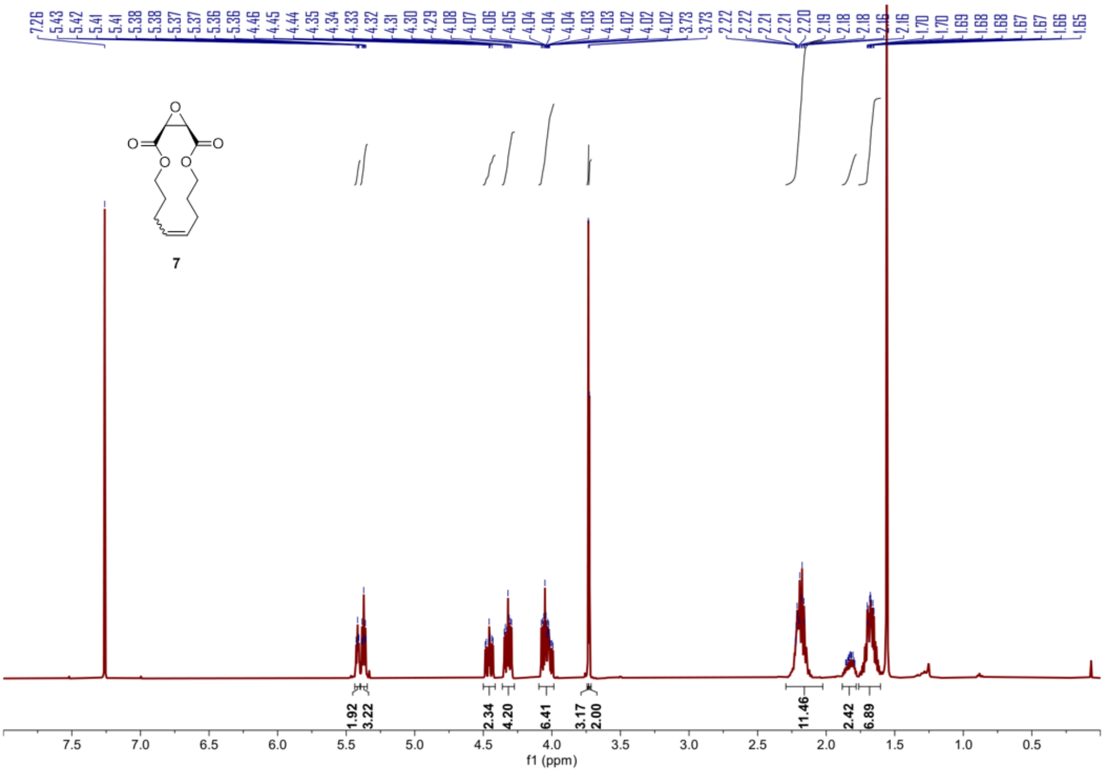

Figure S26. ^{1}H NMR (400 MHz, $CDCl_3$) spectrum of **Compound 7**.

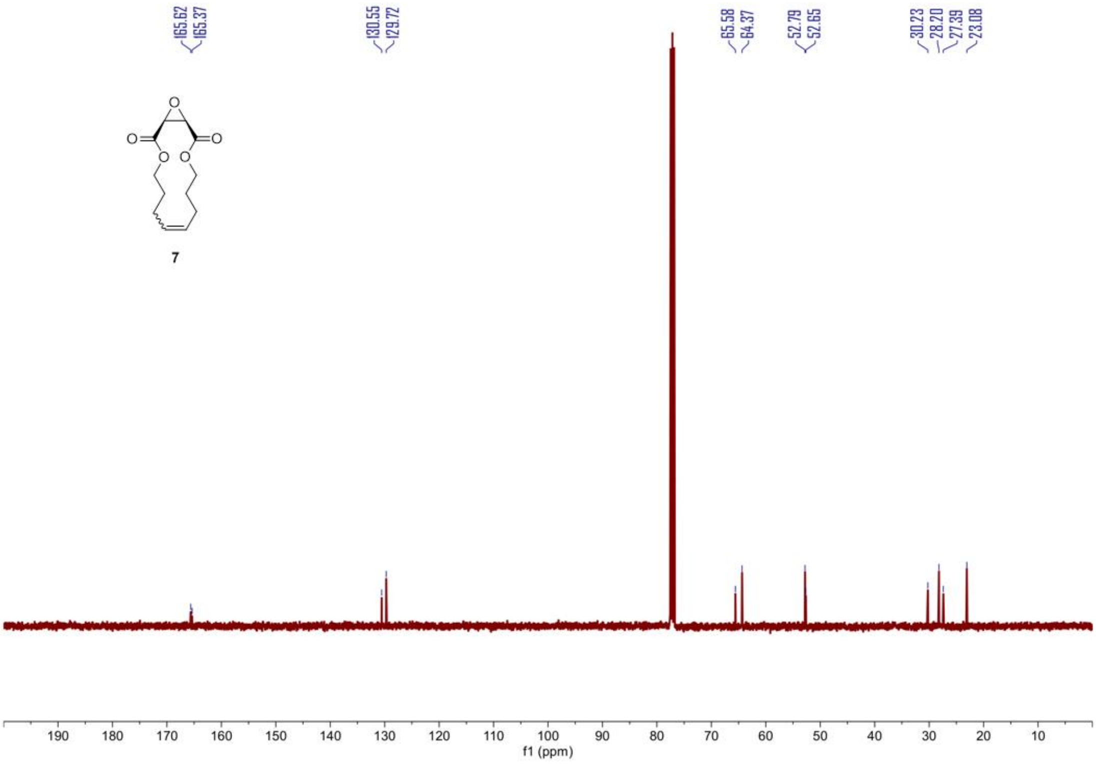

Figure S27. ^{13}C NMR (100 *MHz,* $CDCl_3$) spectrum of **Compound 7**.

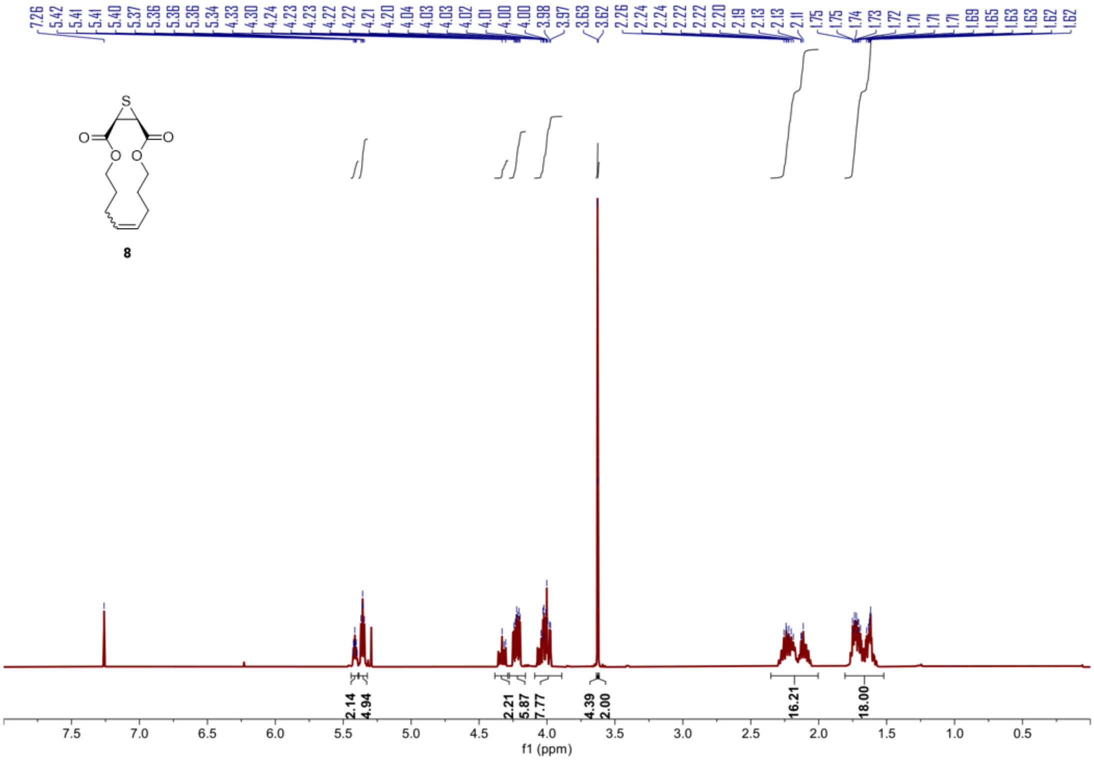

Figure S28. ^{1}H NMR (400 *MHz,* $CDCl_3$) spectrum of **Compound 8**.

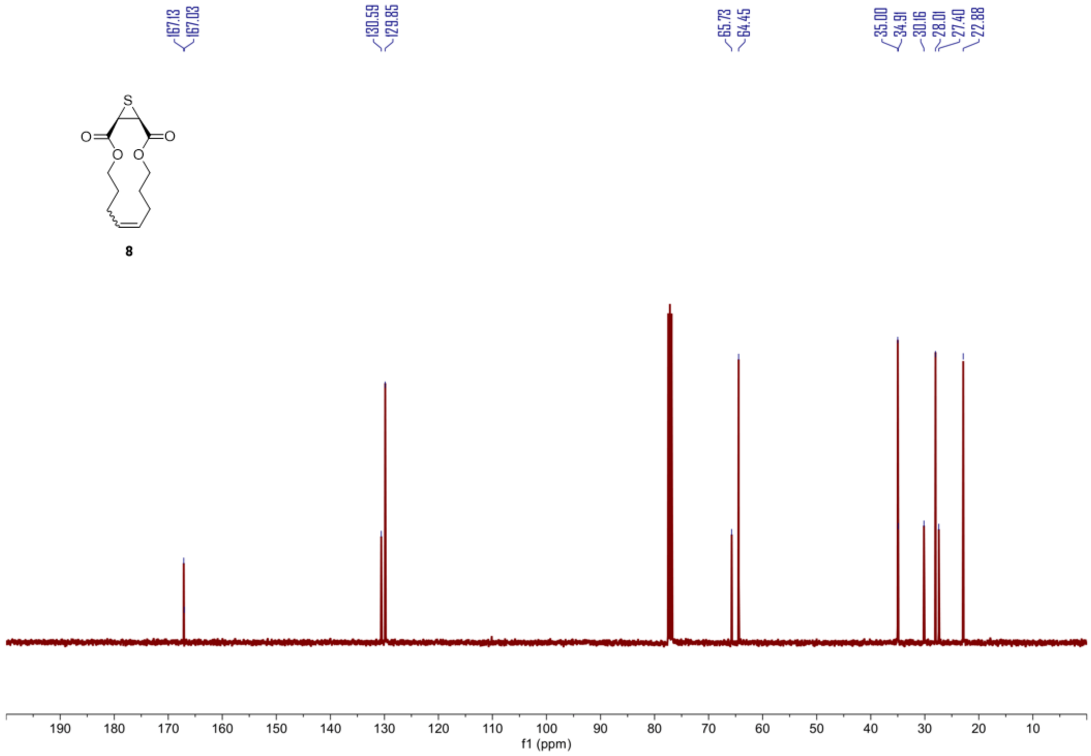

Figure S29. ^{13}C NMR (100 MHz, $CDCl_3$) spectrum of **Compound 8**.

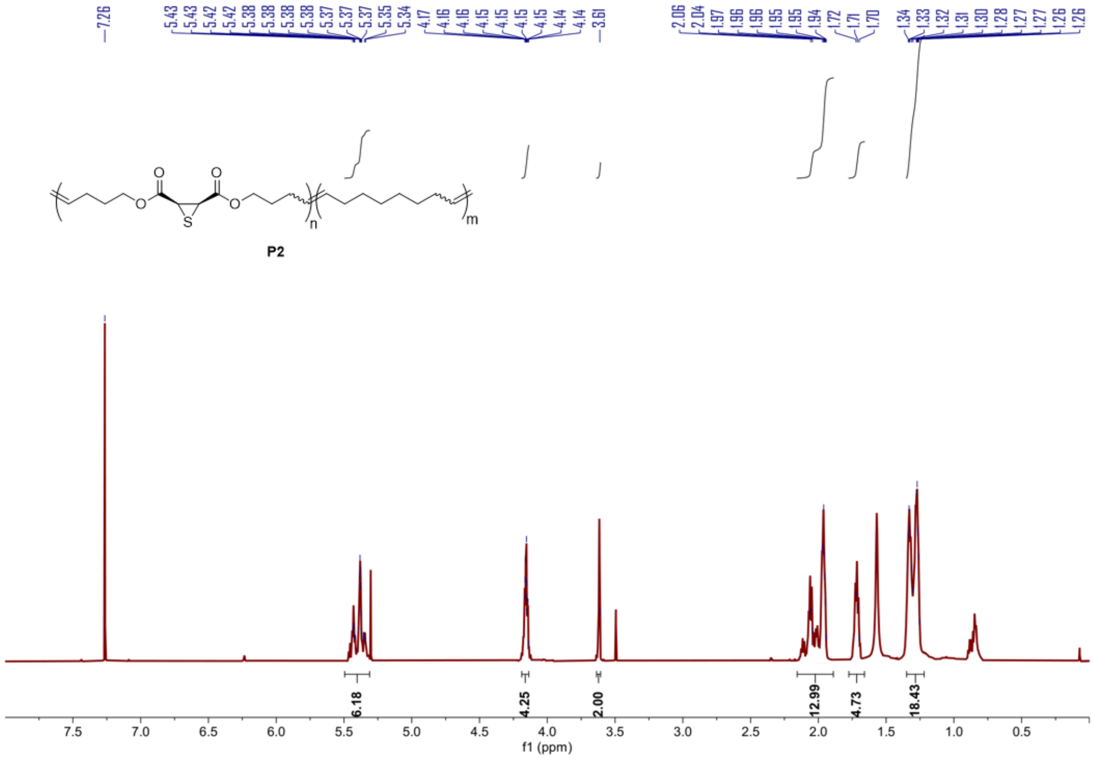

Figure S30. ^{1}H NMR (600 *MHz*, $CDCl_3$) spectrum of **P2**. The residual solvent peaks were MeOH at 3.5 ppm and DCM at 5.3 ppm.

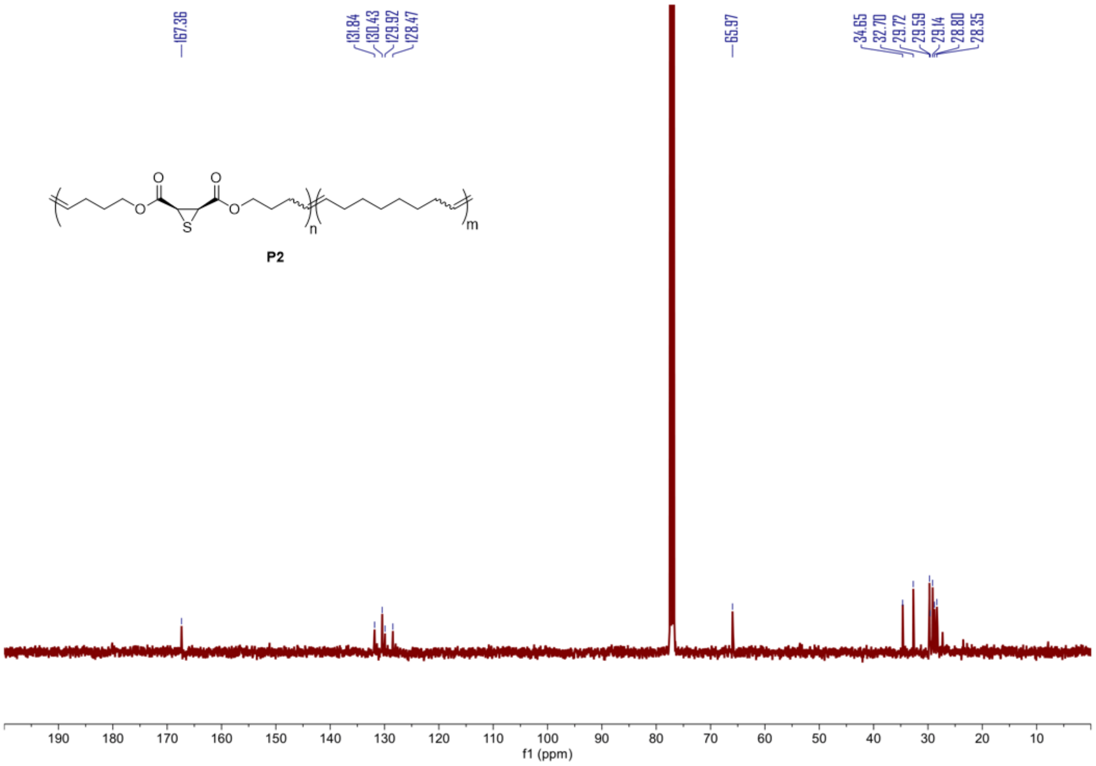

Figure S31. ^{13}C NMR (100 *MHz*, $CDCl_3$) spectrum of **P2**.

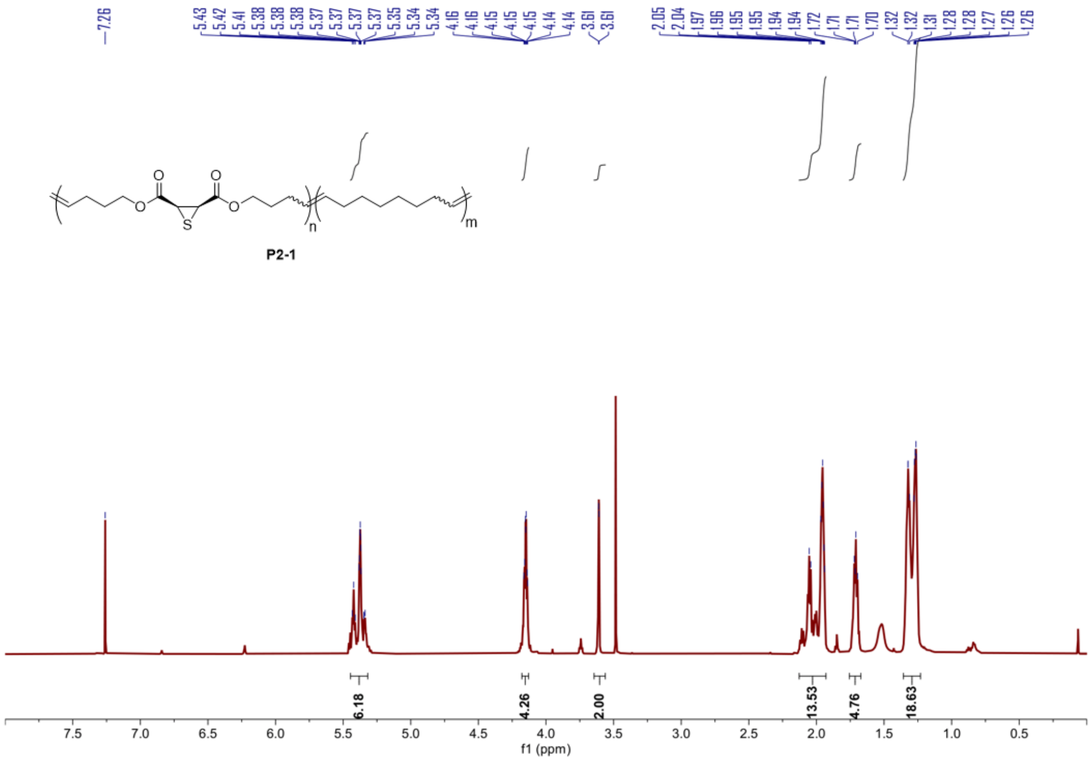

Figure S32. ^{1}H NMR (600 *MHz*, $CDCl_3$) spectrum of **P2-1**. The residual solvent peaks were MeOH at 3.5 ppm and THF at 3.7ppm.

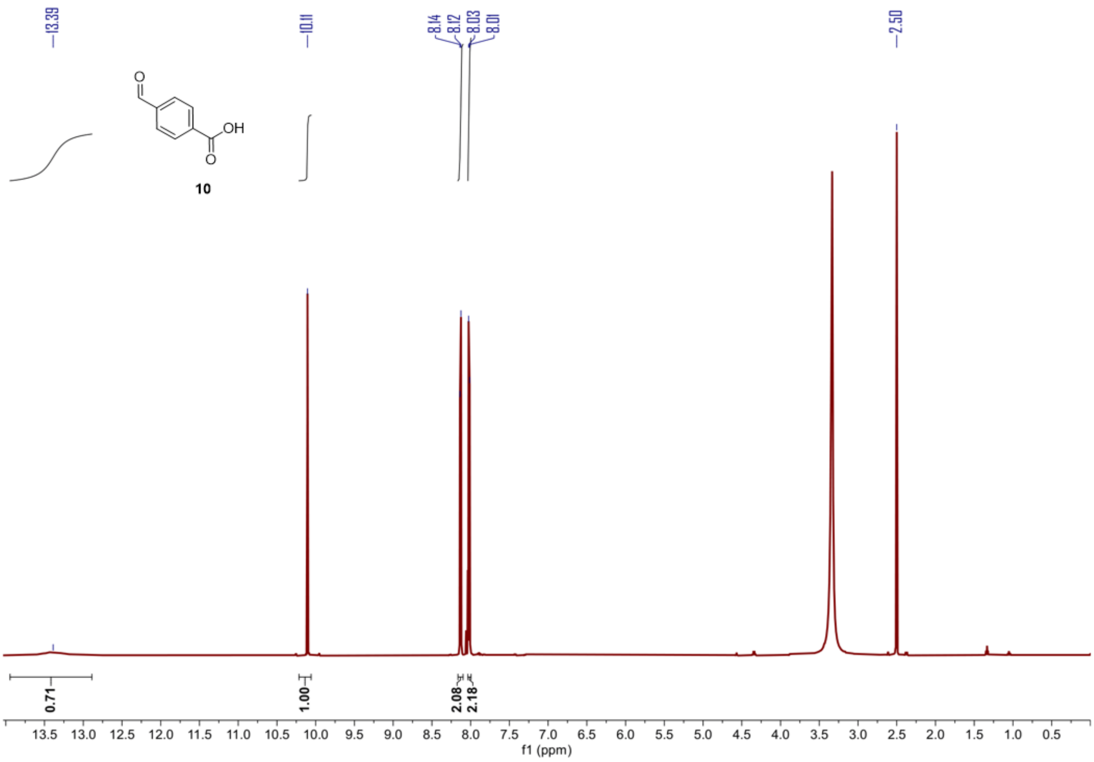

Figure S33. ^{1}H NMR (600 *MHz*, DMSO-*d₆*) spectrum of **Compound 10**.

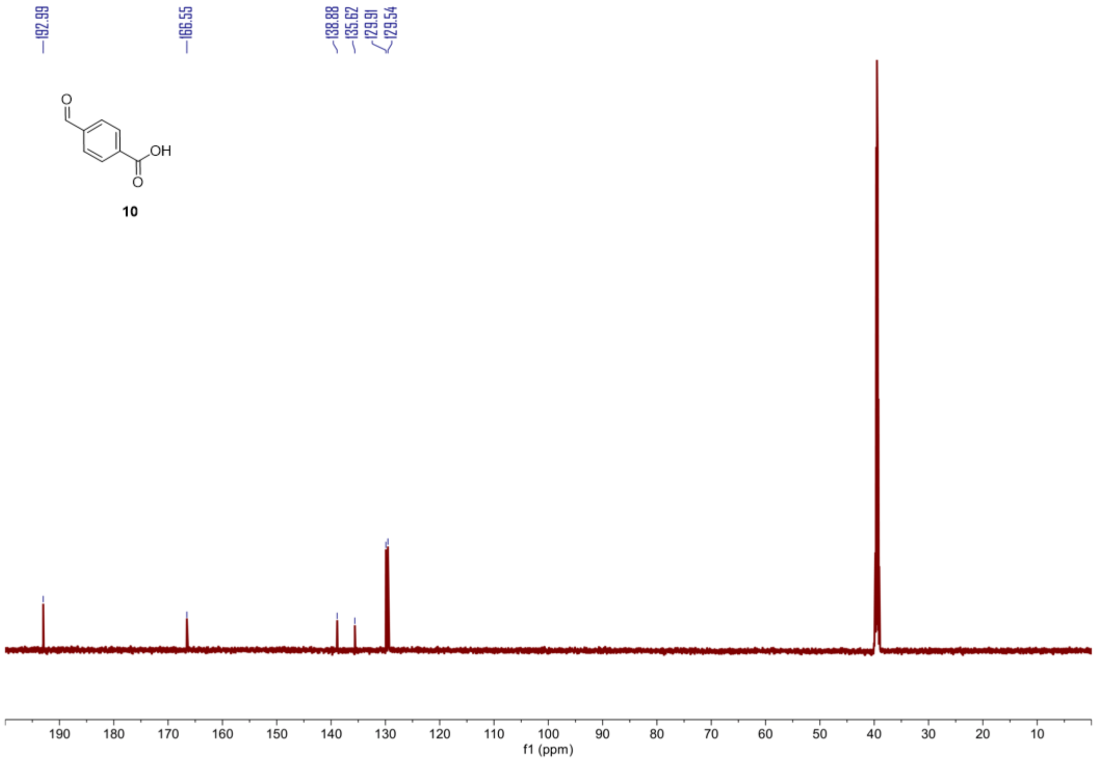

Figure S34. ^{13}C NMR (150 *MHz*, DMSO-d_6) spectrum of **Compound 10**.

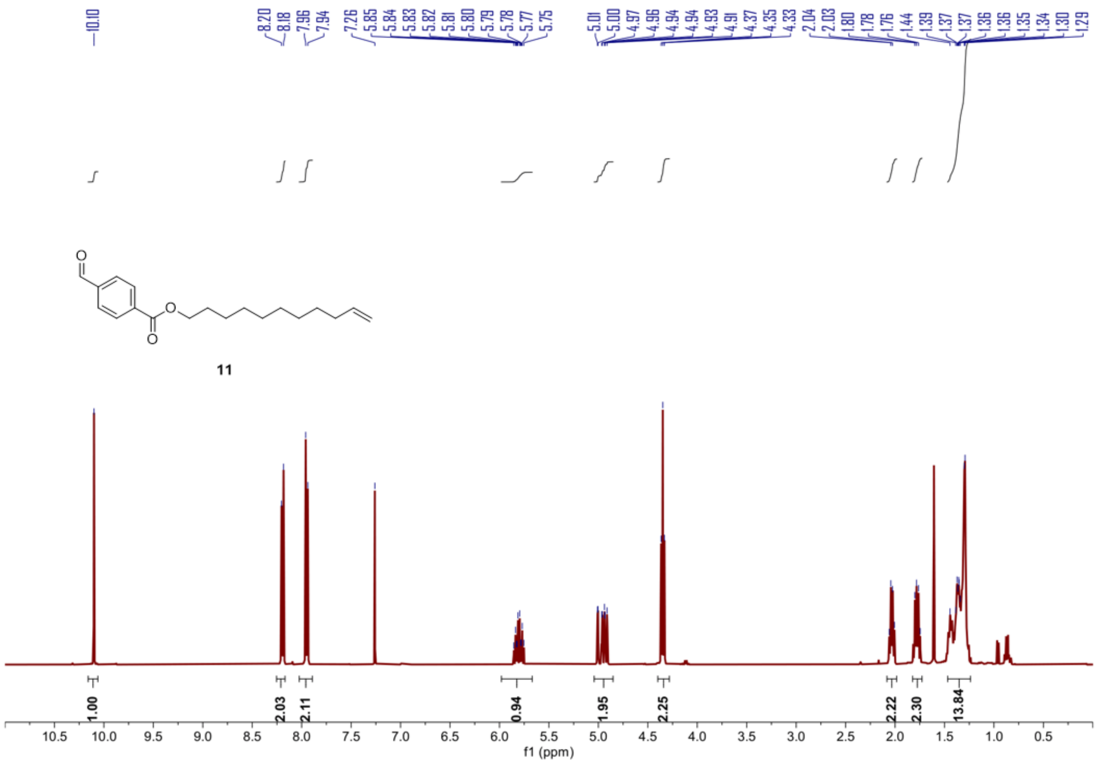

Figure S35. ^{1}H NMR (400 *MHz*, $CDCl_3$) spectrum of **Compound 11**.

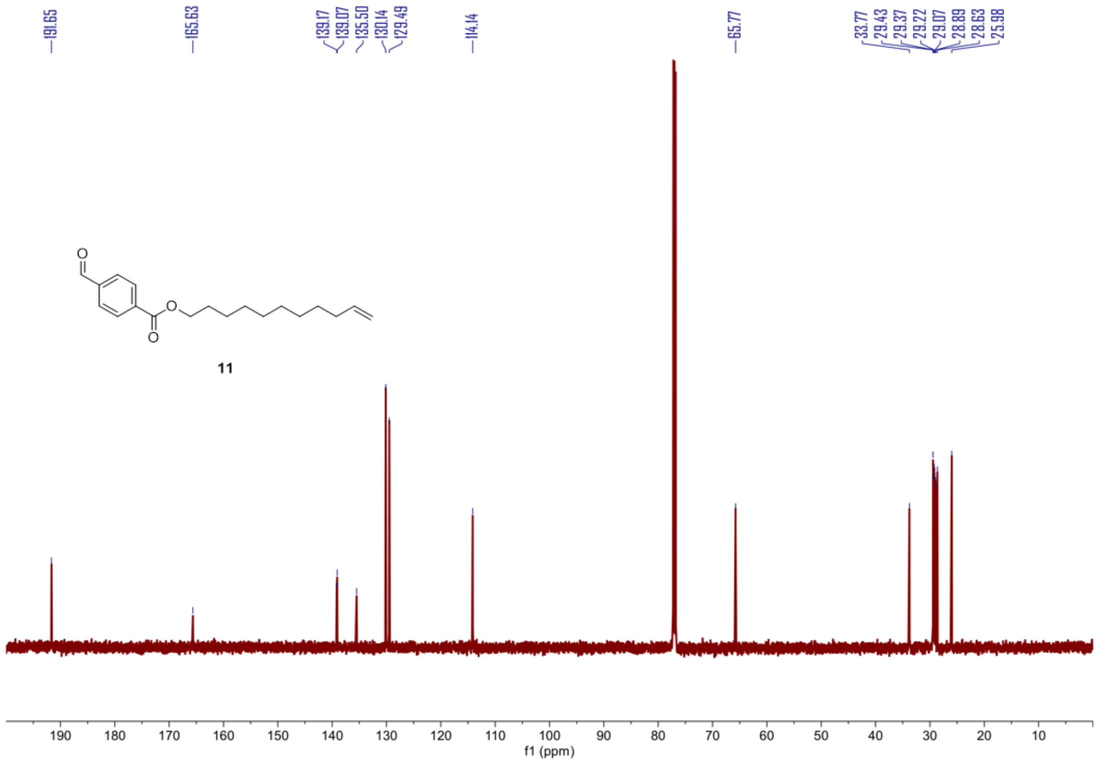

Figure S36. ^{13}C NMR (150 *MHz*, $CDCl_3$) spectrum of **Compound 11**.

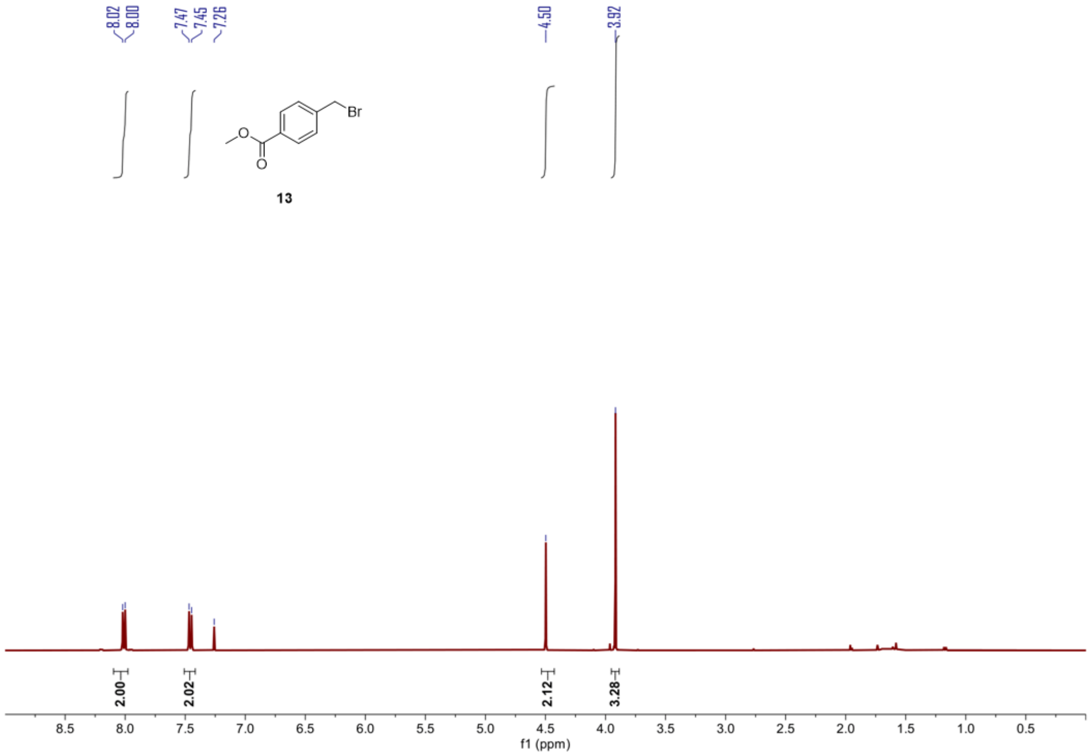

Figure S37. ^{1}H NMR (400 *MHz*, $CDCl_3$) spectrum of **Compound 13**.

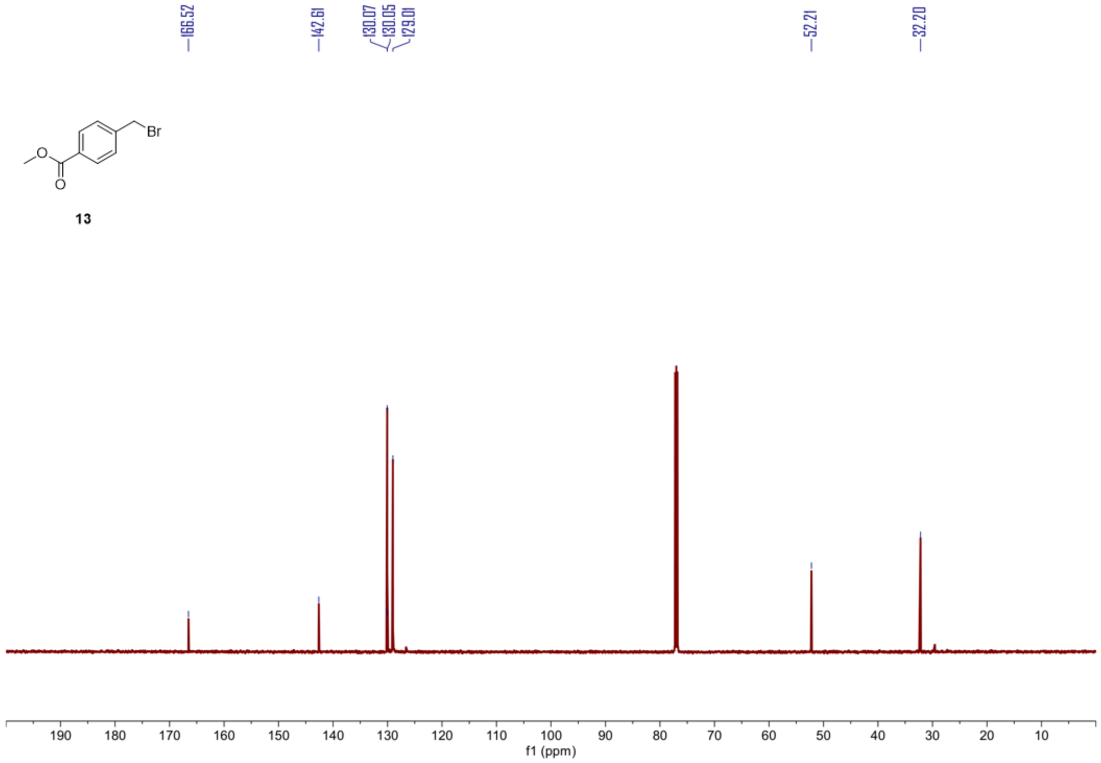

Figure S38. ^{13}C NMR (150 *MHz*, $CDCl_3$) spectrum of **Compound 13**.

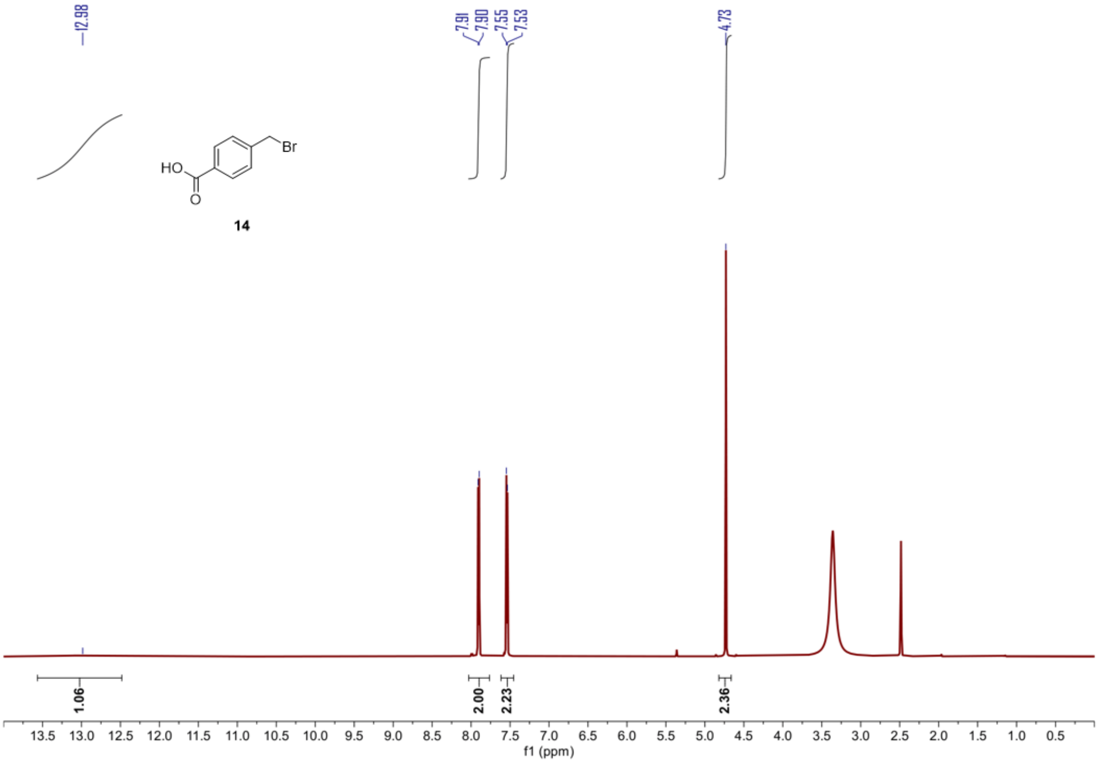

Figure S39. ^{1}H NMR (600 *MHz,* DMSO-*d*$_6$) spectrum of **Compound 14**.

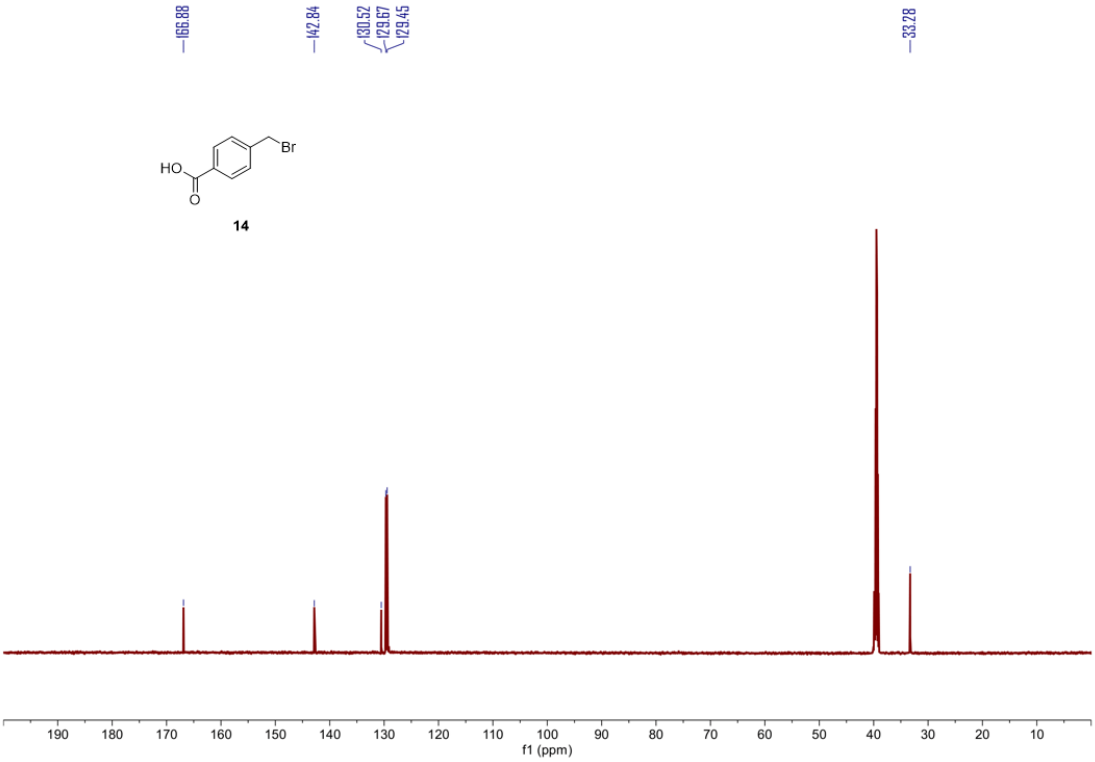

Figure S40. ^{13}C NMR (150 *MHz,* DMSO-*d*$_6$) spectrum of **Compound 14**.

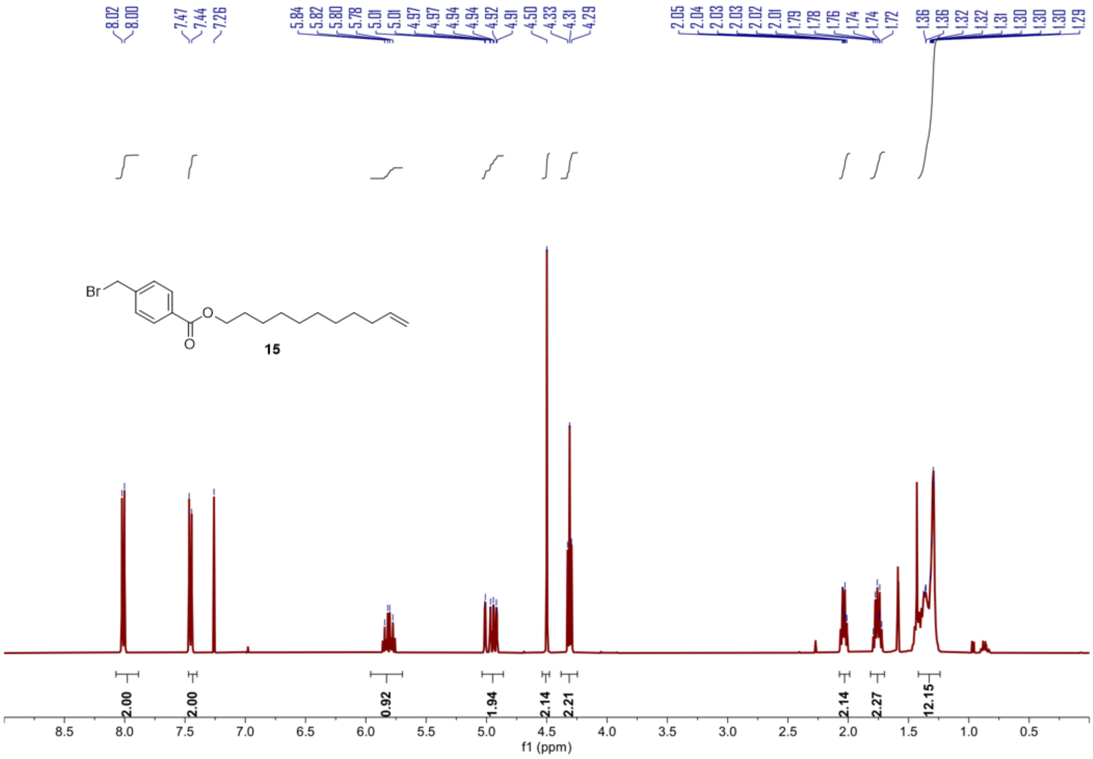

Figure S41. ^{1}H NMR (400 *MHz*, $CDCl_3$) spectrum of **Compound 15**.

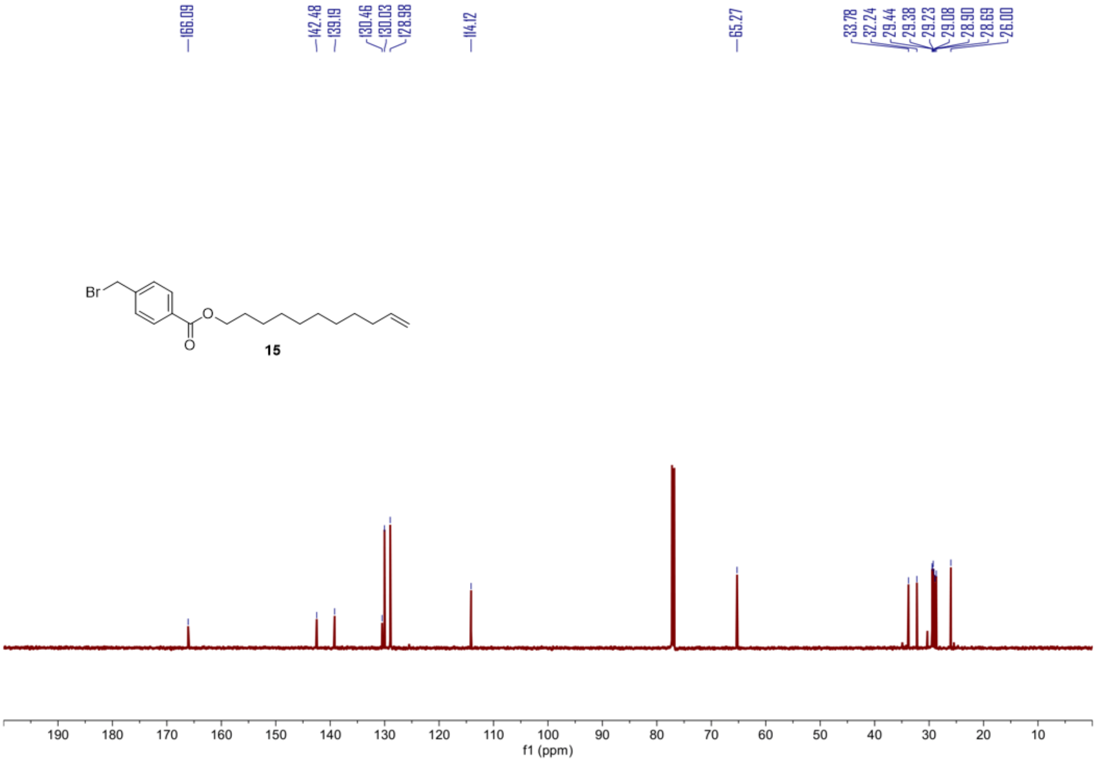

Figure S42. ^{13}C NMR (150 *MHz*, $CDCl_3$) spectrum of **Compound 15**.

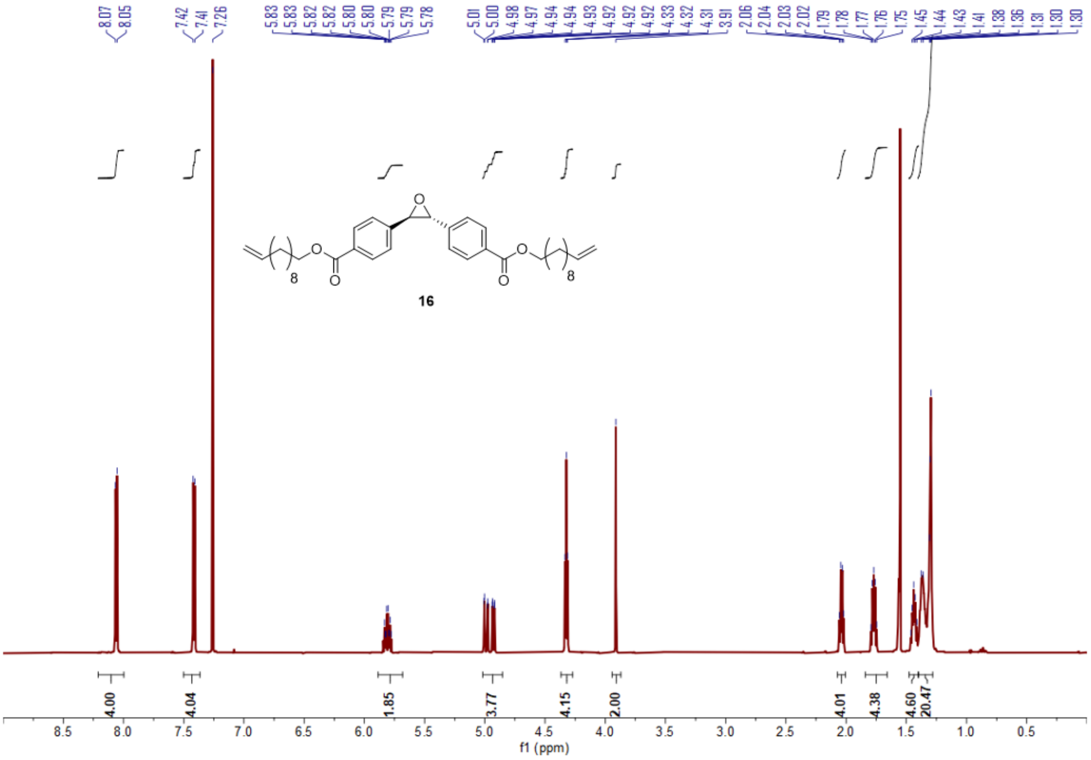

Figure S43. ^{1}H NMR (600 *MHz*, $CDCl_3$) spectrum of **Compound 16**.

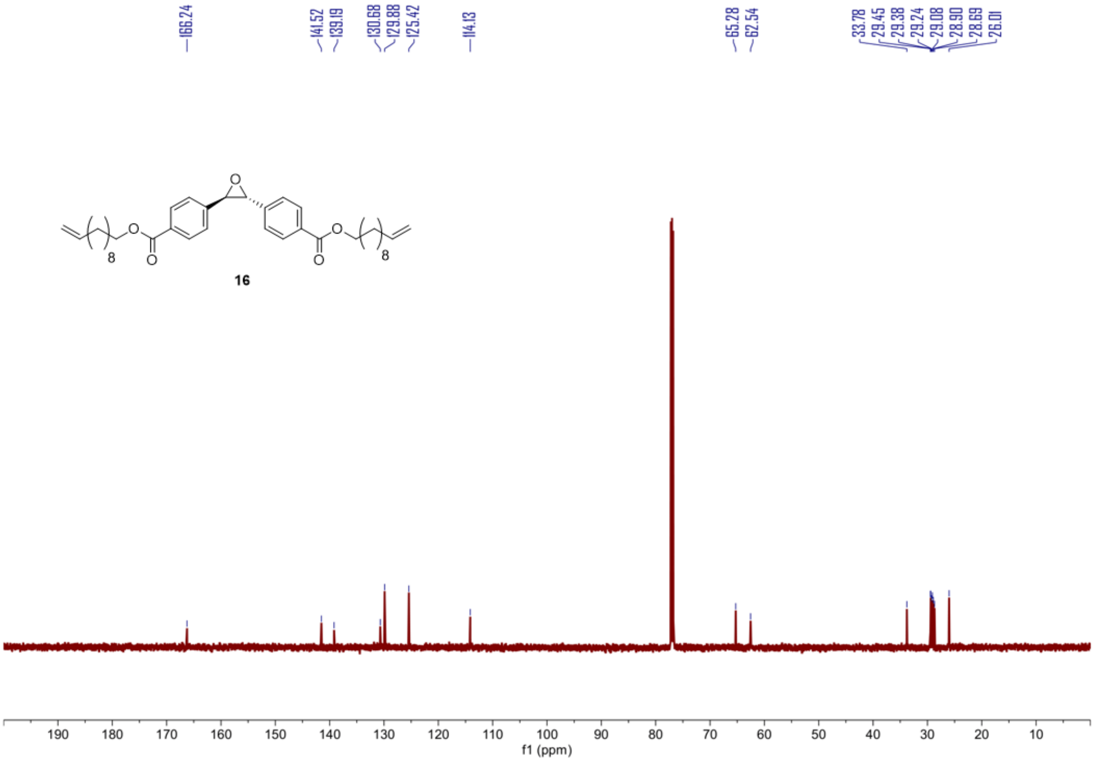

Figure S44. ^{13}C NMR (150 *MHz*, $CDCl_3$) spectrum of **Compound 16**.

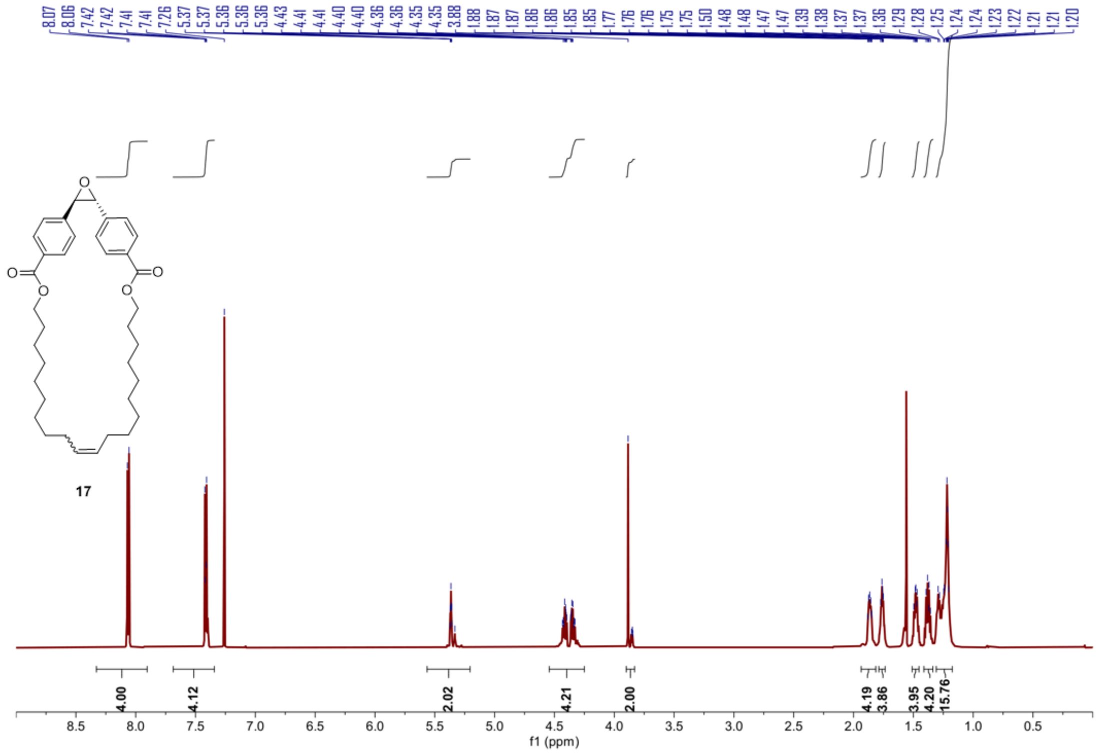

Figure S45. ^{1}H NMR (600 *MHz*, $CDCl_3$) spectrum of **Compound 17**.

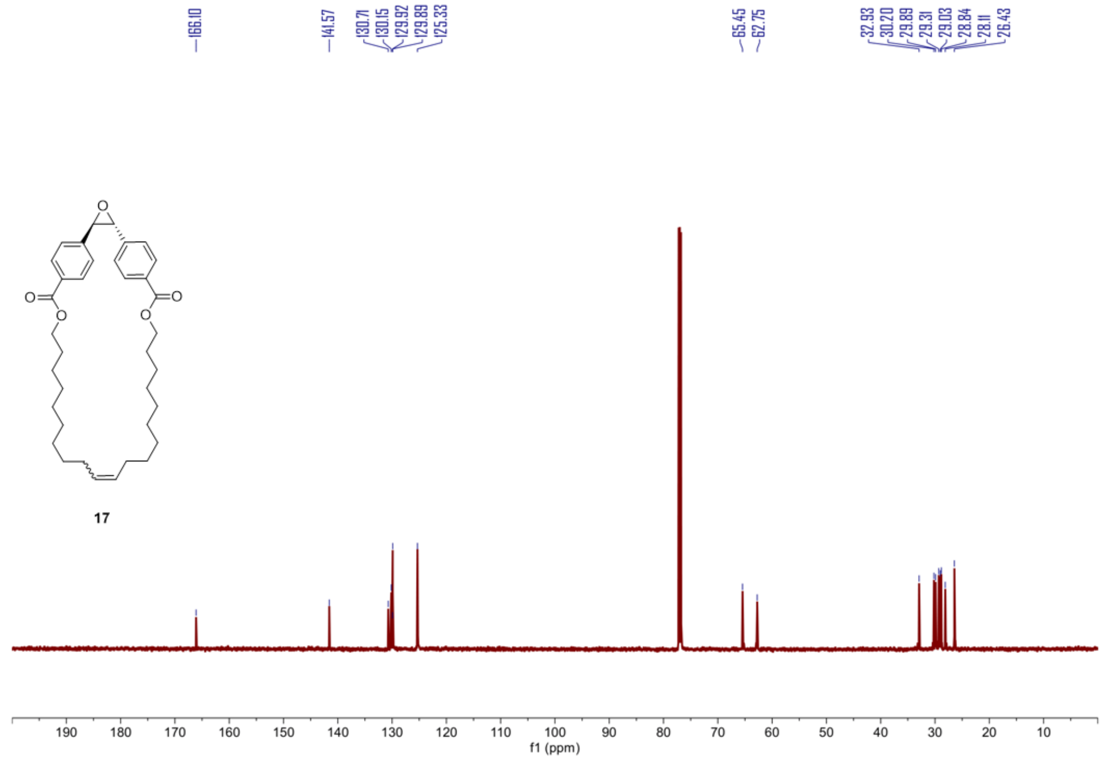

Figure S46. ^{13}C NMR (150 *MHz*, $CDCl_3$) spectrum of **Compound 17**.

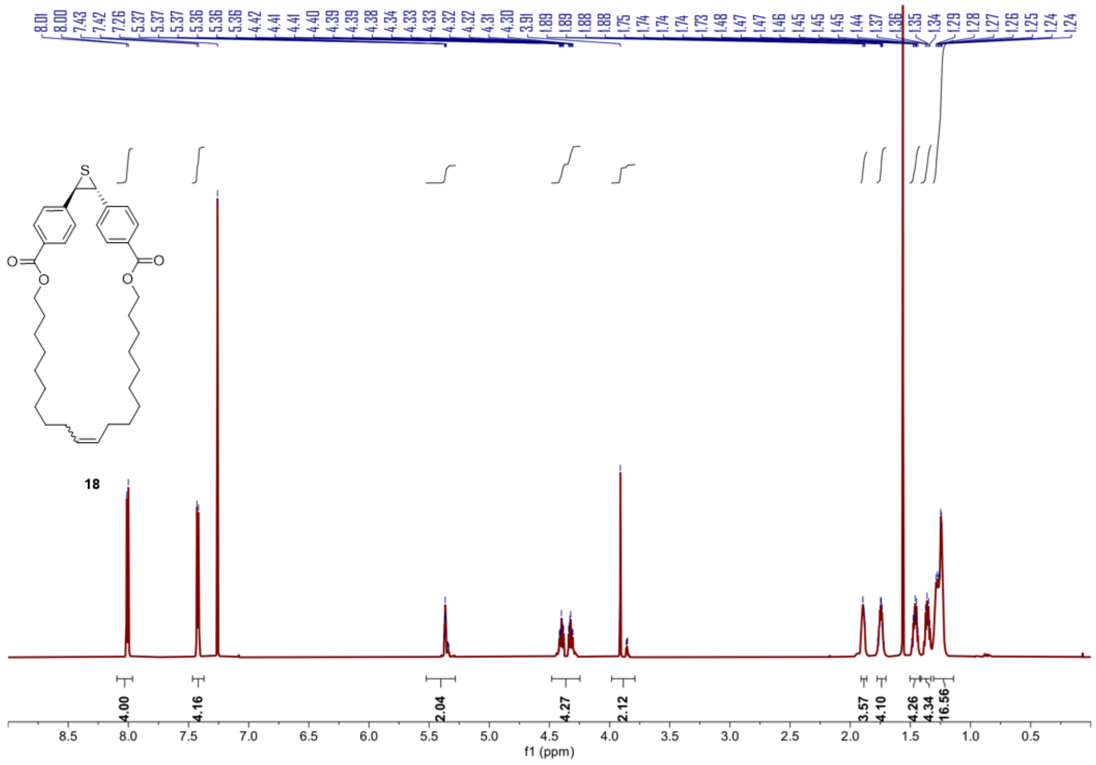

Figure S47. ^{1}H NMR (600 *MHz*, $CDCl_3$) spectrum of **Compound 18**.

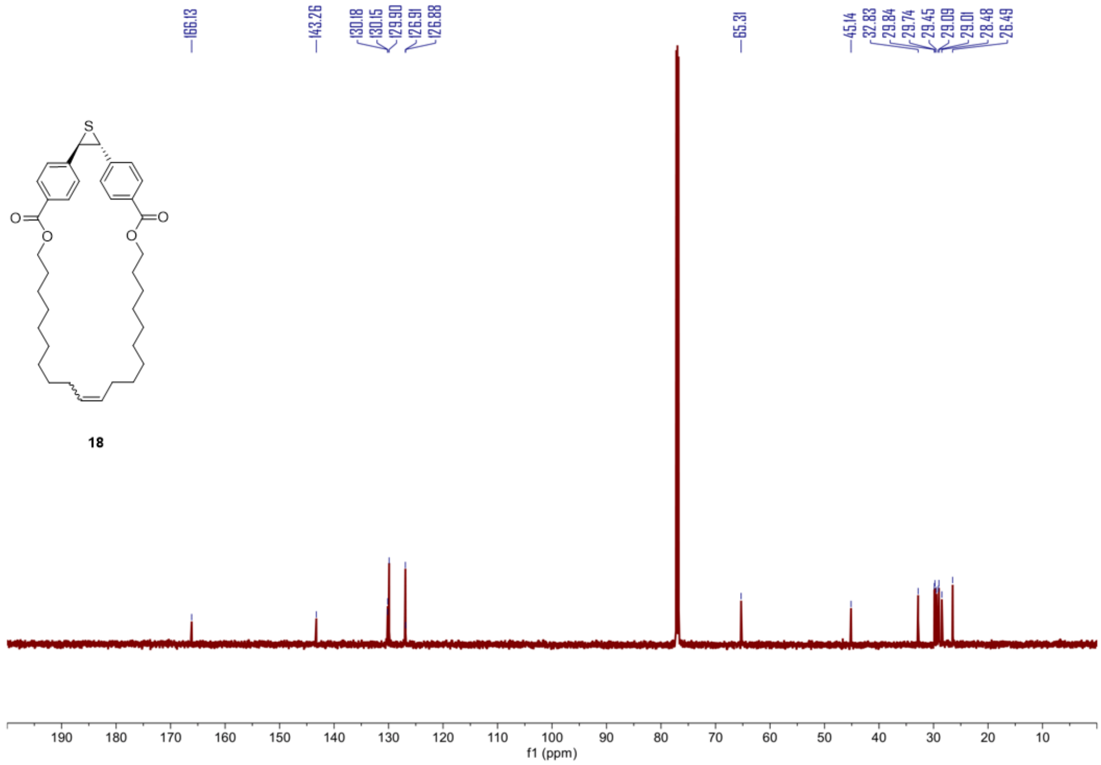

Figure S48. ^{13}C NMR (150 *MHz*, $CDCl_3$) spectrum of **Compound 18**.

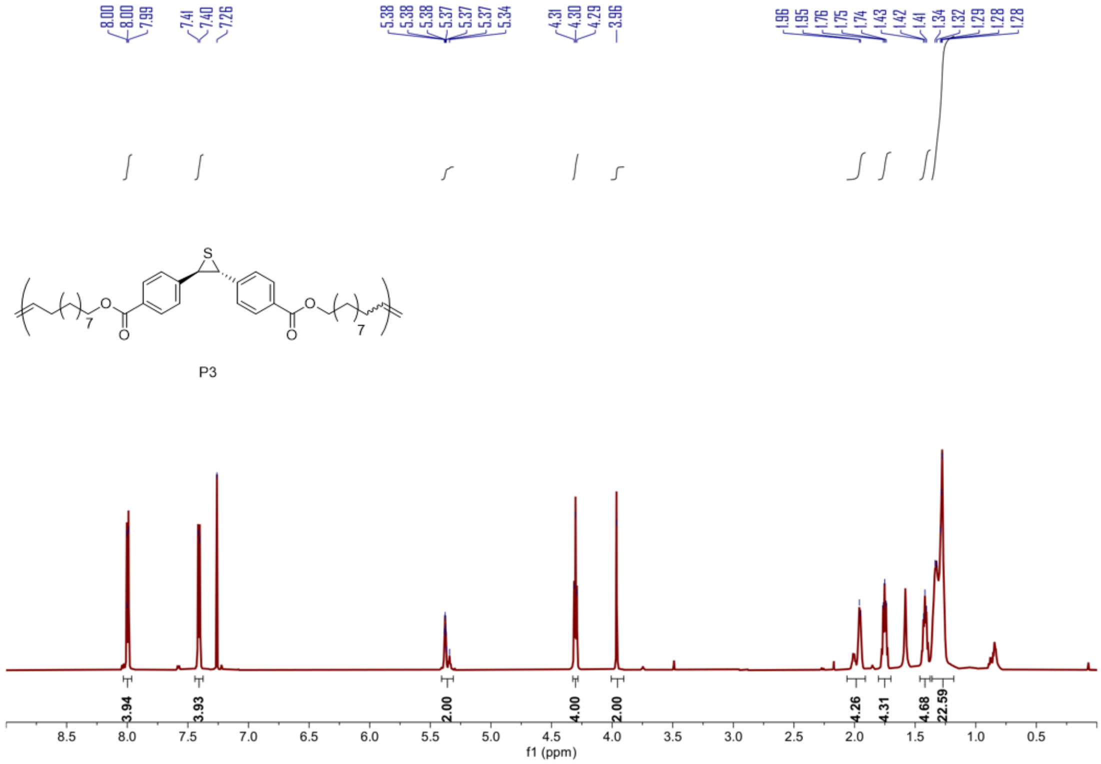

Figure S49. ^{1}H NMR (600 *MHz*, $CDCl_3$) spectrum of **P3** in $CDCl_3$. The residual solvent peaks were MeOH at 3.5 ppm and THF at 3.7ppm.

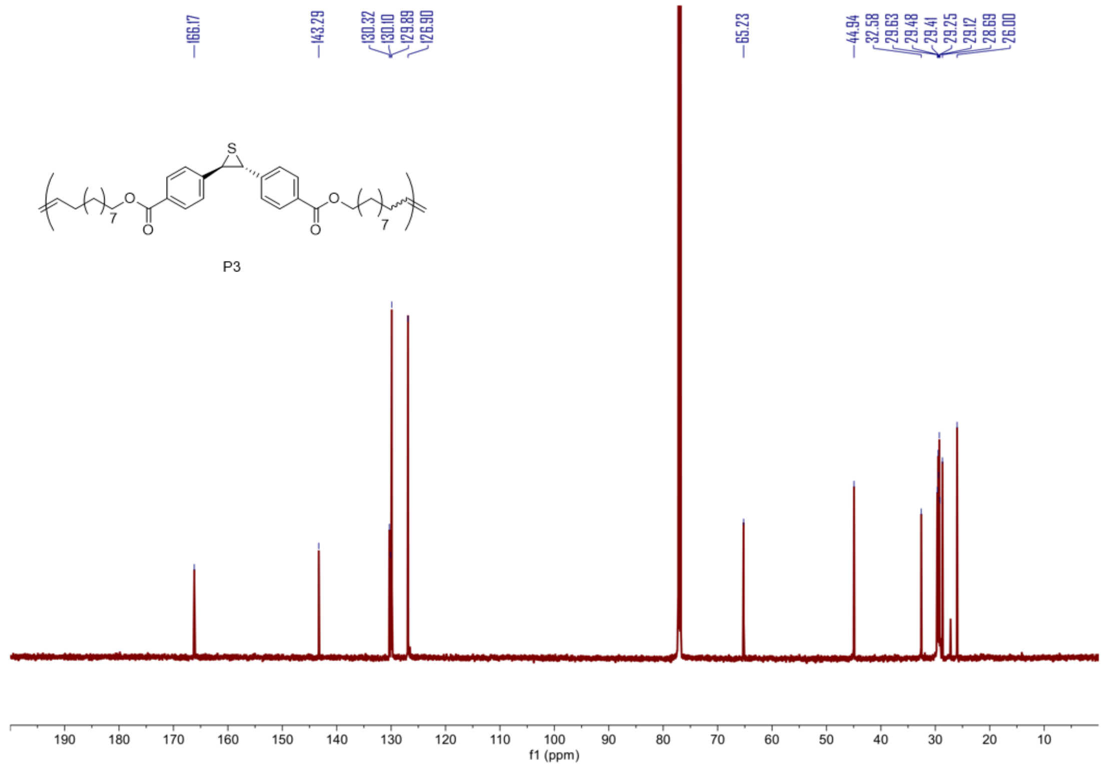

Figure S50. ^{13}C NMR (150 *MHz*, $CDCl_3$) spectrum of **P3**.

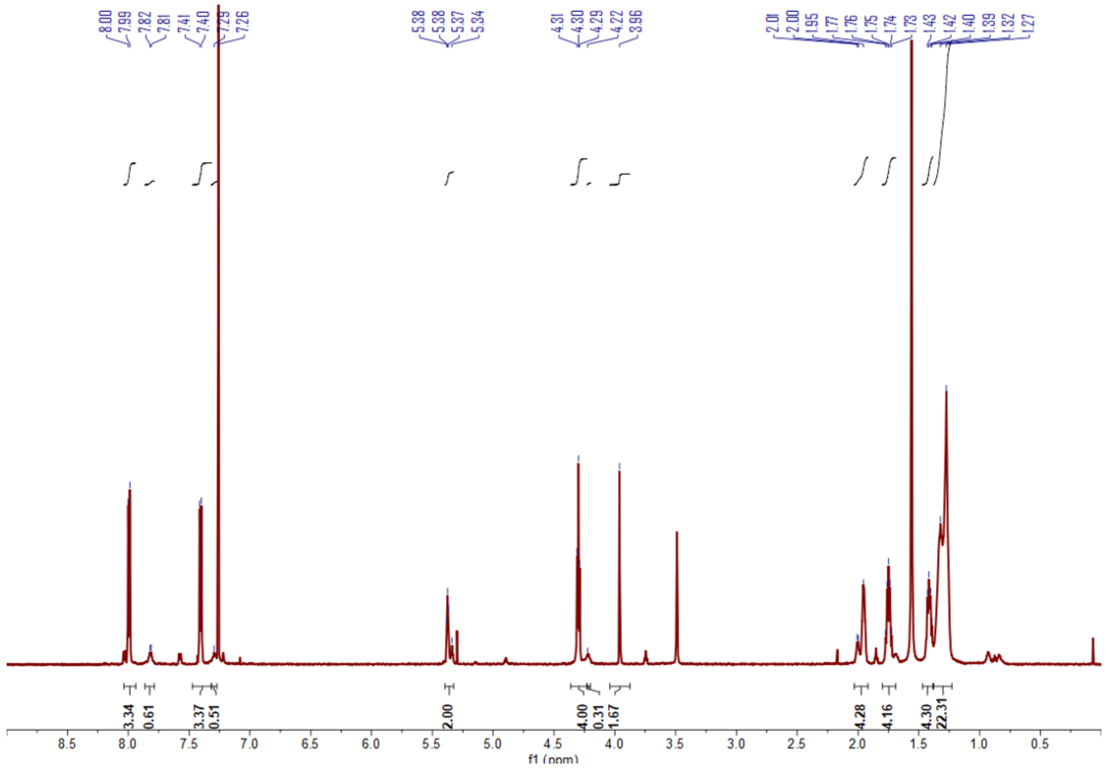

Figure S51. ^{1}H NMR (600 *MHz*, $CDCl_3$) spectrum of **P3-1**.

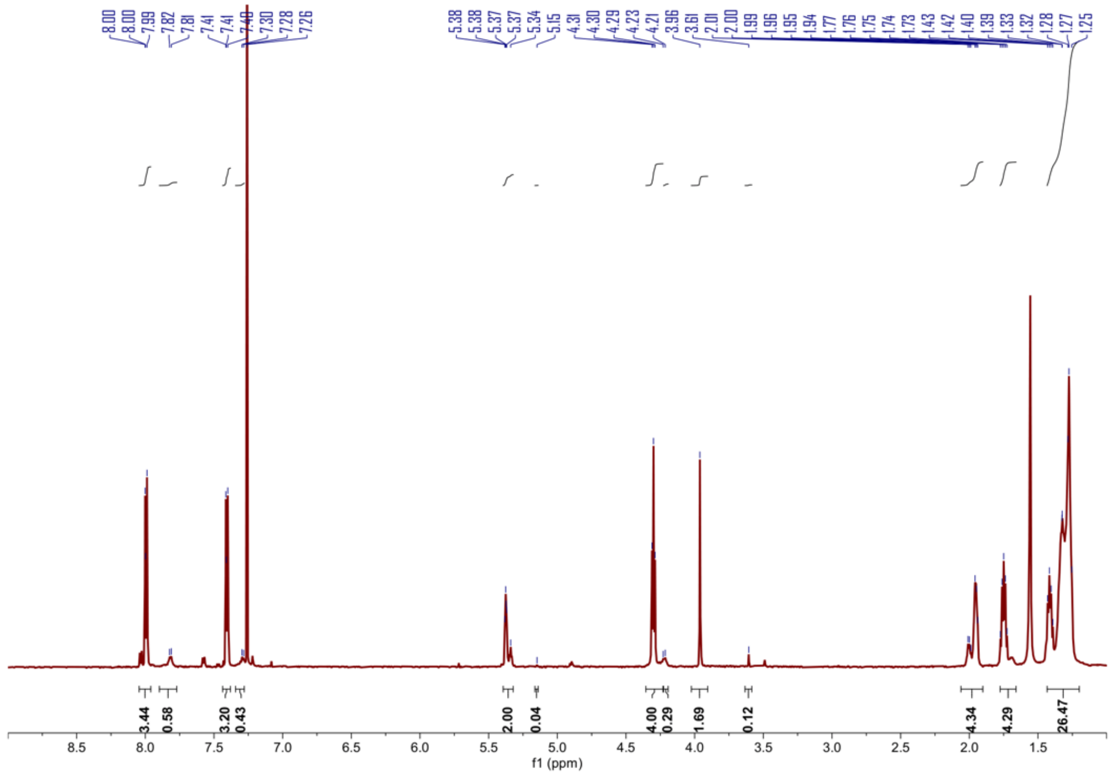

Figure S52. ^{1}H NMR (600 MHz, $CDCl_3$) spectrum of **P3-2**. The residual solvent peaks were MeOH at 3.5 ppm.

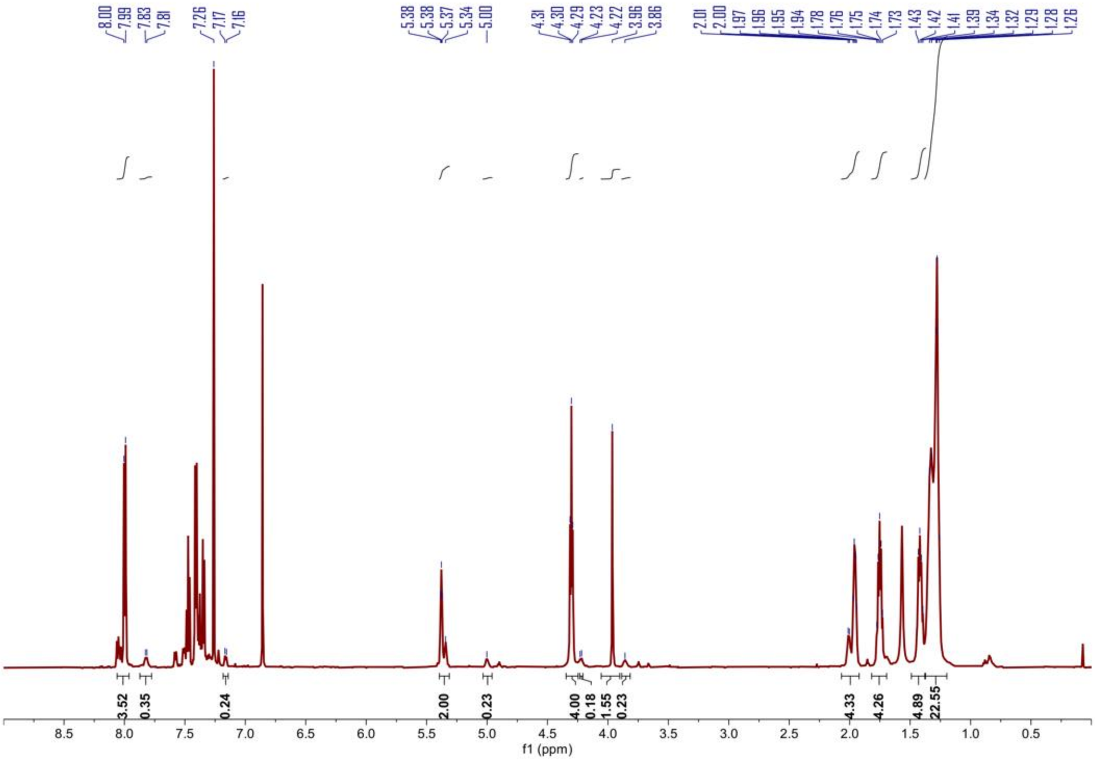

Figure S53. ^{1}H NMR (600 *MHz*, $CDCl_3$) spectrum of **P3-3** and ***N*-Phenylmaleimide**.

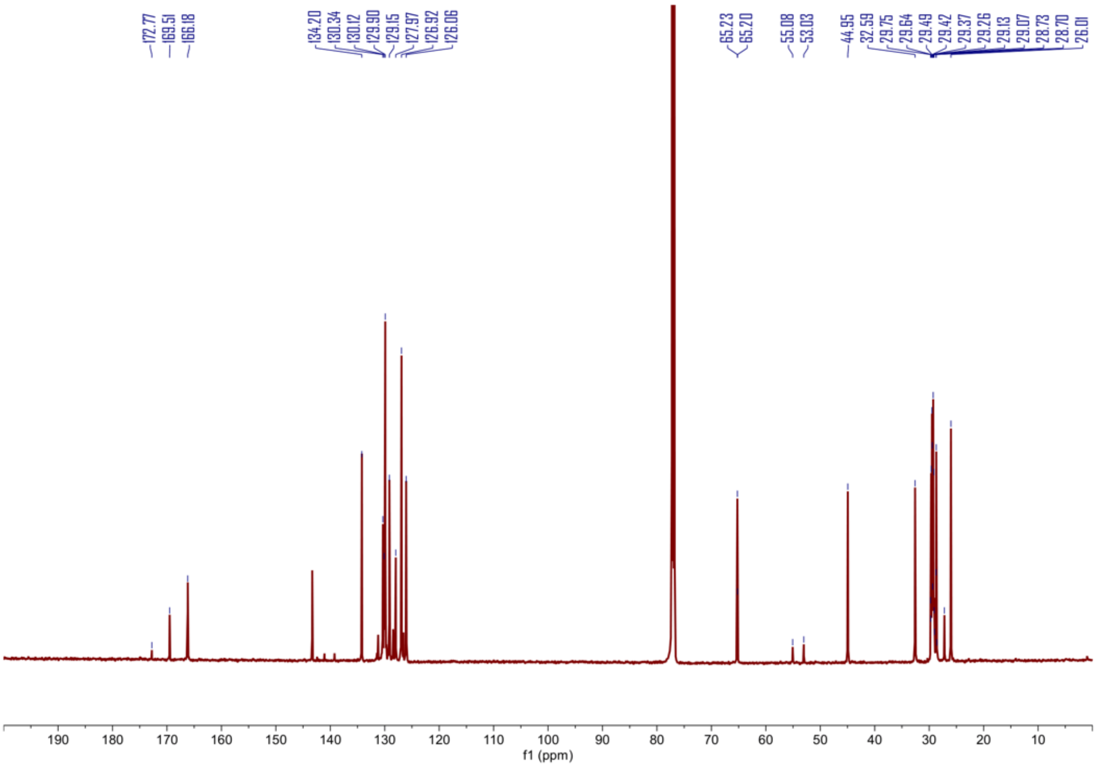

Figure S54. ^{13}C NMR (150 *MHz*, $CDCl_3$) spectrum of **P3-3** and ***N*-Phenylmaleimide**.

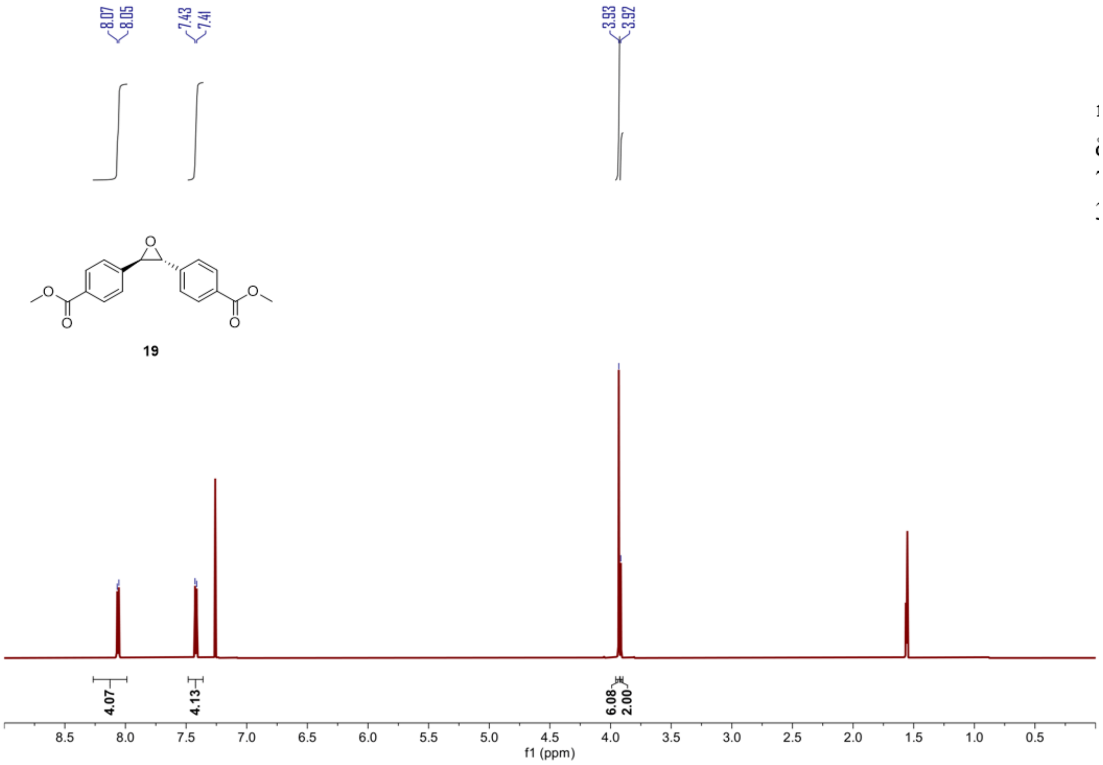

Figure S55. ^{1}H NMR (600 *MHz*, $CDCl_3$) spectrum of **Compound 19**.

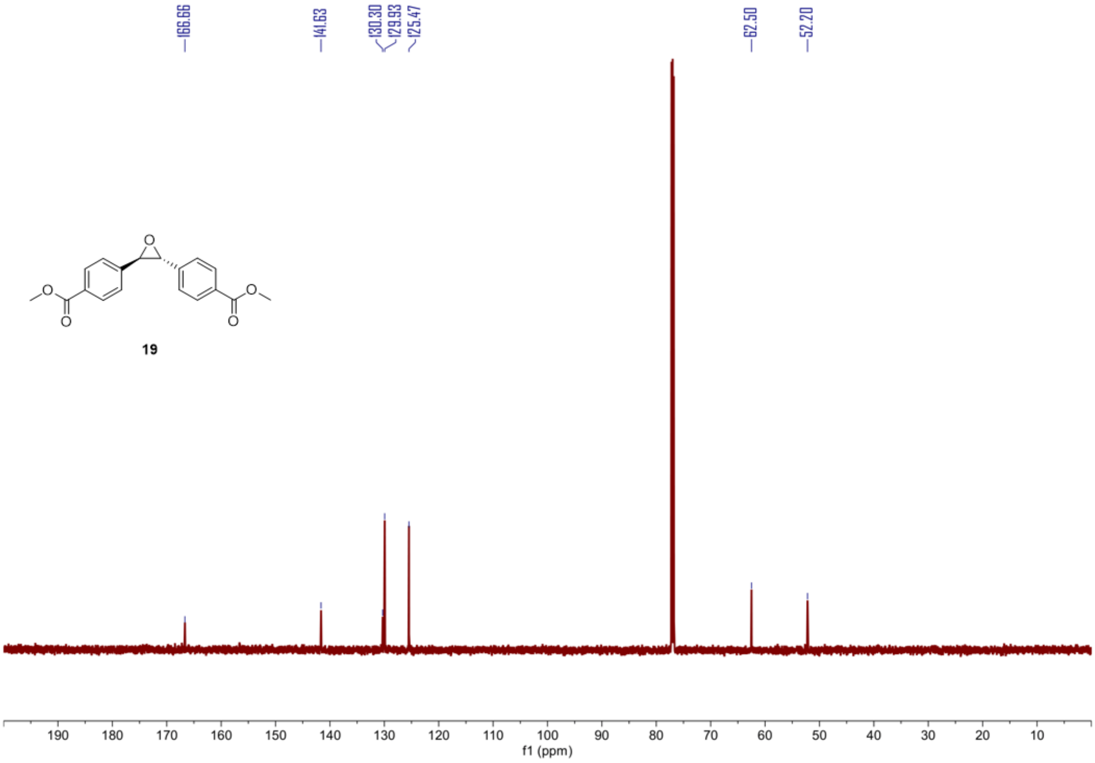

Figure S56. ^{13}C NMR (150 MHz, $CDCl_3$) spectrum of **Compound 19**.

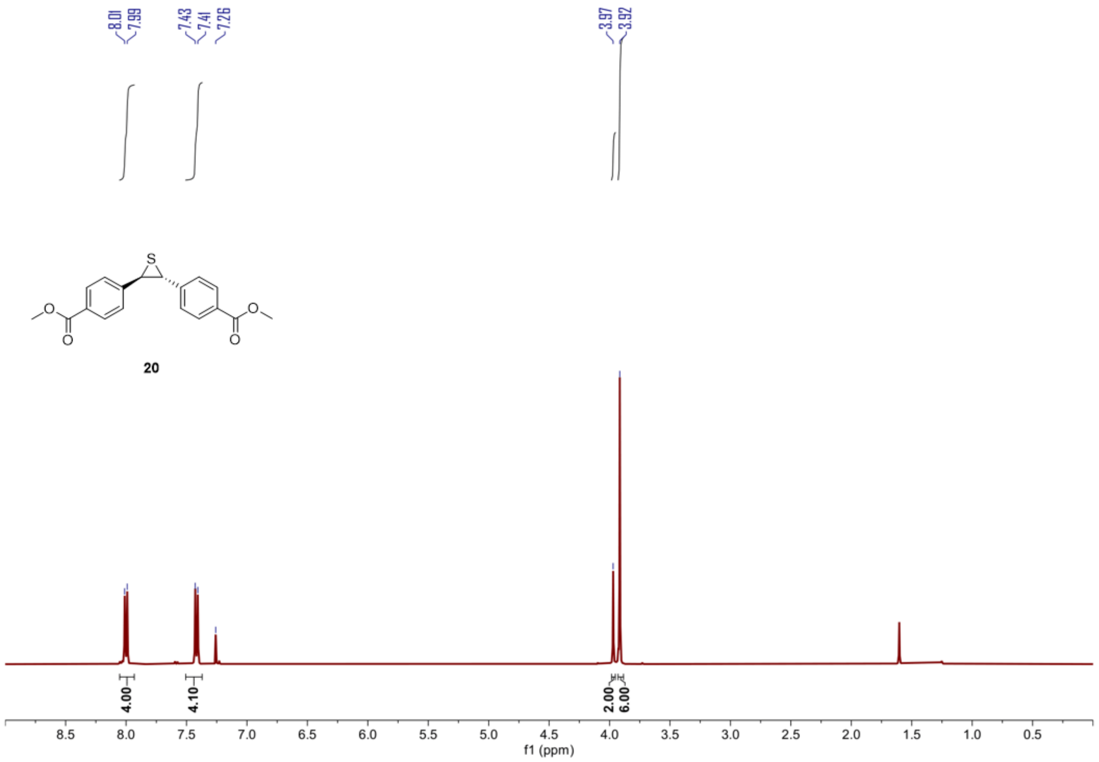

Figure S57. ^{1}H NMR (400 *MHz*, $CDCl_3$) spectrum of **Compound 20**.

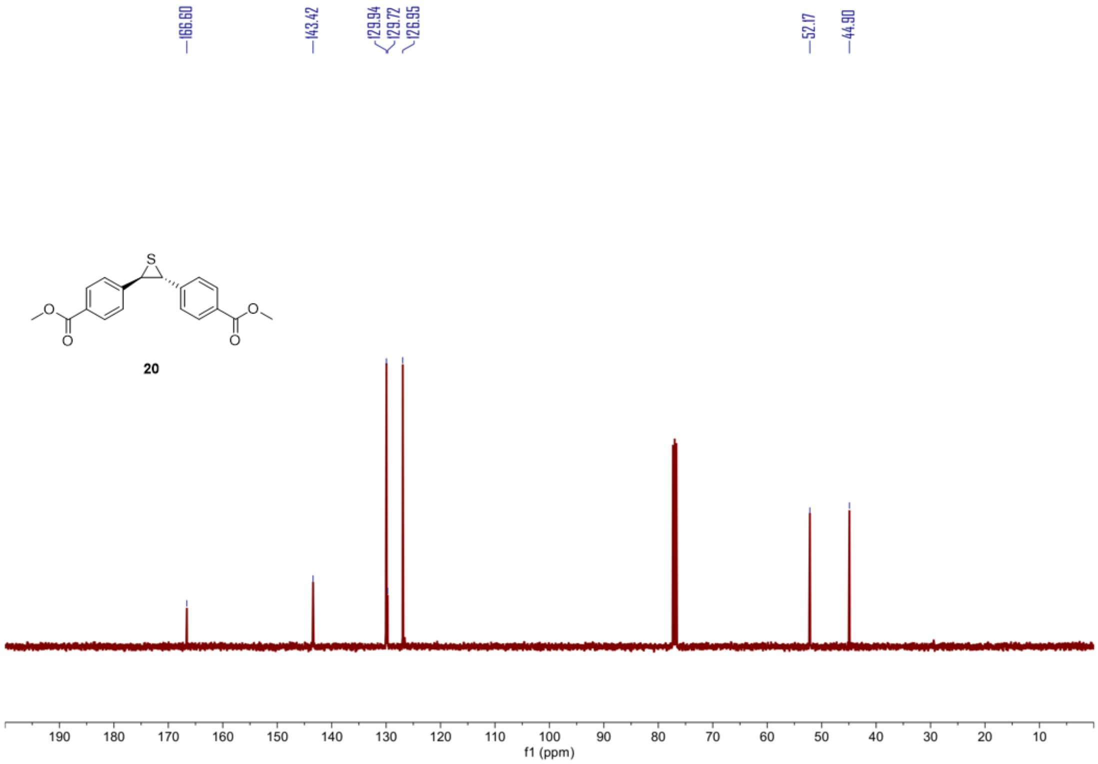

Figure S58. ^{13}C NMR (100 *MHz*, $CDCl_3$) spectrum of **Compound 20**.

Reference

(1) Klein, I. M.; Husic, C. C.; Kovács, D. P.; Choquette, N. J.; Robb, M. J. Validation of the CoGEF Method as a Predictive Tool for Polymer Mechanochemistry. *J. Am. Chem. Soc.* **2020**, *142* (38), 16364-16381.

(2) Beyer, M. K.; Clausen-Schaumann, H. Mechanochemistry: the mechanical activation of covalent bonds. *Chem. Rev.* **2005**, *105* (8), 2921-2948.
(3) Kurra, Y.; Odoi, K. A.; Lee, Y.-J.; Yang, Y.; Lu, T.; Wheeler, S. E.; Torres-Kolbus, J.; Deiters, A.; Liu, W. R. Two rapid catalyst-free click reactions for in vivo protein labeling of genetically encoded strained alkene/alkyne functionalities. *Bioconjug. Chem.* **2014**, *25* (9), 1730-1738.
(4) Chan, T. H.; Finkenbine, J. R. Facile conversion of oxiranes to thiiranes by phosphine sulfides. Scope, stereochemistry, and mechanism. *J. Am. Chem. Soc.* **1972**, *94* (8), 2880-2882.
(5) Methcohn, O.; Moore, C.; Taljaard, H. C. A Stereocontrolled Approach to Electrophilic Epoxides. *J. Chem. Soc., Perkin Trans. 1* **1988**, 2663-2674.
(6) Liu, C.; Xu, L.; Chi, D.; Li, Y.; Liu, H.; Wang, J. Synthesis of novel acceptor molecules of mono-and multiadduct fullerene derivatives for improving photovoltaic performance. *ACS Appl. Mater. Interfaces* **2013**, *5* (3), 1061-1069.
(7) Wang, S.; He, W.; Xiao, C.; Tao, Y.; Wang, X. Synthesis of Y-shaped OEGylated poly (amino acid) s: the impact of OEG architecture. *Biomacromolecules* **2019**, *20* (4), 1655-1666.
(8) Zhang, Z. W.; Li, H. B.; Li, J.; Wang, C. C.; Feng, J.; Yang, Y. H.; Liu, S. X. Synthesis of Epoxides from Alkyl Bromides and Alcohols with in Situ Generation of Dimethyl Sulfonium Ylide in DMSO Oxidations. *J. Org. Chem.* **2020**, *85* (2), 537-547.
(9) Klukovich, H. M.; Kean, Z. S.; Ramirez, A. L. B.; Lenhardt, J. M.; Lin, J.; Hu, X.; Craig, S. L. Tension Trapping of Carbonyl Ylides Facilitated by a Change in Polymer Backbone. J. Am. Chem. Soc. **2012**, 134 (23), 9577-9580.
